# Efficacy and safety of Chinese herbal medicine in post-stroke epilepsy: a systematic review and meta-analysis

**DOI:** 10.3389/fphar.2023.1286093

**Published:** 2023-11-21

**Authors:** Tianye Sun, Kaiyue Wang, Lili Li, Mingyuan Yan, Jing Wu, Jinmin Liu

**Affiliations:** ^1^ Beijing University of Chinese Medicine, Beijing, China; ^2^ Dongzhimen Hospital, Beijing University of Chinese Medicine, Beijing, China; ^3^ Dongfang Hospital, Beijing University of Chinese Medicine, Beijing, China

**Keywords:** post-stroke epilepsy, Chinese herbal medicine, efficacy, safety, anti-seizure, randomized controlled trials, systematic review, meta-analysis

## Abstract

**Background:** Poststroke epilepsy (PSE) is a common complication of strokes that seriously affects the recovery and quality of life of patients, and effective treatments are needed. Chinese herbal medicine (CHM) adjunctive therapy is a viable treatment option, but current evidence is insufficient to support its efficacy and safety. This study aimed to evaluate the efficacy and tolerability of CHM adjunctive therapy in the treatment of PSE.

**Methods:** A systematic search of eight databases was conducted to identify PSE-related randomized clinical trials from the inception of each database through October 2023. The methodological quality assessment was conducted by RoB 2.0, meta-analysis was conducted by RevMan 5.3 and Stata 15.1, and evidence quality was evaluated by GRADE.

**Results:** Twenty-three RCTs involving 1,901 PSE patients were identified. We found that orally administered CHM plus conventional Western medicine (CWM) was superior to CWM monotherapy in increasing the 75% responder rate (*RR* 1.46, 95% CI: 1.31 to 1.62, *p* < 0.00001), decreasing the seizure duration (*MD* -1.01, 95% CI: −1.30 to −0.72, *p* < 0.00001), improving total responder rate (*RR* 1.29, 95% CI: 1.20 to 1.37, *p* < 0.00001), reducing epileptiform discharges (EDs) (*MD* -2.02.46, 95% CI: −2.64 to −1.40, *p* < 0.00001), and decreasing the number of leads involved in epileptiform discharge (*MD* -3.92, 95% CI: −5.15 to −2.68, *p* < 0.00001). Furthermore, intravenously administered CHM plus CWM was superior regarding 75% responder rate (*RR* 1.39, 95% CI: 1.24 to 1.56, *p* < 0.00001), total responder rate (*RR* 1.29, 95% CI: 1.20 to 1.39, *p* < 0.00001), EDs (*MD* -3.92, 95% CI: −5.15 to −2.68, *p* < 0.00001), and the number of leads involved in epileptiform discharge (*MD* -1.82, 95% CI: −2.62 to −1.02, *p* < 0.00001). However, regarding the 50%–75% responder rate, there was no statistically significant difference between the two groups for either oral (*RR* 1.00, 95% CI: 0.77 to 1.29, *p* = 0.98) or injectable CHM (*RR* 0.95, 95% CI: 0.67 to 1.33, *p* = 0.75). Both orally administered CHM plus CWM (*RR* 0.56, 95% CI: 0.35 to 0.90, *p* = 0.02) and intravenously administered CHM plus CWM (*RR* 0.64, 95% CI: 0.45 to 0.90, *p* = 0.010) caused fewer AEs than CWM. Furthermore, the levels of evidence ranged from low to high due to publication bias and heterogeneity.

**Conclusion:** CHM adjuvant therapy may be an effective and safe therapy for PSE. However, due to the poor quality of clinical data, more well-designed RCTs are needed to confirm these findings.

**Systematic Review Registration**: https://www.crd.york.ac.uk/PROSPERO/display_record.php?RecordID=364356, identifier PROSPERO (CRD42022364356)

## 1 Introduction

Poststroke epilepsy (PSE) is defined as seizures occurring within a certain period of time after stroke, with no history of epilepsy before stroke and exclusion of brain and systemic diseases, and electroencephalogram monitoring of epileptic discharges consistent with the site of the stroke lesion (Myint et al., 2006; Pitkänen et al., 2016). PSE, the primary complication of stroke, accounts for approximately 2.7%–12% of all causes of epilepsy in countries worldwide (WHO, 2019), especially in patients over 60 years of age with newly diagnosed epilepsy, with stroke as the cause as high as 40%–55% (Silverman et al., 2002; Zou et al., 2015). PSE seriously affects the treatment and rehabilitation of stroke, leading to decreased quality of life, prolonged hospitalization, and increased healthcare costs. PSE also induces or aggravates other complications like cognitive impairment and may lead to recurrent stroke and death, placing a substantial burden on the national healthcare system.

The exact mechanism of PSE is unclear. Studies suggest a close relationship with a series of pathophysiological changes secondary to stroke, such as chronic inflammation, angiogenesis, neurodegeneration, neurogenesis, selective neuronal loss, synaptic plasticity, and glial scar. PSE is treated mainly with drugs (Johnson and Kaminski, 2020), but due to the complex pathophysiological mechanisms of PSE, there is still a lack of sufficient evidence-based recommendations on the principles of drug selection for PSE. More importantly, even with the active cooperation of patients, the control rate is ideally only about 70%, while 30% of patients are still not effectively controlled (Kwan et al., 2009), resulting in poor compliance and susceptibility to drug resistance. Additionally, the combination of multiple anti-seizure medications (ASMs) will cause more functional damage and increase the risk of sudden unexpected death in epilepsy (Harden et al., 2017; Hou et al., 2022). Therefore, safer and more effective treatment strategies are urgently needed. Chinese herbal medicine (CHM) has the remarkable advantage of multi-target and multi-pathway intervention in diseases and has shown significant efficacy and safety in the treatment of stroke, epilepsy, and even refractory epilepsy. Several medical basic studies have demonstrated that CHM could significantly improve cerebral ischemic injury after stroke (Jiang and Hu, 2022; Ren et al., 2022; Zhu et al., 2020). In addition, in epilepsy treatment, CHM could not only reduce seizure frequency but also improve emotional disorder and cognitive impairment (Ping et al., 2019; Mishra et al., 2021). With the in-depth study of the mechanism, a growing number of antiepileptic effects of single CHM, CHM extracts and Chinese compound formula have been revealed. Studies have shown that CHM exert neuroprotective effects mainly through anti-inflammatory, improving oxidative stress, inhibiting excitatory neurotransmitters, increasing inhibitory neurotransmitters, regulating ion channels, and decreasing neuroglia cell activation (Lin and Hsieh, 2021; Chen et al., 2022; Wu et al., 2023).

Clearly, CHM adjunctive therapy may be an effective and safe pharmacological therapy to improve PSE (Su et al., 2023). However, no meta-analysis has been conducted in recent years to evaluate the efficacy and safety of CHM adjunctive therapy for PSE. Therefore, to further confirm the efficacy and safety of the treatment, we conducted a comprehensive evaluation of the available clinical evidence on the latest randomized clinical trial (RCT) data of CHM combined with classical and new ASMs for PSE.

## 2 Materials and methods

### 2.1 Registration

The protocol for this systematic review and meta-analysis was registered in PROSPERO (No. CRD42022364356), and followed the Preferred Reporting Items for Systematic Reviews and Meta-Analyses (PRISMA) guidelines (Page et al., 2021).

### 2.2 Literature search

Two researchers (STY and WKY) independently searched PubMed, EMBASE, Cochrane Library, Web of Science, Chinese National Knowledge Infrastructure (CNKI), SinoMed, Chinese Science and Technique Journals Database (VIP), Wanfang Database, and two clinical trial registries (ClinicalTrials.gov and the Chinese Clinical Trial Registry) from study inception to 11 October 2023. The language restriction was English and Chinese. The search terms were “Epilepsy,” “Seizures,” “Absence,” “Stroke,” “Cerebral Infarction,” “Cerebral Hemorrhage,” “Cerebrovascular Disorders,” “Post stroke Epilepsy,” “Herbal Medicine,” “Medicine, Chinese Traditional,” “Drugs, Chinese Herbal,” “Integrated chinese and western medicine,” “Chinese patent medicine,” and related terms. We also consulted citations from relevant systematic reviews. Details of the search strategies were shown in Supplementary File S1.

### 2.3 Eligibility criteria

#### 2.3.1 Study types

Prospective parallel RCTs of Chinese herbal medicine (CHM) plus conventional Western medicine (CWM) for the treatment of PSE.

#### 2.3.2 Participants

Participants diagnosed with PSE, without gender or age restrictions. Stroke was diagnosed by Magnetic Resonance Imaging or Computed Tomography scan, and epilepsy was diagnosed by International League Against Epilepsy guidelines (Fisher et al., 2014; Fisher et al., 2017).

#### 2.3.3 Interventions

The experimental group received CHM plus CWM therapy or CHM monotherapy. For Chinese patent medicine, we only included the one whose letter of the State Drug and Food Administration approval number is “Z”.

#### 2.3.4 Comparisons

The control group received CWM alone or a combination of CHM placebo and CWM.

Both groups received the same conventional treatment for stroke, with no restrictions on the CWM, dosage, treatment duration, prescription composition, or route of administration.

#### 2.3.5 Outcomes

The primary outcomes included 75% responder rate and 50%–75% responder rate. The secondary outcomes included seizure duration; total responder rate; electroencephalogram (EEG) efficacy: epileptiform discharges (EDs), the number of leads involved in epileptiform discharge, and adverse events (AEs); as compared to baseline, respectively.

The 75% responder rate and 50%–75% responder rate were defined as the proportion of patients with a reduction in seizures frequency ≥75% and 50%–75%, as compared to baseline, respectively. In addition, the total responder rate was defined as the proportion of patients with a reduction in seizures frequency ≥50%.

### 2.4 Exclusion criteria

We excluded the following studies: 1) The intervention measures in the experimental group included other CHM treatments, such as acupuncture and moxibustion. The intervention measures in the control group included surgical treatment, deep brain stimulation, vagus nerve stimulation, responsive neurostimulation, and so on; 2) studies with incomplete or incorrect data; 3) studies involving patients with a history of epilepsy, additional epileptogenic intracranial pathology, psychiatric disorders, or central nervous system infection; 4) studies without full-text availability.

### 2.5 Data extraction

Two researchers (WKY and LLL) independently screened the titles, abstracts, and full texts of the retrieval studies according to the eligibility criteria, then independently extracted the data of the finally included literature. Disagreements were resolved by mutual negotiation or the third researcher (STY). The following information was extracted: general information (name of the author, publication year), participants’ characteristics (sample size, age, gender, duration of disease), details of interventions (ways of using CHM, dose and type of CWM used, botanical drugs in prescriptions, duration of treatment), and outcomes.

### 2.6 Risk of bias assessment

Two researchers (YMY and WJ) independently assessed the methodological quality of the included studies using the Cochrane Risk of Bias Tool 2.0 (RoB 2.0) (Sterne et al., 2019), which contains six aspects: randomization process, deviations from the intended intervention, missing outcome data, measurement of the outcome, selection of the reported results, and overall bias. Each aspect was evaluated as “low risk of bias”, “some concerns”, or “high risk of bias”. Disagreements were resolved by mutual negotiation or by consultation with a third researcher (STY).

### 2.7 Statistical analysis

All statistical analyses were conducted using Review Manager 5.4 and Stata 15.1 software. Dichotomous outcomes were expressed as the risk ratio (*RR*) with 95% confidence interval (CI), while continuous outcomes were expressed as mean difference (*MD*) with 95% CI. Heterogeneity was assessed by the *χ* ^2^ test and the *I* ^*2*^ statistic (Higgins et al., 2019). The fixed-effect model was applied in cases with low heterogeneity (*p* > 0.1, *I* ^*2*^ < 50%), and the random-effect model was used in cases with substantial heterogeneity (*p* ≤ 0.1, *I* ^*2*^ ≥ 50%) (Higgins et al., 2019; Page et al., 2021).

Sensitivity analysis was conducted by excluding individual studies to investigate the stability of the results. Subgroup analysis was conducted to investigate the potential causes of heterogeneity with two prespecified aspects: 1) treatment duration (≤3 months, >3 months); 2) types of CWM (classical ASMs, new ASMs). Furthermore, funnel plot and Egger’s test were used to evaluate the publication bias, and the trim-and-fill analysis was applied to assess whether publication bias impacted the results. Descriptive analysis was performed if the data were not suitable for meta-analysis.

### 2.8 Quality of evidence

The Grading of Recommendations Assessment, Development and Evaluatio (GRADE) system (Liu, 2022) was used to rank the quality of evidence in five domains: risk of bias, inconsistency, indirectness, imprecision, and publication bias. The quality of evidence was classified into four grades: high, moderate, low, or very low.

## 3 Results

### 3.1 Identification of studies

**FIGURE 1:**
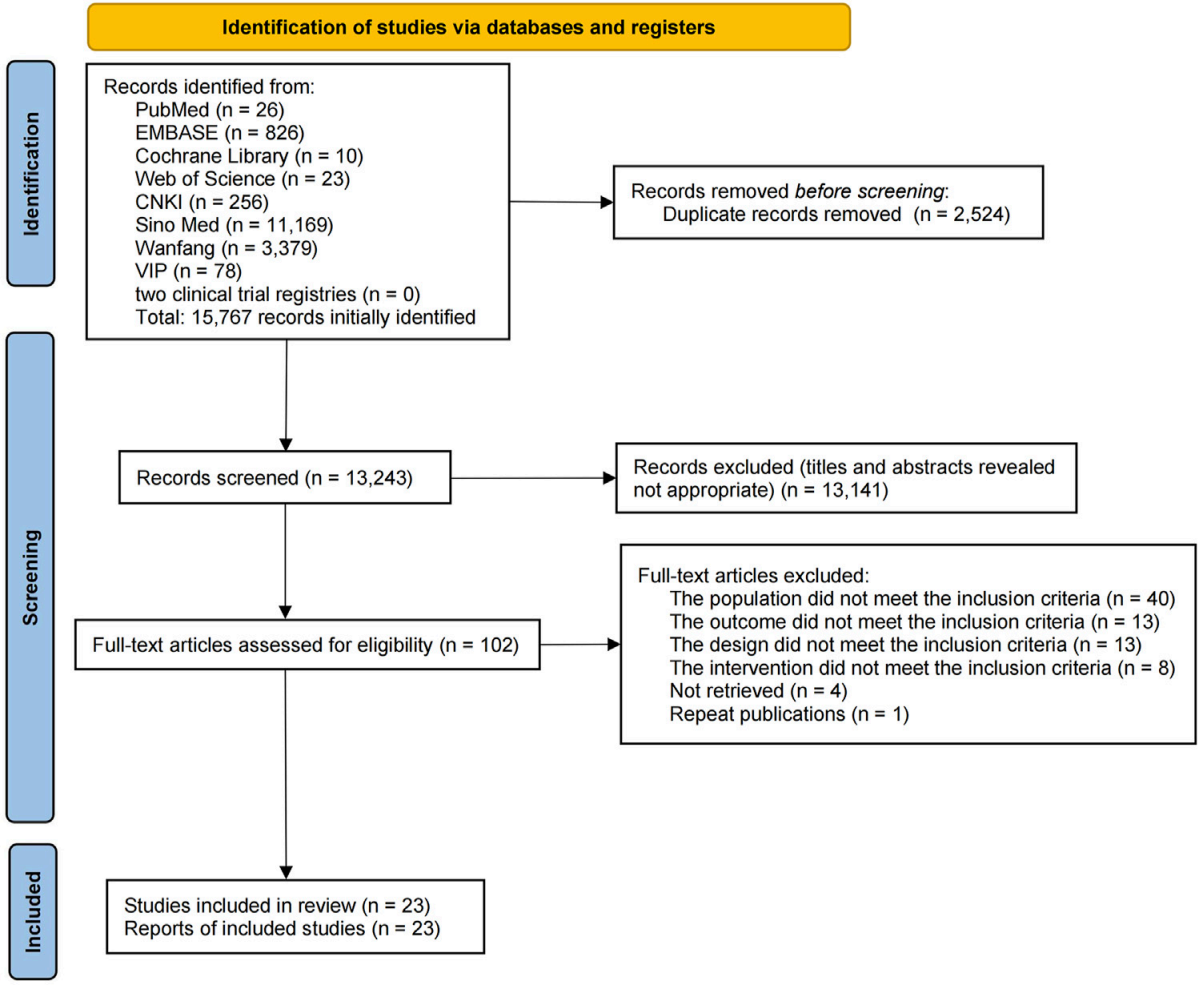
Literature selection process.

A total of 15,767 publications were retrieved from the eight databases and two clinical trial registries, and 2,524 duplicate publications were eliminated. After a review of the titles and abstracts, 13,141 publications were excluded, which left 102 publications for the secondary assessment. After reading the full text, 79 studies were eliminated (reasons for exclusion are shown in Supplementary Table S1). Eventually, 23 studies were selected (Figure 1).

### 3.2 Characteristics of the included studies

Twenty-three RCTs (Liu and Yin, 2008; Cai et al., 2013; Wang, 2014; Wang et al., 2014; Wang et al., 2015; Zhang, 2015; Guo, 2017; Wang and Zhang, 2017; Deng et al., 2018; Jiao, 2018; Li et al., 2018; Liu and Wang, 2018; Yu, 2018; Niu, 2019; Chang and Cui, 2020; Jin et al., 2020; Li and Liu, 2020; Liu et al., 2020; Zhang et al., 2020; Liu et al., 2021; Liang et al., 2022; Yang and Zhang, 2022; Zhai et al., 2022) were included in the systematic review and meta-analysis. The 23 RCTs enrolled a total of 1,901 participants (*n* = 954 and *n* = 947 from the intervention and control groups, separately), with sample sizes ranging from 18 to 63. All trials were conducted in China and published in Chinese from 2008 to 2023, and all the RCTs were two-armed and single-center trials. Both groups were based on conventional therapy, including anti-platelet aggregation, neuroprotective agents, dilation of blood vessels, etc. Fifteen studies compared CHM plus CWM with CWM and one trial compared CHM plus CWM with CWM plus placebo. The CHM was prescribed oral medicine in 15 trials (Liu and Yin, 2008; Cai et al., 2013; Wang et al., 2014; Wang and Zhang, 2017; Jiao, 2018; Li et al., 2018; Liu and Wang, 2018; Niu, 2019; Chang and Cui, 2020; Li and Liu, 2020; Liu et al., 2020; Liu et al., 2021; Liang et al., 2022; Yang and Zhang, 2022; Zhai et al., 2022), and an injection in 8 trials (Wang, 2014; Wang et al., 2015; Zhang, 2015; Guo, 2017; Deng et al., 2018; Yu, 2018; Jin et al., 2020; Zhang et al., 2020). As control interventions, two types of classical AEDs (sodium valproate, carbamazepine) and three types of new AEDs (topiramate, oxcarbazepine, levetiracetam) were used. Treatment duration ranged from 2 weeks to 12 months. Table 1 displays the characteristics of the 23 studies.

**TABLE 1:** Characteristics of the included trials.

Study	Type of stroke	Sample size (M/F); mean age (years)		Course of disease	Main CHM type	Intervention		Course of treatment	Outcomes
		T	C	T/C		T	C		
Zhai et al. (2022)	CH	38 (21/17); 61.74 ± 3.79	38 (24/14); 62.14 ± 3.87	NR	Decoction	XZD + BBTD, 150 mL, bid, po + C	OXC, 150 mg, bid for the first week, then 300 mg, bid, po	3 m	①③
Liang et al. (2022)	CI	32 (21/11); 64.53 ± 9.70	31 (21/10); 64.32 ± 9.97	NR	Pills	XDAP, 5 g, tid, po + C	VPS, 0.2 g, tid, po	3 m	④⑦
Yang and Zhang, (2022)	CI	63 (35/28); 57.26 ± 7.12	62 (37/25); 56.84 ± 7.41	2.48 ± 0.12/2.54 ± 0.21 m	Decoction	HTDD, 100 mL, bid, po + C	OXC, 0.15 g, bid for the first week, then 300 mg, bid, po	3 m	①②③④
Liu et al. (2021)	CI	39 (26/13); 60.13 ± 2.82	39 (23/16); 59.47 ± 2.67	7.72 ± 1.49/7.43 ± 1.26 m	Decoction	CLMD, 200mL, bid, po + C	VPS, 500 mg, bid + LTG, 50 mg, qd, po	8 w	①③④
Jin et al. (2020)	NR	46 (26/20); 64.39 ± 7.25	47 (27/20); 65.14 ± 8.39	4.97 ± 0.74/4.85 ± 0.63 m	Injection	SV, 100 mg, qd, ivgtt + C	VPS, 15–30 mg·kg^-1^·d^-1^, po	2 w	③④⑤⑥⑦
Zhang et al. (2020)	CH + CI + CT	41 (22/19); 66.5 ± 8. 2	42 (24/18); 65.9 ± 6.8	NR	Injection	XNJ, 20 mL, qd, ivgtt + C	VPS, 500 mg, bid, po	4 w	①②④⑦
Liu et al. (2020)	CH + CI	48 (22/26); 76.03 ± 10.01	48 (21/27); 75.21 ± 9.36	8.02 ± 2.96/7.42 ± 2.51 w	Decoction	KJD, 300mL, bid, po + C	OXC, 0.15–0.4 g, tid, po	6 m	①②③④⑤⑥⑦
Chang and Cui, (2020)	NR	49 (30/19); 63.14 ± 5.89	49 (28/21); 62.78 ± 6.02	NR	Pills+Decoction	DXP + THD, 5.5 g, bid with a decoction of old green Chinese onion (10 g) and old ginger (10 g), po + C	LEV, 0.5 g, bid, po	12 m	①
Li and Liu, (2020)	NR	40 (23/17); 63.43 ± 7.41	40 (24/16); 62.93 ± 6.48	NR	Pills (CPM)	BJP, 6 g, bid, po + C	VPS, 15–30 mg·kg^-1^·d^-1^, po	8 w	①②④
Niu, (2019)	CI	43 (22/21); 57.03 ± 5.46	42 (23/19); 57.42 ± 5.39	6.97 ± 0.74/7.13 ± 0.59 m	Decoction	QWD, bid, po + C	VPS, 0.25 g, bid for the first 2 days, then 0.5–1 g, bid, po	3 m	①②④
Jiao, (2018)	CI	18 (9/9); 58.1 ± 5.2	18 (10/8); 57.5 ± 4.8	NR	Pills	XZD + BBTD, 9 g, bid, po + C	VPS, 15 mg·kg^-1^, bid + CHM Placebo, po	6 m	①②④⑦
Yu, (2018)	NR	36 (23/13); 71.9 ± 2.5	36 (22/14); 72.3 ± 3.0	5.50 ± 1.23/5.39 ± 0.98 m	Injection	XNJ, 20 mL, qd, ivgtt + C	VPS 15–20 mg·kg^-1^, tid for the first week, then 20–30 mg·kg^-1^, tid + LEV 0.25 g, bid for the first week, then 0.5–3 g, bid, po	8 w	①②④⑤⑥⑦
Deng et al. (2018)	CH + CI	45 (25/20); 60.1 ± 5.8	45 (24/21); 60.8 ± 6.4	NR	Injection	XNJ 20 mL, qd, ivgtt + C	CBZ, 0.1–0.4 g, qd-tid, po	4 w	①②④⑤⑥⑦
Li et al. (2018)	CI	38 (38); 63.68 ± 12.45	38 (19/19); 63.56 ± 12.16	NR	Decoction	QWD, 150mL, bid, po + C	VPS, 300 mg, bid for the first week, then 400–500 mg, bid, po	2 m	①②④⑦
Liu and Wang, (2018)	NR	25 (15/10); 53.28 ± 2.76	25 (16/9); 52.37 ± 2.98	6.01 ± 1.33/5.28 ± 1.28 m	Decoction	CLMD, 100mL, bid, po + C	VPS, 15–20 mg·kg^-1^ d^-1^, po	16 w	①②③④⑤⑥
Guo, (2017)	NR	41 (25/16); 55.34 ± 5.47	41 (23/18); 54.52 ± 5.23	3.18 ± 1.35/3.51 ± 1.22 years	Injection	XNJ 20 mL, qd, ivgtt + C	OXC, 300 mg, bid, po	3 m	①②④⑦
Wang and Zhang, (2017)	CI	47 (29/18); 57.8 ± 6.4	47 (30/17); 58.1 ± 6.0	1.2 ± 0.2/1.2 ± 0.3 m	Decoction	DTD, 150mL, bid, po + C	TPM, 25 mg, bid, po	6 m	①②④⑦
Wang et al. (2015)	CH + CI	40 (24/16); 60 ± 6	40 (22/18); 60 ± 7	5.4 ± 0.3/5.4 ± 0.4 years	Injection	XNJ 20 mL, qd, ivgtt + C	CBZ, 0.1 g, tid, po	4 w	①②④⑤⑥⑦
Zhang, (2015)	NR	53 (30/23); 64.2 ± 10.3	53 (28/25); 65.5 ± 11.2	NR	Injection	XNJ 20 mL, qd, ivgtt + C	CBZ, 0.1 g, tid, po	4 w	①②④⑤⑥⑦
Wang, (2014)	NR	50 (28/22); 67.09 ± 12.06	50 (30/20); 65.70 ± 11.37	NR	Injection	XNJ 20 mL, qd, ivgtt + C	CBZ, >3 years, 0.1 g, tid; ≤3 years, 0.5 mg·kg^-1^·d^-1^, po	4 w	①②④⑤⑥⑦
Wang et al. (2014)	NR	30 (13/17); 73.63 ± 9.74	30 (16/14); 74.25 ± 11.40	NR	Granule	PXG, 6 g, tid, po + C	LEV, 0.5–1.5 g, bid, po	6 m	①②④
Cai et al. (2013)	CH + CI	32 (18/14); 64.5 ± 9.6	32 (20/12); 65.2 ± 10.1	NR	Capsules (CPM)	TTC, 2 g, tid, po + C	VPS/VPS + CBZ, po	12 w	①②④⑦
Liu and Yin, (2008)	NR	60 (36/24); 60.6 ± 9.2	54 (34/20); 63.6 ± 8.4	6.2 ± 3.1/5.9 ± 2.7 years	Decoction+Capsule	HCD, 250mL, bid + HLC, 1.2–2.0 g, bid, po + C	CBZ, 0.1 g, tid, po	6 m	①④⑦

Note: bid, twice daily; BJP, baijin pills; C, control group; CBZ, carbamazepine; CH, cerebral hemorrhage; CHM: Chinese herbal medicine; CI, cerebral infarction; CLMD, chaihu longgu muli decoction; CPM, chinese patent medicine; CT, cerebral thrombosis; DTD, ditan decoction; DXP, dingxian pills; F: female; HCD, huangqi chifeng decoction; HLC, huoluo capsules; HTDD, huatan tongluo dingxian decoction; ivgtt, intravenously guttae; KJD, kangxian jiejing decoction; LEV, levetiracetam; m, month; M, male; N: no; NR, not report; OXZ, oxcarbazepine; PXG: pingxian granule; qd, once daily; QWD, qingxin wendan decoction; SRT, sustained release tablets; SV, salvianolate injection; T, treatment group; THD, tongqiao huoxue decoction; tid, thrice daily; TPM, topiramate; TTC: tiandan tongluo capsules; VPS, sodium valproate; w: week; XDAP, xiandean pills; XNJ, xingnaojing injection; XZD, xuefu zhuyu decoction; y, year; Y, yes; ①75% responder rate; ②50%–75% responder; ③seizure duration; ④total responder rate; ⑤epileptiform discharges (EDs); ⑥the number of leads involved in epileptiform discharge; ⑦adverse events.

A total of 14 prescriptions were used in all studies, involving 57 kinds of botanical drugs. The detailed information of the CHMs (e.g., composition, dosages, quality control and sources) prescribed is presented in Supplementary Table S2.

### 3.3 Quality assessment

**FIGURE 2:**
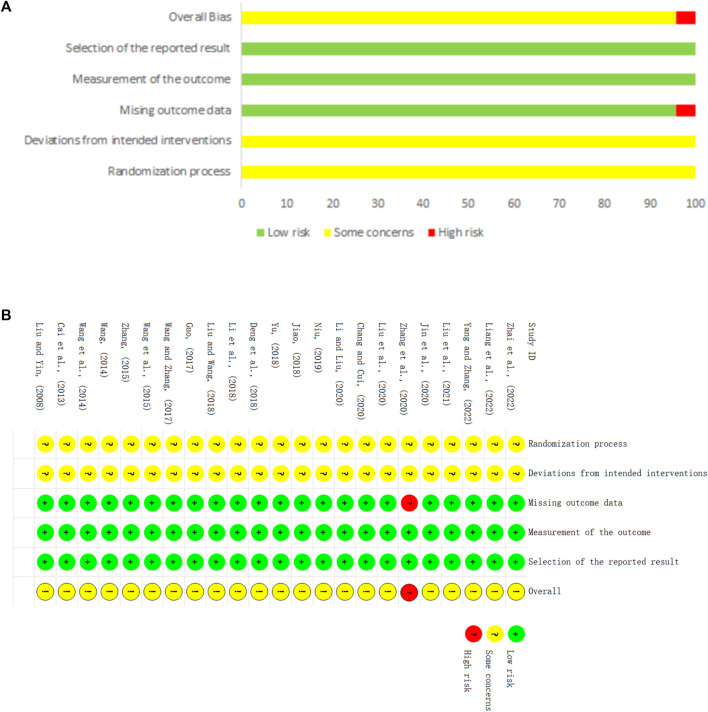
Risk of bias. **(A)** Risk of bias summary. **(B)** Risk of bias graph.

Regarding randomization, eighteen studies (Liu and Yin, 2008; Cai et al., 2013; Wang, 2014; Wang et al., 2015; Guo, 2017; Wang and Zhang, 2017; Deng et al., 2018; Jiao, 2018; Yu, 2018; Niu, 2019; Chang and Cui, 2020; Jin et al., 2020; Li and Liu, 2020; Liu et al., 2020; Zhang et al., 2020; Liang et al., 2022; Yang and Zhang, 2022; Zhai et al., 2022) provided a sufficient randomized sequence generation process. In addition, one study (Li et al., 2018) used envelopes without a specific details, and the remaining studies did not provide information on allocation concealment. Therefore we evaluated the risk of bias as unclear. Regarding deviations from the intended interventions, one study (Jiao, 2018) reported that the experimental group was treated with a combination of CHM and CWM, and the control group was treated with a combination of CHM placebo (The appearance and specifications of the placebo are approximately the same as those of the CHM) and CWM. The remaining studies did not report whether the blind method was applied for clinicians and participants, so we rated them as high risk. With respect to missing outcome data, one study (Zhang et al., 2020) reported missing visits but did not perform an intention-to-treat approach, which may affect the true outcome, leading us to rate the risk of bias as high. The remaining 22 studies had no missing data. Regarding outcome measurement, none of studies provided information on whether the blind method was utilized, but considering that the outcomes were based on objective measurement data, and the results were unlikely influenced by outcome indicator reporters, so these trials were considered with low risk. Regarding selective outcome reporting, the expected outcomes of all studies are fully reported. The risk of bias for all trials is shown in Figure 2.

### 3.4 Primary outcomes

#### 3.4.1 75% responder rate

##### 3.4.1.1 Orally administered CHM

**FIGURE 3:**
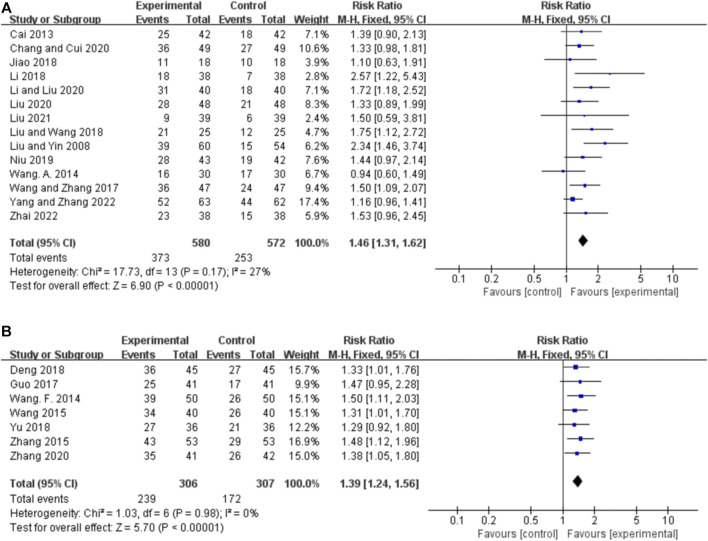
Forest plot of 75% responder rate. **(A)** Orally administered CHM plus CWM vs. CWM. **(B)** Intravenously administered CHM plus CWM vs. CWM.

Fourteen studies compared the 75% responder rate for orally administered CHM plus CWM and CWM, and the fixed-effects model was used because there was low heterogeneity among the studies (*p* = 0.17, *I* ^*2*^ = 27%). We found that orally administered CHM plus CWM was superior to CWM monotherapy regarding 75% responder rate (*RR* 1.46, 95% CI: 1.31 to 1.62, *p* < 0.00001) (Figure 3A), and sensitivity analysis revealed that results were robust against the exclusion of any one study (Supplementary Figure S1A).

##### 3.4.1.2 Intravenously administered CHM

Seven studies compared the 75% responder rate for intravenously administered CHM plus CWM and CWM, and the fixed-effects model was used because there was no heterogeneity among the studies (*p* = 0.98, *I* ^*2*^ = 0%). We found that intravenously administered CHM plus CWM was superior to CWM monotherapy regarding 75% responder rate (*RR* 1.39, 95% CI: 1.24 to 1.56, *p* < 0.00001) (Figure 3B), and sensitivity analysis revealed that results were robust against the exclusion of any one study (Supplementary Figure S1B).

#### 3.4.2 50%–75% responder rate

##### 3.4.2.1 Orally administered CHM

**FIGURE 4:**
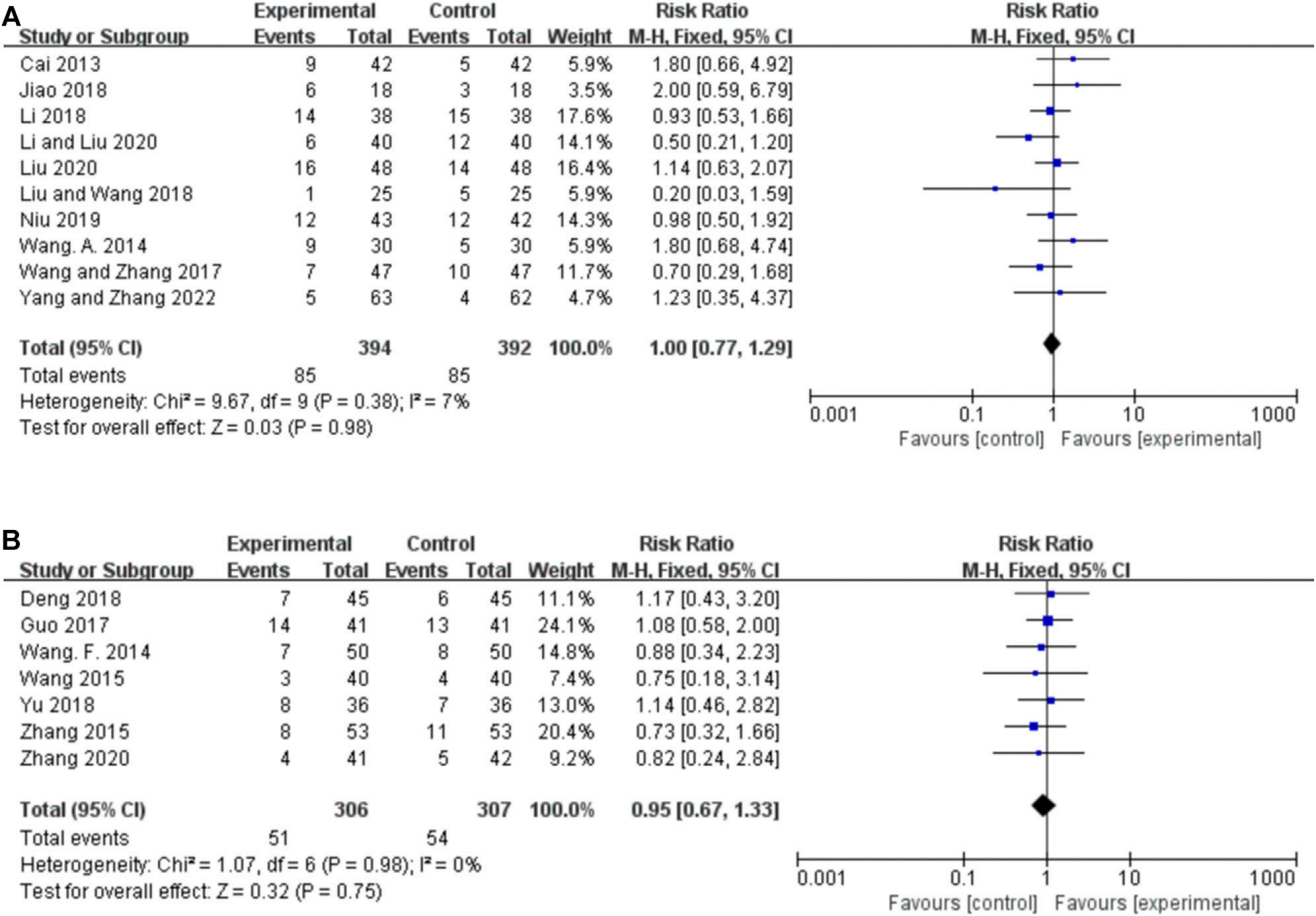
Forest plot of 50%–75% responder rate. **(A)** Orally administered CHM plus CWM vs. CWM. **(B)** Intravenously administered CHM plus CWM vs. CWM.

Ten studies reported the 50%–75% responder rate for orally administered CHM plus CWM and CWM, and the fixed-effects model was used because there was low heterogeneity among the studies (*p* = 0.38, *I* ^*2*^ = 7%). We found that orally administered CHM plus CWM was not superior to CWM monotherapy in increasing 50%–75% responder rate (*RR* 1.00, 95% CI: 0.77 to 1.29, *p* = 0.98) (Figure 4A), and sensitivity analysis revealed that results were robust against the exclusion of any one study (Supplementary Figure S1C).

##### 3.4.2.2 Intravenously administered CHM

Seven studies reported the 50%–75% responder rate for intravenously administered CHM plus CWM and CWM, and the fixed-effects model was used because there was no heterogeneity among the studies (*p* = 0.98, *I* ^*2*^ = 0%). We found that orally administered CHM plus CWM was not superior to CWM monotherapy in increasing 50%–75% responder rate (*RR* 0.95, 95% CI: 0.67 to 1.33, *p* = 0.75) (Figure 4B), and sensitivity analysis revealed that results were robust against the exclusion of any one study (Supplementary Figure S1D).

#### 3.4.3 Subgroup analysis

Subgroup analyses by treatment duration (≤ 3 m, > 3 m) and types of CWM (classical, new, new + classical) showed no statistically differential effects except for the subgroup with only one RCT, and the results of the subgroup analysis were consistent with the overall results (Table 2).

**TABLE 2:** Results of subgroup analysis.

Subgroup		No.	*RR*	95% CI	*I* ^*2*^ (%)	*p-*value for overall effect	*p* interaction
75% responder rate comparing orally administered CHM plus CWM and CWM							
Treatment duration	≤ 3 months	8	1.48	1.28 to 1.70	24	*p* < 0.00001	0.76
	> 3 months	6	1.43	1.22 to 1.68	43	*p* < 0.0001	
Types of CWM	Classical ASMs	8	1.69	1.42 to 2.02	0	*p* < 0.00001	0.01
	New ASMs	6	1.28	1.13 to 1.47	0	0.0002	
75% responder rate comparing intravenously administered CHM plus CWM and CWM							
Treatment duration	≤ 3 months	7	1.39	1.24 to 1.56	0	*p* < 0.00001	-
	> 3 months	0	-	-	-	-	
Types of CWM	Classical ASMs	5	1.40	1.24 to 1.59	0	*p* < 0.00001	0.87
	New ASMs	1	1.47	0.95 to 2.28	-	0.08	
	Classical + New ASMs	1	1.29	0.92 to 1.80	-	0.14	
50%-75% responder rate comparing orally administered CHM plus CWM and CWM							
Treatment duration	≤ 3 months	6	0.88	0.63 to 1.24	15	0.64	0.27
	> 3 months	4	1.19	0.79 to 1.79	0	0.41	
Types of CWM	Classical ASMs	6	0.92	0.65 to 1.29	31	0.62	0.47
	New ASMs	4	1.12	0.74 to 1.69	0	0.59	
50%-75% responder rate comparing intravenously administered CHM plus CWM and CWM							
Treatment duration	≤ 3 months	7	0.95	0.67 to 1.33	0	0.75	-
	> 3 months	0	-	-	-	-	
Types of CWM	Classical ASMs	5	0.86	0.54 to 1.35	0	0.51	0.77
	New ASMs	1	1.08	0.58 to 2.00	-	0.81	
	Classical + New ASMs	1	1.14	0.46 to 2.82	-	0.77	

Note: AEs, adverse events; ASMs, anti-seizure medications; CI, confidence interval; CHM, chinese herbal medicine; CWM, conventional Western medicine; *RR*, risk ratio.

### 3.5 Secondary outcomes

#### 3.5.1 Seizure duration

##### 3.5.1.1 Orally administered CHM

**FIGURE 5:**
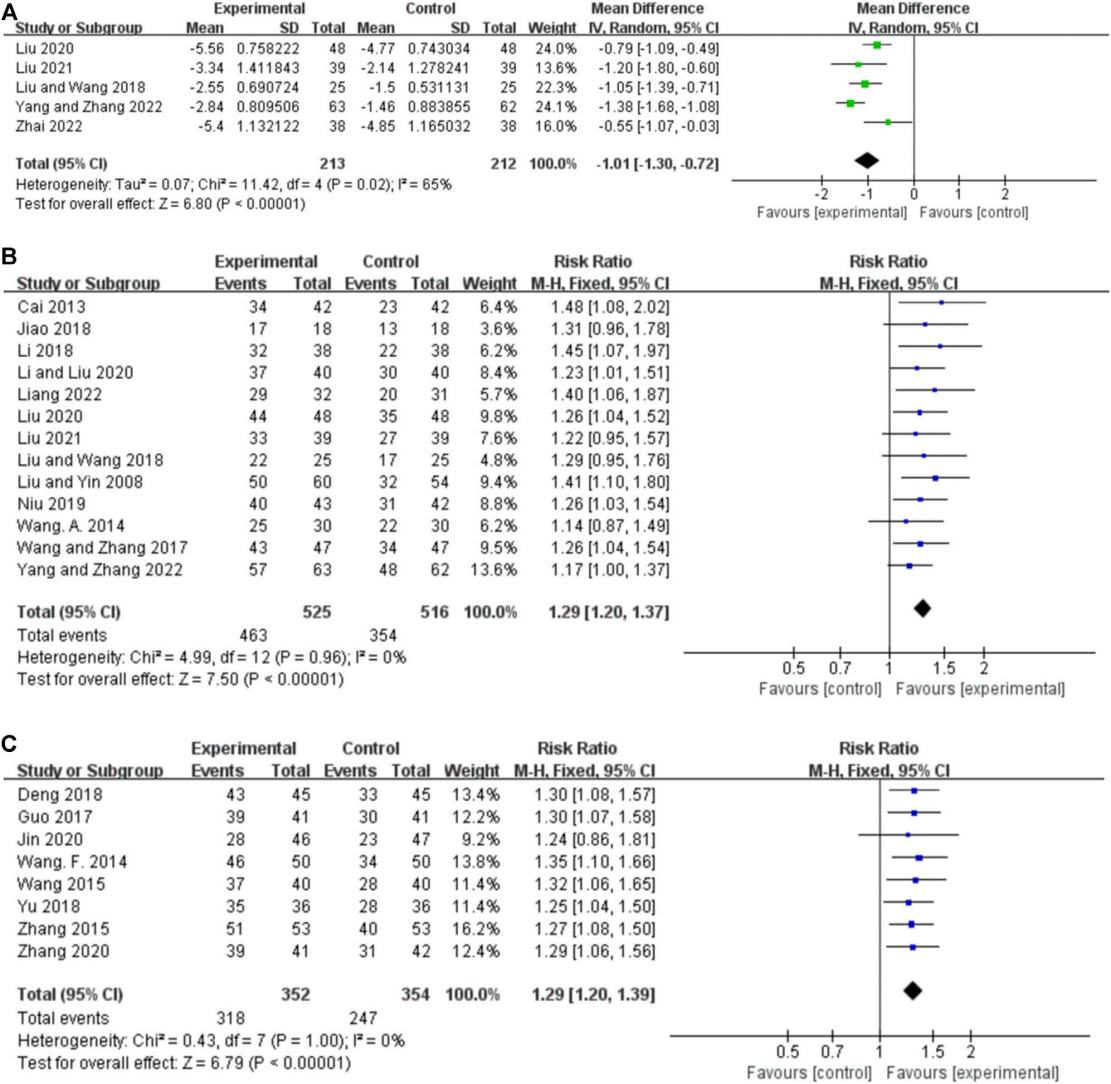
Forest plot. **(A)** Orally administered CHM plus CWM vs. CWM on seizure duration. **(B)** Orally administered CHM plus CWM vs. CWM on total responder rate. **(C)** Intravenously administered CHM plus CWM vs. CWM on total responder rate.

Five studies reported the seizure duration for orally administered CHM plus CWM and CWM, and the random-effects model was used because of the high heterogeneity among the studies (*p* = 0.02, *I* ^*2*^ = 65%). As shown in Figure 5A, orally administered CHM plus CWM reduced seizure duration to a greater extent than did CWM monotherapy (*MD* -1.01, 95% CI: −1.30 to −0.72, *p* < 0.00001). Sensitivity analysis demonstrated that the heterogeneity was significantly decreased (*p* = 0.27, *I* ^*2*^ = 24%) after removing the studies by yang and zhang (2022) (Supplementary Figure S1E).

##### 3.5.1.2 Intravenously administered CHM

One RCT (Jin et al., 2020) reported that salvianolate injection combination therapy was superior to CWM in shorting the seizure duration (*p* < 0.05).

#### 3.5.2 Total responder rate

##### 3.5.2.1 Orally administered CHM

Thirteen studies reported the total responder rate for orally administered CHM plus CWM and CWM, and the fixed-effects model was used because there was no heterogeneity among the studies (*p* = 0.96, *I* ^*2*^ = 0%). We found that orally administered CHM plus CWM was superior to CWM monotherapy in total responder rate (*RR* 1.29, 95% CI: 1.20 to 1.37, *p* < 0.00001) (Figure 5B), and sensitivity analysis revealed that results were robust against the exclusion of any one study (Supplementary Figure S1F).

##### 3.5.2.2 Intravenously administered CHM

Eight studies reported the total responder rate for intravenously administered CHM plus CWM and CWM, and the fixed-effects model was used because there was no heterogeneity among the studies (*p* = 1.00, *I* ^*2*^ = 0%). We found that intravenously administered CHM plus CWM was superior to CWM monotherapy in total responder rate (*RR* 1.29, 95% CI: 1.20 to 1.39, *p* < 0.00001) (Figure 5C), and sensitivity analysis revealed that results were robust against the exclusion of any one study (Supplementary Figure S1G).

#### 3.5.3 Improvement of EEG efficacy

Improvements in EEG efficacy were assessed using EDs and the number of leads involved in ED.

##### 3.5.3.1 Epileptiform discharges

###### 3.5.3.1.1 Orally administered CHM

**FIGURE 6:**
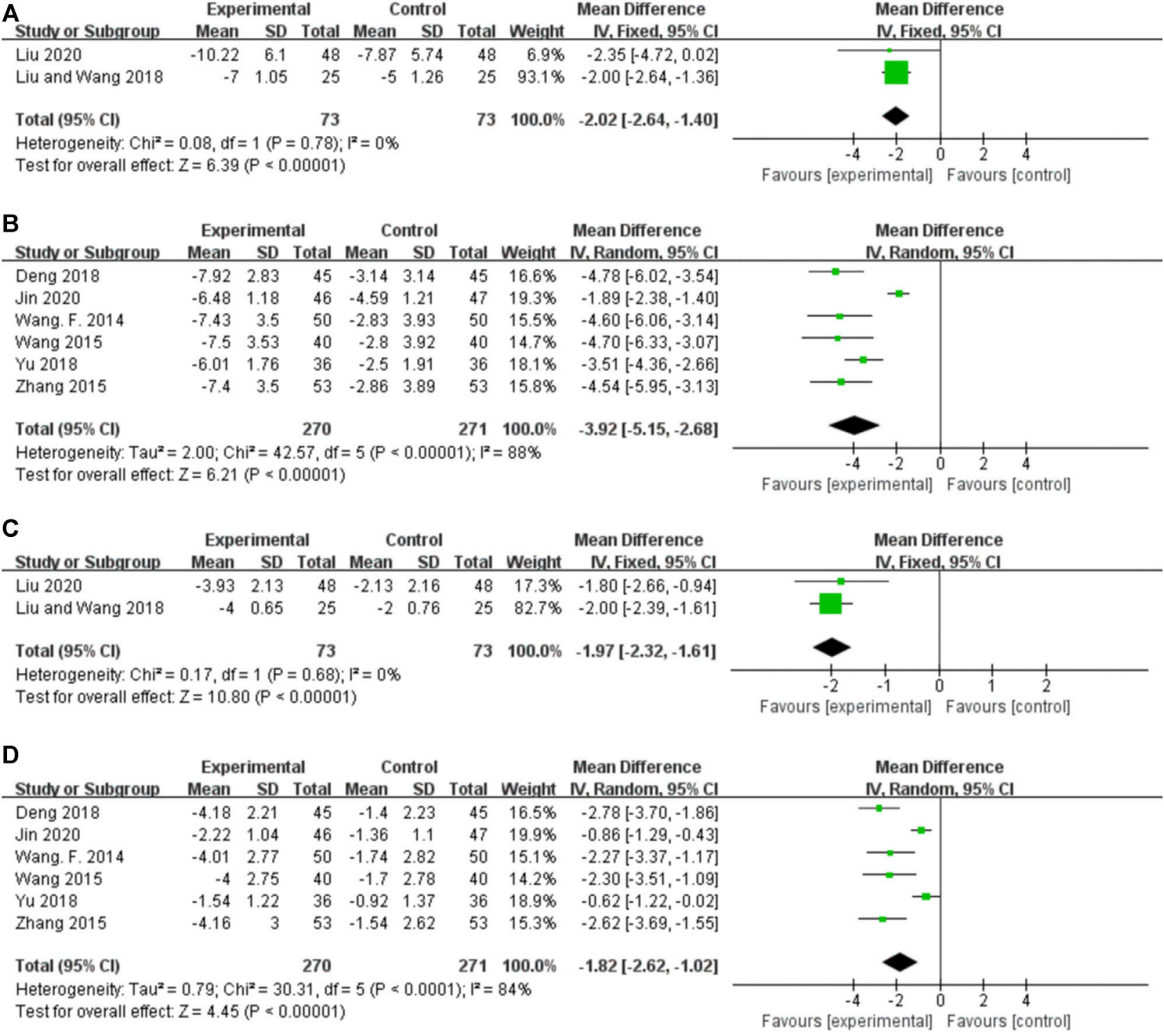
Forest plot. **(A)** Orally administered CHM plus CWM vs. CWM on EDs. **(B)** Intravenously administered CHM plus CWM vs. CWM on EDs. **(C)** Orally administered CHM plus CWM vs. CWM on the number of leads involved in ED. **(D)** Intravenously administered CHM plus CWM vs. CWM on the number of leads involved in ED.

Two studies reported the EDs for orally administered CHM plus CWM and CWM, and the fixed-effects model was used because there was no heterogeneity among the studies (*p* = 0.78, *I* ^*2*^ = 0%). As shown in Figure 6A, orally administered CHM plus CWM resulted in a greater reduction in EDs compared to CWM monotherapy (*MD* -2.02.46, 95% CI: −2.64 to −1.40, *p* < 0.00001).

###### 3.5.3.1.2 Intravenously administered CHM

Six studies reported the EDs for intravenously administered CHM plus CWM and CWM, and the random-effects model was used because of the high heterogeneity among the studies (*p* < 0.00001, *I* ^*2*^ = 88%). As shown in Figure 6B, intravenously administered CHM plus CWM resulted in a greater reduction in EDs compared to CWM monotherapy (*MD* -2.02.46, 95% CI: −2.64 to −1.40, *p* < 0.00001). Sensitivity analysis demonstrated that the heterogeneity was significantly decreased (*p* = 0.37, *I* ^*2*^ = 6%) after removing the studies by Jin et al. (2020) (Supplementary Figure S1H). Different from other studies, the treatment duration of this RCT was less than 3 weeks, which may lead to the clinical heterogeneity.

##### 3.5.3.2 The number of leads involved in epileptiform discharge

###### 3.5.3.2.1 Orally administered CHM

Two studies reported the number of leads involved in ED for orally administered CHM plus CWM and CWM, and the fixed-effects model was used because there was no heterogeneity among the studies (*p* = 0.68, *I* ^*2*^ = 0%). As shown in Figure 6C, orally administered CHM plus CWM resulted in a greater reduction in the number of leads involved in ED than did CWM monotherapy (*MD* -1.97, 95% CI: −2.32 to −1.61, *p* < 0.00001).

###### 3.5.3.2.2 Intravenously administered CHM

Six studies reported the number of leads involved in ED for intravenously administered CHM plus CWM and CWM, and the random-effects model was used because of the high heterogeneity among the studies (*p* < 0.00001, *I* ^*2*^ = 84%). As shown in Figure 6D, intravenously administered CHM plus CWM resulted in a greater reduction in the number of leads involved in ED than did CWM monotherapy (*MD* -1.82, 95% CI: −2.62 to −1.02, *p* < 0.00001), and sensitivity analysis revealed the robustness of the conclusions (Supplementary Figure S1I).

#### 3.5.4 Adverse events

All studies did not report the treatment withdrawal due to AEs, so only the incidence of AEs was analyzed in this research. Only one study (Liu et al., 2020) reported the loss of follow-up but excluded the missed patients from the study, the remaining studies (Liu and Yin, 2008; Cai et al., 2013; Wang, 2014; Wang et al., 2014; Wang et al., 2015; Zhang, 2015; Guo, 2017; Wang and Zhang, 2017; Deng et al., 2018; Jiao, 2018; Li et al., 2018; Liu and Wang, 2018; Yu, 2018; Niu, 2019; Chang and Cui, 2020; Jin et al., 2020; Li and Liu, 2020; Zhang et al., 2020; Liu et al., 2021; Liang et al., 2022; Yang and Zhang, 2022; Zhai et al., 2022) did not report the drug withdrawal or loss of follow-up due to AEs.

Fifteen studies (Liu and Yin, 2008; Cai et al., 2013; Wang, 2014; Wang et al., 2015; Zhang, 2015; Guo, 2017; Wang and Zhang, 2017; Deng et al., 2018; Jiao, 2018; Li et al., 2018; Yu, 2018; Jin et al., 2020; Liu et al., 2020; Zhang et al., 2020; Liang et al., 2022) reported the incidence of AEs. Among these studies, one study (Deng et al., 2018) did not provide the details, and one study (Li et al., 2018) reported that no AEs occurred in either the experimental group or the control group. The major AEs were gastrointestinal reactions, dizziness, sleep disorders, and headache.

##### 3.5.4.1 Orally administered CHM

**FIGURE 7:**
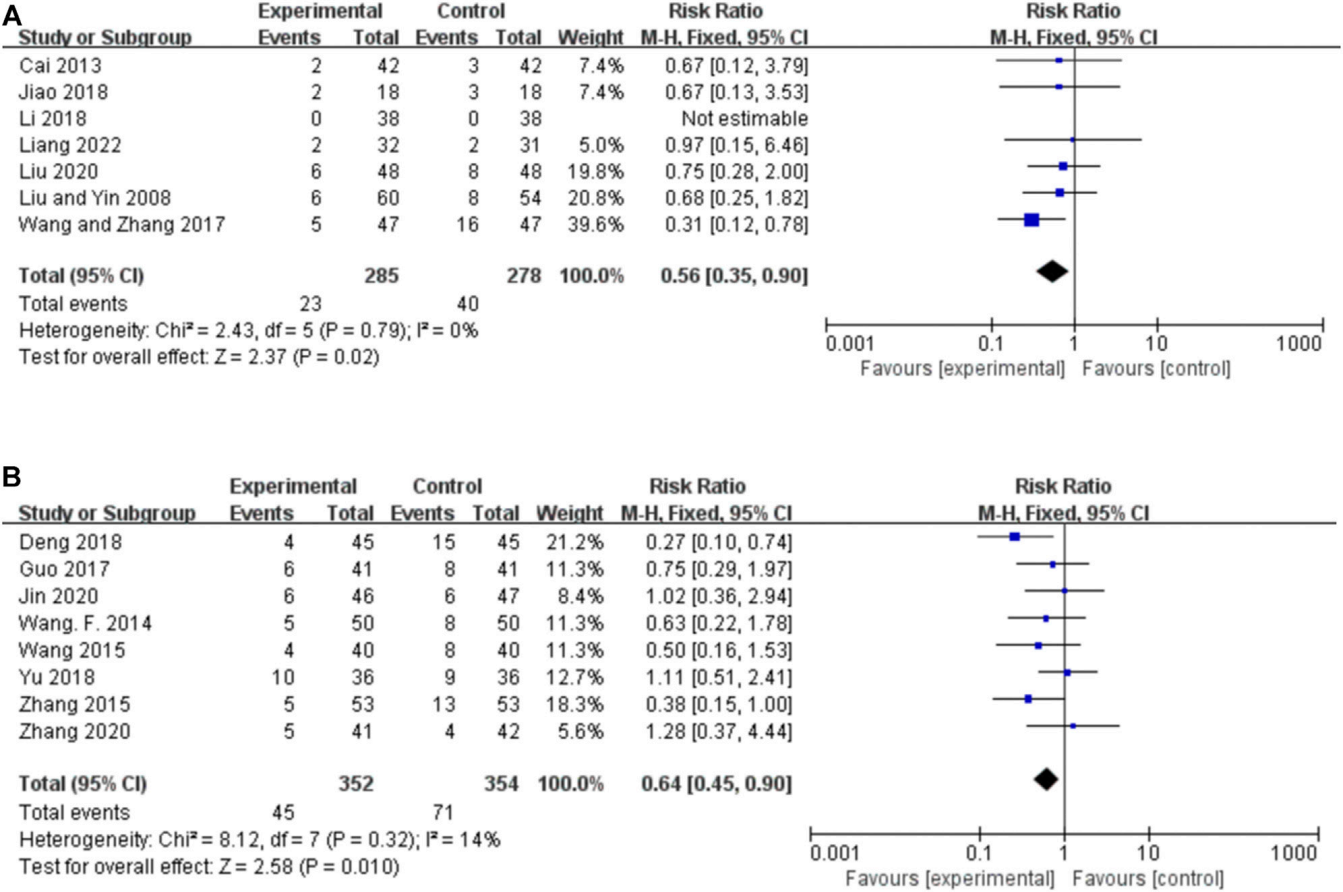
Forest plot of the incidence of AEs. **(A)** Orally administered CHM plus CWM vs. CWM. **(B)** Intravenously administered CHM plus CWM vs. CWM.

Seven studies reported the AEs for orally administered CHM plus CWM and CWM, and the fixed-effects model was used because there was no heterogeneity among the studies (*p* = 0.79, *I* ^*2*^ = 0%). As shown in Figure 7A, orally administered CHM plus CWM resulted in a greater reduction in the incidence of AEs compared to CWM (*RR* 0.56, 95% CI: 0.35 to 0.90, *p* = 0.02), and sensitivity analysis revealed the robustness of the conclusions (Supplementary Figure S1J).

##### 3.5.4.2 Intravenously administered CHM

Eight studies reported the AEs for intravenously administered CHM plus CWM and CWM, and the fixed-effects model was used because there was low heterogeneity among the studies (*p* = 0.32, *I* ^*2*^ = 14%). As shown in Figure 7B, intravenously administered CHM resulted in a greater reduction in the incidence of AEs compared to CWM (*RR* 0.64, 95% CI: 0.45 to 0.90, *p* = 0.010), and sensitivity analysis revealed the robustness of the conclusions (Supplementary Figure S1K).

#### 3.5.5 Risk of publication bias

**FIGURE 8:**
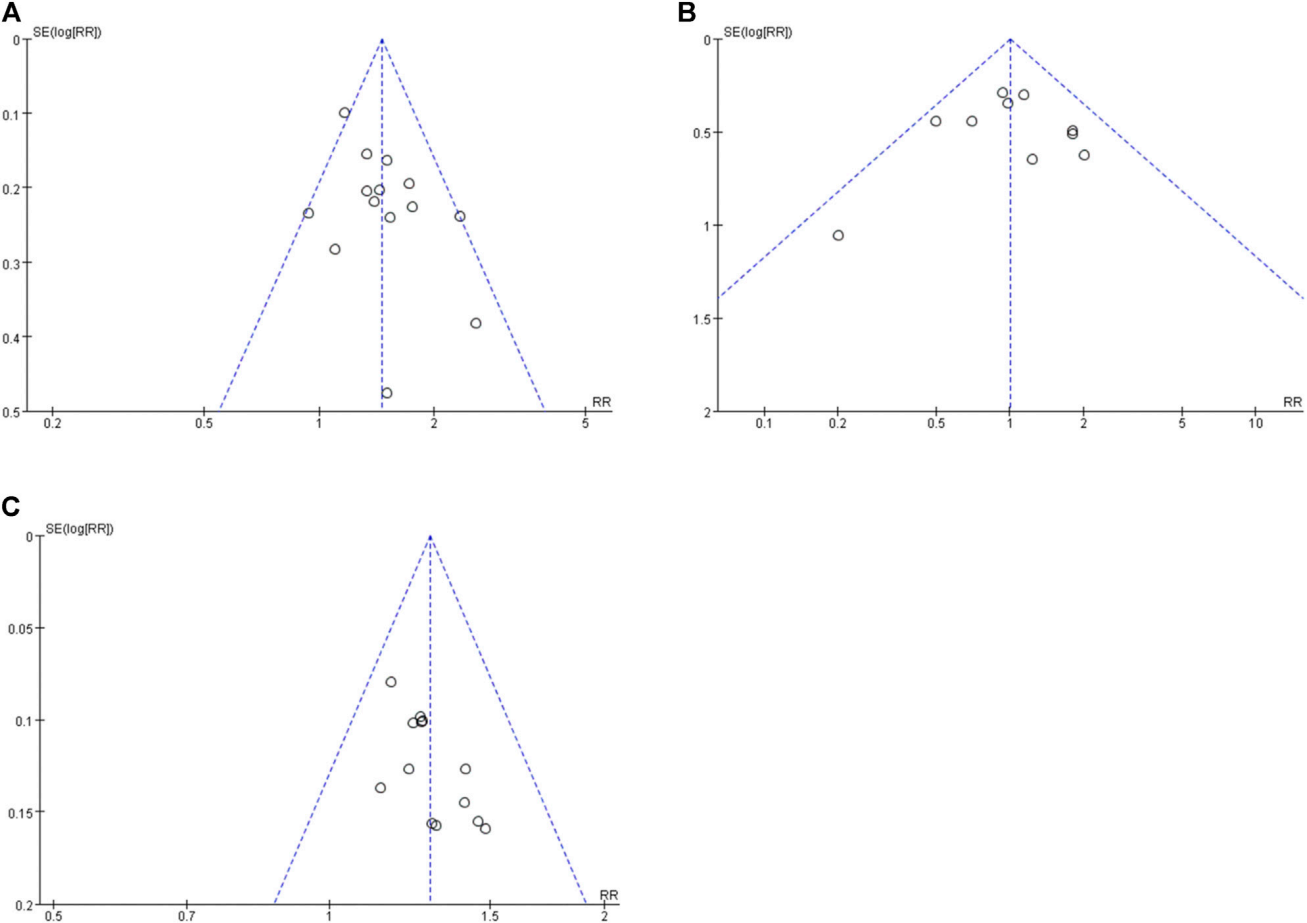
Funnel plots. **(A)** Orally administered CHM plus CWM vs. CWM on 75% responder rate. **(B)** Orally administered CHM plus CWM vs. CWM on 50%-75% responder rate. **(C)** Orally administered CHM plus CWM vs. CWM on total responder rate.

The funnel plots of orally administered CHM plus CWM vs. CWM on 75% responder rate, 50%–75% responder rate, and total responder rate were asymmetric visually, suggesting publication bias probably existed (Figures 8A–C). Thus, Egger’s test was performed to evaluate the effect of publication bias on the meta-analysis results, and Egger’s test of the 75% responder rate and 50%–75% responder rate suggested no significant publication bias was detected (Egger’s test *p* = 0.080 and *p* = 0.835, respectively). However, Egger’s test of the total responder rate indicated potential publication bias (Egger’s test *p* = 0.014) (Supplementary Figure S2A–C). Therefore, we conducted trim-and-fill test analysis, and the result indicated that this publication bias did not affect the conclusion (Supplementary Table S3).

#### 3.5.6 Certainty assessment

The certainty of evidence for the meta-analysis was evaluated by GRADE (Table 3). The quality of evidence ranged from low to high. The primary reasons for downgrading were inconsistency (high heterogeneity), imprecision (small sample size), and other considerations (publication bias).

**TABLE 3:** GRADE summary of outcomes.

NO.	Study design	Certainty assessment					Summary of results						Importance
		Risk of bias	Inconsistency	Indirectness	Imprecision	Other considerations	No. of patients		Effect (95% CI)		Certainty		
							T	C	Relative	Absolute			
Orally administered CHM plus CWM vs. CWM on 75% responder rate													
14	RCT	Not serious^a^	Not serious	Not serious	Not serious	Not serious^g^	580	572	*RR* 1.46 (1.31–1.62)	-	⊕⊕	High	Critical
											⊕⊕		
Intravenously administered CHM plus CWM vs. CWM on 75% responder rate													
7	RCT	Not serious^a^	Not serious	Not serious	Not serious	Not serious^g^	306	307	*RR* 1.39 (1.24–1.56)	-	⊕⊕	High	Critical
											⊕⊕		
Orally administered CHM plus CWM vs. CWM on 50%-75% responder rate													
10	RCT	Not serious^a^	Not serious	Not serious	Serious^d^	Not serious^g^	394	392	*RR* 1.00 (0.77–1.29)	-	⊕⊕	Moderate	Critical
											⊕ ○		
Intravenously administered CHM plus CWM vs. CWM on 50%-75% responder rate													
7	RCT	Not serious^a^	Not serious	Not serious	Serious^d^	Not serious^g^	306	307	*RR* 0.95 (0.67–1.33)	-	⊕⊕	Moderate	Critical
											⊕ ○		
Orally administered CHM plus CWM vs. CWM on seizure duration													
5	RCT	Not serious^a^	Serious^b^	Not serious	Not serious	Not serious^g^	213	212	-	*MD* 1.01 lower (1.30 lower to 0.72 lower)	⊕⊕	Moderate	Important
											⊕ ○		
Orally administered CHM plus CWM vs. CWM on total responder rate													
13	RCT	Not serious^a^	Not serious	Not serious	Not serious	Serious^f^	525	516	*RR* 1.29 (1.20–1.37)	-	⊕⊕	Moderate	Important
											⊕ ○		
Intravenously administered CHM plus CWM vs. CWM on total responder rate													
8	RCT	Not serious^a^	Not serious	Not serious	Not serious	Not serious^g^	352	354	*RR* 1.29 (1.20–1.39)		⊕⊕	High	Important
											⊕⊕		
Orally administered CHM plus CWM vs. CWM on EDs													
2	RCT	Not serious^a^	Not serious	Not serious	Serious^e^	Not serious^g^	73	73	-	*MD* 2.02 lower (2.64 lower to 1.40 lower)	⊕⊕	Moderate	Important
											⊕ ○		
Intravenously administered CHM plus CWM vs. CWM on EDs													
6	RCT	Not serious^a^	Very serious^c^	Not serious	Not serious	Not serious^g^	270	271	-	*MD* 3.92 lower (5.15 lower to 2.68 lower)	⊕⊕	High	Important
											⊕⊕		
Orally administered CHM plus CWM vs. CWM on the number of leads involved in ED													
2	RCT	Not serious^a^	Not serious	Not serious	Serious^e^	Not serious^g^	73	73	-	*MD* 1.97 lower (2.32 lower to 1.61 lower)	⊕⊕	Moderate	Important
											⊕ ○		
Intravenously administered CHM plus CWM vs. CWM on the number of leads involved in ED													
6	RCT	Not serious^a^	Very serious^c^	Not serious	Not serious	Not serious^g^	270	271	-	*MD* 1.82 lower (2.62 lower to 1.02 lower)	⊕⊕	Low	Important
											○ ○		
Orally administered CHM plus CWM vs. CWM on adverse events													
7	RCT	Not serious^a^	Not serious	Not serious	Not serious	Not serious^g^	285	278	*RR* 0.56 (0.35–0.90)	-	⊕⊕	High	Important
											⊕⊕		
Intravenously administered CHM plus CWM vs. CWM on adverse events													
8	RCT	Not serious^a^	Not serious	Not serious	Not serious	Not serious^g^	352	354	*RR* 0.64 (0.45–0.90)	-	⊕⊕	High	Important
											⊕⊕		

Notes: C, control group; CI, confidence interval; RCT, randomized controlled trial; *RR*, risk ratio; *MD*, mean difference; T, treatment group.

^a^ Most of the included studies were at unclear or low risk of bias.

^b^ 50% < *I* ^2^ < 75% for heterogeneity.

^c^ *I* ^2^ ≥ 75% for heterogeneity.

^d^ 95% CI, contains 1.

^e^ Small sample size.

^f^ Publication bias.

^g^ No test for publication bias.

## 4 Discussion

### 4.1 Summary of main findings

In this study, a total of 23 RCTs involving 1,901 (954/947) patients were included to systematically evaluate the efficacy and safety of CHM add-on treatment for PSE. We found that CHM plus CWM therapy offers significant benefits over CWM monotherapy on metrics of 75% responder rate, seizure duration, and total responder rate. The results of the EEG efficacy proved that CHM plus CWM significantly decreased EDs and the number of leads involved in ED. In terms of safety, based on available evidence, we can conservatively assume that CHM add-on treatment does not cause serious AEs and increases the occurrence of AEs. However, the level of evidence was low to high due to the potential publication bias, heterogeneity, and small sample size.

However, CHM add-on treatment was not superior to CWM monotherapy in terms of 50%–75% responder rate, although more patients were effective in the CHM group compared to the CWM group in the majority of our included studies. It has been suggested (Sondhi et al., 2020) that the efficacy in terms of seizures might be related to differences in neurological disorders as a result of comparison between interventions in patients with epilepsy. And the efficacy is time-dependent. There are also data from a study (Yang et al., 2022) showing that short-term treatment of epilepsy is more effective than long-term treatment. This might all contribute to the lack of significant difference in the 50%–75% responder rate. Therefore, whether CHM combination therapy has a significant therapeutic effect on 50%–75% response rate still needs to be further explored and validated by high-quality RCTs.

### 4.2 Secondary findings

We evaluated the effects of treatment duration and types of CWM on 75% responder rate and 50%–75% responder rate, which are relevant to clinical practice. Notably, the results from subgroup analyses were not fully consistent. We found that CHM combination therapy significantly improved efficacy regardless of the treatment durations (≤3 months or >3 months). However, for the 75% responder rate, we found a positive effect with classical ASMs, but not with new ASMs and classical ASMs + new ASMs. We surmised that the differences in efficacy across types of CWM could be attributed to the number of included studies (only 1 study used classical new ASMs or ASMs + new ASMs, respectively).

Sensitivity analysis demonstrated the robustness of our findings. However, the results of the analyses of some outcome indicators were remarkably heterogeneous, which may be due to the differences in the type of disease or disease staging of the patients, suggesting that reporting of clinical characteristics of patients should be refined in studies on co-morbidities and that these findings should also be treated with caution. Furthermore, more studies are needed to determine whether CHM add-on treatment can improve seizure duration and EEG efficacy (EDs and the number of leads involved in ED).

Additionally, we identified publication bias in the total responder rate. Notably, the trim-and-fill analysis found that several RCTs showing negative findings remained unpublished. Therefore, we reduced the certainty of the evidence accordingly.

### 4.3 Implications for research

Numerous *in vivo* and *in vitro* studies (Ho et al., 2014; Jia et al., 2018; Hu et al., 2019; Jin et al., 2023) have shown that CHM can exert neuroprotective effects by regulating central neurotransmitters (Gao et al., 2016; Jin et al., 2019; Deng et al., 2020), inhibiting apoptosis (Yang et al., 2020), regulating oxidative stress, and inhibiting inflammatory response (Xia et al., 2021), thereby improve electroencephalographic activity, and effectively controlling seizure rates. At the same time, it also can alleviate AEs caused by CWM and improve patients’ quality of life. We summarized commonly used botanical drugs and prescription (Supplementary Table S4), providing scientific guidance for clinical practice and for the design and implementation of clinical studies. We refer to the ConPhyMP statement to ensure the rigor of conclusions (Heinrich et al., 2022). The top three botanical drugs for PSE were *Poria cocos* (Schw.) Wolf [Poria], *Pineilia ternata* (Thunb.) Makino [Araceae; Pinelliae rhizoma] and *Conioselinum anthriscoides* ‘Chuanxiong’ [Apiaceae; Chuanxiong rhizoma], and the most used prescriptions was Chaihu-Longgu-Muli Decoction, suggesting that they could be potentially effective prescriptions for PSE.

With regard to individual drugs, Gao et al. (2016) found that the total triterpenes of *Poria cocos* (Schw.) Wolf [Poria] could inhibit maximal electroshock- and pentylenetetrazol-induced seizures with significant antiepileptic effects, which might be achieved by increasing gamma-aminobutyric acid (GABA) content, decreasing glutamate content, and having good sedative effects. Deng et al. (2020) found that the pinellia total alkaloids might reduce the incidence and frequency of spontaneous recurrent seizures in the pilocarpine-induced epilepsy rat model, and might modulate the antiepileptic effects of the GABAergic system by increasing the levels of GABA and glutamate decarboxylase 65, and suppressed the levels of GABA transporter-1 and GABA transaminase, as well as regulating the expression levels of GABAAR α5, δ, α4 and γ2 subunits in hippocampal formation. In addition, Jin et al. (2019) found that the antiepileptic effect of Tetramethylpyrazine, a major chemical constituent alkaloid of *Conioselinum anthriscoides* ‘Chuanxiong’ [Apiaceae; Chuanxiong rhizoma], unlike conventional ASMs that act on sodium channels, might be through its inhibition of excitatory synaptic transmission by its inhibitory effect on calcium channels, and inhibit the development of seizures.

Regarding the overarching polyherbal formula, in recent years, there have been many studies on the antiepileptic effects of Chaihu-Longgu-Muli decoction (CLMD) that have progressed. CLMD could significantly inhibited autophagy in the hippocampal dentate gyrus of lithium-pilocarpine-induced epilepsy rat model and attenuated seizure frequency, especially at high doses, and the mechanism might be related to the upregulation of mTOR expression and downregulation of Beclin-1 and LC3B expression (Yang et al., 2020). Furthermore, CLMD significantly inhibited the frequency and duration of seizures in the lithium chloride-pilocarpine temporal lobe epilepsy rat model and decreased the expression of NLRP3, Caspase-1, TNF-α and IL-1β in hippocampal neurons of temporal lobe epilepsy rats, indicating that CLMD could inhibit the onset of pyroptosis (Xia et al., 2021).

The improvement of these pathophysiological mechanisms can significantly inhibit aberrant neuronal discharges, thus reducing seizure frequency and duration (Prince, 1985). Modern medical studies have shown that the pathogenesis of epilepsy may be closely related to Neuroimmune inflammation (Andersen and Qintana, 2022; Vezzani et al., 2022; Yue et al., 2022). Although studies on the antiepileptic effects of CHM have made many advances in improving neuroinflammation, no high-quality studies have appeared in neuroimmunity, and future studies could go further in this direction.

### 4.4 Implications for practice

Our meta-analysis suggests that CHM combination therapy has great potential for the treatment of PSE and deserves further exploration. First, we found that CHM combination therapy could enhance the clinical efficacy regardless of the treatment duration (≤3 months, >3 months) and types of CWM (classical ASMs, new ASMs), but given the limited availability of high-quality evidence, more research is needed to verify the reliability and scientific validity of this finding. Second, the quality of future studies would be improved by standardizing study protocols, unifying diagnostic criteria, and improving the implementation of randomization, allocation concealment, and blinding. Third, more scientific and objective outcome indicators like 6- or 12-month seizure freedom should be selected, and follow-up times should be extended. Fourth, the extraction and processing of Chinese medicinal preparations urgently need to be standardized to enhance the comparability of studies and the stability of conclusions. Finally, clinical studies should strictly follow the CONSORT 2010 statement (Schulz et al., 2022) and CONSORT Extension for Chinese Herbal Medicine Formulas 2017 (Cheng et al., 2017) to improve reporting quality. High-quality, large-sample, multi-center, double-blind RCT studies will provide more reliable guidance for clinical practice, which is conducive to comprehensively evaluating the efficacy and safety of CHM combination therapy and identifying highly effective and rational treatment protocols.

### 4.5 Strengths

Our study has several strengths. First, we have adopted updated and internationally recognized epilepsy-related indicators such as 75% responder rate and seizure duration to evaluate the effects of CHM combination therapy, further supporting the use of CHM in treating PSE. Second, we improved the credibility of the results by exploring the sources of heterogeneity, the robustness of the results, and the impact of some characteristics on the efficacy of CHM combination therapies with sensitivity and subgroup analyses. Third, the GRADE was used to assess the evidence quality. Finally, to minimize heterogeneity in the included studies, we analyzed orally administered CHM and intravenously administered CHM separately. In addition, we limited the types of interventions and excluded co-treatment with other traditional Chinese medicine treatments (e.g., acupuncture and moxibustion).

### 4.6 Limitations

Some limitations in this work should be noted. First, there are no standardized diagnostic criteria for PSE, and the different ILAE diagnostic criteria (e.g., ILAE, 1981 vs. ILAE, 2017) may affect the assessment of CHM combination therapy efficacy. Second, all included RCTs were conducted in China, and the conclusions of our study should be validated in patients of other races. Third, the strength of our conclusions may be limited by poor methodological quality (e.g., some of the included studies did not describe the allocation concealment and blinding in detail), small sample sizes, and the potential risk of bias. Fourth, the extraction and processing of botanical drugs are not standardized, which is not conducive to quality control and chemical analysis and may lead to heterogeneity. Fifth, differences in administrations of CHM in RCTs (including decoction, intravenous injection, tablet, capsule, pill, etc.), as well as in interventions in the control group, may lead to a risk of bias that reduces the credibility of the findings, so we should be cautious about the conclusions. Furthermore, subgroup analyses could not be performed for other study characteristics that could lead to heterogeneity (e.g., diagnostic criteria, subject characteristics, drug composition, and whether stroke-related surgical treatment was performed) due to insufficient reported data for the inclusion of RCTs, which introduces additional uncertainty into the comparisons. Finally, we were unable to compare the long-term effects of CHM combination therapy because of the limited number of studies and follow-up data.

## 5 Conclusion

This systemic review and meta-analysis suggests that CHM adjunctive therapy for PSE is superior to CWM monotherapy in terms of efficacy and safety, indicating that CHM adjunctive therapy is a potential therapy for PSE. However, the effects of CHM adjunctive therapy on 50%–75% responder rate remain to be further investigated. In addition, because the evidence quality in this study is unstable, more well-designed long-term follow-up RCTs are needed to evaluate CHM adjunctive therapy efficacy and safety.

## Data Availability

The original contributions presented in the study are included in the article/Supplementary Material, further inquiries can be directed to the corresponding author.
