# Supplementary material for: Efficacy and safety of Chinese herbal medicine in post-stroke epilepsy: a systematic review and meta-analysis
Source: Front Pharmacol. 2023 Nov 21;14:1286093. doi: 10.3389/fphar.2023.1286093 (PMC10703176; doi:10.3389/fphar.2023.1286093)
Supplement: Supplementary file 1 [file Table1.docx]

Supplementary Material

**Table of contents**

[1 Supplementary Tables 2](#_Toc3421)

[Supplementary Table S1. Reasons for exclusion of studies after full text review 2](#_Toc31272)

[Supplementary Table S2. The detailed information of the CHMs prescribed 7](#_Toc2737)

[Supplementary Table S3. Trim-and-fill test of the total responder rate in comparison of orally administered CHM plus CWM vs. CWM. 13](#_Toc15112)

[Supplementary Table S4. Frequency ranking of Chinese herbal medicine. 14](#_Toc4124)

[2 Supplementary Figures 16](#_Toc31429)

[Supplementary Figure S1. Sensitivity analysis 16](#_Toc26765)

[Supplementary Figure S2. Egger’s plot 19](#_Toc5019)

[3 Supplementary File 20](#_Toc31793)

[Supplementary File S1. Search strategy 20](#_Toc28765)

[Supplementary File S2. The PRISMA checklist of this meta-analysis 38](#_Toc2177)

# Supplementary Tables

## Supplementary Table S1. Reasons for exclusion of studies after full text review

| Exclusion reasons （n） | Study ID | Title of excluded studies |
| --- | --- | --- |
| The population did not meet the inclusion criteria (n = 40) | Xing 2022 | Efficacy and safety of salvia miltiorrhiza polyphenolate combined with lamotrigine in the treatment of stroke-induced epilepsy in older adults |
|  | Chen 2021a | Clinical observation of Kangxian Jiejing Decoction as an adjunctive treatment for post-stroke refractory epilepsy with wind-phlegm upheaval |
|  | Wang 2021 | Investigation of the effect of Tianma Gouteng Decoction combined with sodium valproate in the treatment of secondary epilepsy after stroke |
|  | Liu 2021 | Analysis of the efficacy of Huoxue Dingxian Formula in the discriminatory treatment of secondary epilepsy after cerebral infarction |
|  | Chen 2021b | Efficacy of Tianma Gouteng Decoction combined with sodium valproate in the treatment of secondary epilepsy after stroke |
|  | Deng 2020 | Clinical observation on the treatment of secondary epilepsy after cerebral infarction with asparagine injection combined with oxcarbazepine |
|  | Qiu 2020 | Effectiveness of Dingxian Pill combined with Tongqiao Huoxue Decoction as an adjunct to Western medicine in the treatment of post-stroke epilepsy |
|  | Zhang 2020 | A study on the effect of Ditan Decoction combined with topiramate tablets in the treatment of cerebral infarction secondary to epilepsy |
|  | Xue 2020 | Clinical application of Chaihu Longgu Muli Decoction for post-stroke epilepsy |
|  | Li 2020 | Clinical observation of 36 cases of late-onset epilepsy after cerebral infarction treated with combined Chinese and Western medicine |
|  | Yu 2019 | Clinical effects of Tianhu Kangxian Tablets in the treatment of post-stroke epilepsy with wind and phlegm blockage |
|  | Fan 2019 | Efficacy of combined Chinese and Western medicine in the treatment of post-stroke epilepsy |
|  | Xia 2019 | Investigation of the effect of using Xingnaojing Injection and carbamazepine in the treatment of secondary epilepsy after stroke |
|  | Xu 2019 | Efficacy of Salvia Miltiorrhiza Polyphenolate Injection Combined with Sodium Valproate in the Treatment of Post-stroke Epilepsy |
|  | Chen 2019 | Efficacy of self-prepared Tongnao Dingxian Pills in the treatment of epilepsy secondary to cerebrovascular disease |
|  | Li 2018a | Clinical effect of Huoxue Dingxain Formula in the treatment of secondary epilepsy after cerebral infarction |
|  | Ouyang 2018 | Clinical treatment effect of Dianxianning Tablets applied to epilepsy secondary to stroke |
|  | He 2018 | Study on the application value of combined Chinese and Western medicine in the treatment of late-onset epilepsy after cerebral infarction |
|  | Wang 2018 | Clinical efficacy of Xingnaojing Injection adjunctive therapy with carbamazepine in the treatment of secondary epilepsy after stroke |
|  | Miao 2018 | A clinical study to investigate stroke secondary to epilepsy |
|  | Guo 2018 | Efficacy of Xingnaojing Injection combined with carbamazepine in the treatment of 32 patients with secondary epilepsy after stroke and its effect on their quality of life |
|  | Li 2018b | Study on the clinical efficacy and safety of Xingnaojing Injection with carbamazepine in the treatment of secondary epilepsy after stroke |
|  | Xu 2017 | Analysis of clinical effects of combined Chinese and Western medicine in the treatment of epilepsy |
|  | Liu 2017 | Xingnaojing combined with Lamotrigine and Sodium Valproate in treatment of elderly secondary epilepsy due to stroke |
|  | Jiang 2016 | Evaluation of clinical efficacy of combined Chinese and Western medicine in the treatment of late-onset epilepsy after cerebral infarction |
|  | Li 2016 | Combination of Chinese and Western medicine in the treatment of late-onset epilepsy after cerebral infarction |
|  | Zhang 2016 | Clinical effects of combined Chinese and Western medicine in the treatment of post-stroke secondary epilepsy |
|  | Zhou 2016 | Clinical effect of carbamazepine and Xingnaojing combined treatment in patients with secondary epilepsy after stroke |
|  | Zhao 2016 | Clinical effect analysis of Xingnaojing adjuvant to carbamazepine in the treatment of secondary epilepsy after stroke |
|  | Wang 2016 | Clinical effect of Xingnaojing adjuvant to carbamazepine in the treatment of secondary epilepsy after stroke |
|  | Yu 2016 | Clinical efficacy and safety evaluation of Xingnaojing adjuvant to carbamazepine in the treatment of secondary epilepsy after stroke |
|  | Zhou 2015 | Clinical study on the identification and treatment of secondary epilepsy after cerebral infarction with Huoxue Dingxian Formula |
|  | Shang 2015 | Clinical efficacy of combined Chinese and Western medicine in the treatment of post-stroke secondary epilepsy |
|  | Pan 2015 | Tongqiao Huoxue Decoction combined with Western medicine for post-stroke epilepsy in 48 cases |
|  | Wei 2015 | Evaluation of the efficacy of Xingnaojing as an adjunct to carbamazepine in the treatment of post-stroke secondary epilepsy |
|  | Zhang 2014 | Efficacy of Xingnaojing combined with carbamazepine in the treatment of secondary epilepsy after stroke |
|  | Chen 2014 | An investigation into the clinical efficacy of Xingnaojing as an adjunct to carbamazepine in the treatment of post-stroke secondary epilepsy |
|  | Yin 2012 | Clinical study on the treatment of late-onset wind-phlegm occlusion type post-stroke epilepsy with Xifeng Dingxian Decoction |
|  | Wu 2012 | Clinical observation of combined Chinese and Western medicine in the treatment of epilepsy caused by cerebrovascular disease |
|  | Liu 2011 | Clinical study on the treatment of epilepsy after cerebral infarction with Huoxue Anxian Pills |
| The outcome did not meet the inclusion criteria (n = 13) | Wu 2023 | Observation on the efficacy of self-proposed Huatan-Quyu-Tongqiao formula in treating post-stroke epilepsy and its effect on cognitive function and serum Hcy and CRP |
|  | Li 2023 | Effect of added Chaihu Shugan Decoction combined with sodium valproate on neuron -specific enolase level in patients with poststroke epilepsy |
|  | Fang 2023 | Clinical observation on comprehensive treatment of post-stroke epilepsy with traditional Chinese medicine |
|  | Chen 2021 | Efficacy of self-prepared Tongluo Huatan Formula combined with oxcarbazepine in the treatment of post-ischemic stroke epilepsy (phlegm-stasis type) and the effect on seizures and serum indexes |
|  | Guo 2021 | Effect of Chaihu Longgu Muli Decoction as an adjunctive treatment for post-stroke epilepsy on electroencephalographic indices and seizures |
|  | Li 2020 | Combination of sodium valproate and Chinese medicine in the treatment of late-onset epilepsy after cerebral infarction |
|  | Jiang 2020 | Attenuating the Post-Stroke Epilepsy Using the Si-Miao-Yong-An Decoction for Treatment |
|  | Sun 2018 | Clinical efficacy of Wuchong Decoction combined with clonidine in the treatment of post-stroke secondary epilepsy |
|  | Jiang 2016 | Clinical observation of 35 cases of post-stroke epilepsy treated with Si-Miao-Yong-An Formula |
|  | Liu 2014 | Effectiveness of combined Chinese and Western medicine treatment for late-onset epilepsy after cerebral infarction |
|  | Yang 2013 | Efficacy of Danxing Ningxian Granules in the treatment of epilepsy secondary to cerebral infarction |
|  | Luo 2012 | Clinical study on the treatment of secondary epilepsy after cerebral infarction with Huoxue Dingxian Formula |
|  | Wang 2003 | Efficacy of Kangxian Decoction in post-cerebrovascular disease epilepsy |
| The design did not meet the inclusion criteria (n = 13) | Guo 2023 | Based on the theory of "long illness entering the collaterals", this paper discusses the effect of Tongqiao Huoxue Decoction on the efficacy and cerebral hemodynamics of patients with secondary epilepsy after ischemic stroke |
|  | Zhang 2022 | Effect of Yinao Anshen Decoction Combined with levetiracetam on epilepsy secondary to subarachnoid hemorrhage and its infIuence on EEG activity |
|  | Shang 2022 | Clinical observation on Chaihu-Shugan decoction combined with sodium valproate in the treatment of qi stagnation and blood stasis type post ischemic stroke epilepsy |
|  | Gong 2019 | Clinical observation of 40 cases of post-stroke late-onset epilepsy treated with Xifeng Dingxian Decoction |
|  | Luo 2016 | Clinical study of 40 cases of post-stroke late-onset epilepsy (wind-phlegm blockage type) treated with Xifeng Dingxian Decoction |
|  | Wang 2015 | Clinical treatment experience of Dianxianning Tablets applied to epilepsy secondary to stroke |
|  | Liu 2013 | Clinical study on the treatment of late-onset epilepsy after cerebral infarction by combining Chinese and Western medicine |
|  | Ying 2013 | Experience of Chinese medicine treatment for epilepsy secondary to cerebral infarction |
|  | Tang 2009 | Clinical observation on the treatment of late-onset epilepsy after stroke with Chaihu Longgu Muli Decoction |
|  | Xu 2007 | 33 cases of post-stroke epilepsy treated with Chaihu Longgu Muli Decoction |
|  | Chen 2007 | Clinical report on the treatment of late-onset post-stroke epilepsy with the Huayu Dingxian Formula |
|  | Cao 2006 | 61 cases of late onset epilepsy after cerebral infarction treated with Ningxian San |
|  | Zhao 2001 | Clinical observation of status epilepticus after cerebral infarction treated with combination of Chinese and Western medicine |
| The intervention did not meet the inclusion criteria (n = 8) | Wei 2022 | The use of Qingxin Wendan Decoction combined with prophylactic intervention in secondary epilepsy after stroke |
|  | Hong 2018 | Efficacy of Chinese medicine in the comprehensive treatment of post-stroke epilepsy in the elderly |
|  | Cao 2018 | Clinical efficacy of combined Chinese and Western medicine in the treatment of epilepsy secondary to stroke |
|  | Chen 2016 | Clinical efficacy, improvement in cognitive function and safety of sodium valproate combined with Pingxian Granules versus carbamazepine combined with butylphthalide soft capsules in the treatment of post-stroke epilepsy |
|  | Lin 2017 | Clinical efficacy analysis of combined Chinese and Western medicine in the treatment of epilepsy secondary to cerebral infarction |
|  | Liu 2014 | Effectiveness of combined Chinese and Western medicine in the treatment of late-onset epilepsy after cerebral infarction |
|  | Yang 2010 | Individualized staging and typing of ischemic post-stroke epilepsy in Chinese and Western medicine with syndrome differentiation treatment |
|  | Yang 2007 | Clinical observation of 45 cases of epilepsy secondary to cerebral infarction treated with combined Chinese and Western medicine |
| Not retrieved (n = 4) | Wu 2022 | Analysis of the cognitive function and clinical effects of levetiracetam and Yinao Capsule on post-stroke epilepsy patients |
|  | Wang 2021 | Clinical efficacy of combined Chinese and Western medicine in the treatment of patients with epilepsy secondary to post-stroke |
|  | Wu 2021 | A pilot study on the clinical management of secondary epilepsy after stroke |
|  | Li 2020 | The role and clinical effects of Huoxue Dingxian Formula in the identification and treatment of secondary epilepsy after cerebral infarction |
| Repeat publications (n = 1) | Liu 2011 | Preliminary observations on the treatment of epilepsy after cerebral infarction with a combination of Chinese and Western medicine |

## Supplementary Table S2. The detailed information of the CHMs prescribed

|  | **Study(publication year)** | **Formulation** | **Preparations** | **Ingredients of prescription** | **Quality control** | **Source** |
| --- | --- | --- | --- | --- | --- | --- |
| 1 | Zhai et al. (2022) | XZD + BBTD | Decoction | *Achyranthes bidentata* Blume [Amaranthaceae; Achyranthis bidentatae rhizoma] 20 g, *Conioselinum anthriscoides* 'Chuanxiong' [Apiaceae; Chuanxiong rhizoma] 15 g, *Pineilia ternata* (Thunb.) Makino [Araceae; Pinelliae rhizoma] 15 g, *Poria cocos* (Schw.) Wolf [Poria] 15 g, *Platycodon grandiflorus* (Jacq.) A.DC. [Campanulaceae; Platycodonis radix] 15 g, *Paeonia lactiflora* Pall. [Paeoniaceae; Paeoniae radix rubra] 15 g, *Atractylodes macrocephala* Koidz. [Asteraceae; Atractylodis macrocephalae rhizoma] 15 g, *Rehmannia glutinosa* (Gaertn.) DC. [Orobanchaceae; Rehmannia radix] 15 g, *Carthamus tinctorius* L. [Asteraceae; Carthami flos] 15 g, *Gastrodia elata* Blume [Orchidaceae; Gastrodiae rhizoma] 15 g, *Ziziphus jujuba* Mill. [Rhamnaceae; Jujubae fructus] 15 g, *Prunus persica* (L.) Batsch [Rosaceae; Persica semen] 15 g, *Citrus × aurantium f. deliciosa* (Ten.) M.Hiroe [Rutaceae; Citri reticulatae pericarpium] 15 g, *Angelica sinensis* (Oliv.) Diels [Apiaceae; Angelicae sinensis radix] 10 g, *Zingiber officinale* Roscoe [Zingiberaceae; Zingiberis rhizoma recens] 10 g, *Bupleurum falcatum* L. [Apiaceae; Bupleuri radix] 10 g. | N- | Prepared by Zhai et al., (2022) |
| 2 | Liang et al. (2022) | XDAP | Pills | *Coptis chinensis* Franch. [Ranunculaceae; Coptidis rhizoma], *Fritillaria cirrhosa* D.Don [Liliaceae; Fritillariae cirrhosae bulbus], *Poria cocos* (Schw.) Wolf [Poria], *Glycyrrhiza glabra* L. [Fabaceae; Glycyrrhizae radix et rhizoma], *Salvia miltiorrhiza* Bunge [Lamiaceae; Salviae miltiorrhizae radix et rhizoma], *Ophiopogon japonicus* (Thunb.) Ker Gawl. [Asparagaceae; Ophiopogonis radix]. | Y- | Quality controlled by Zhaoqing City Hospital of Traditional Chinese Medicine |
| 3 | Yang and Zhang (2022) | HTDD | Decoction | *Salvia miltiorrhiza* Bunge [Lamiaceae; Salviae miltiorrhizae radix et rhizoma] 16 g, *Polygala senega* L. [Polygalaceae; Polygalae radix] 16 g, *Gastrodia elata* Blume [Orchidaceae; Gastrodiae rhizoma] 16 g, *Poria cocos* (Schw.) Wolf [Poria] 12 g, *Atractylodes macrocephala* Koidz. [Asteraceae; Atractylodis macrocephalae rhizoma] 12 g, *Uncaria rhynchophylla* (Miq.) Miq. [Rubiaceae; Uncariae ramulus cum uncis] 12 g, *Acorus calamus var. angustatus* Besser [Acoraceae; Acori tatarinowii rhizoma] 12 g, *Ophiopogon japonicus* (Thunb.) Ker Gawl. [Asparagaceae; Ophiopogonis radix] 10 g, *Pineilia ternata* (Thunb.) Makino [Araceae; Pinelliae rhizoma praeparatum] 10 g, *Carthamus tinctorius* L. [Asteraceae; Carthami flos] 10 g, Succinum 10 g, *Codonopsis pilosula* (Franch.) Nannf. [Campanulaceae; Codonopsis radix] 10 g, *Buthus martensii* Karsch [Scorpio] 6 g, *Bombyx mori Linnaeus* [Bombyx batryticatus] 6 g, *Citrus × aurantium f. deliciosa* (Ten.) M.Hiroe [Rutaceae; Citri reticulatae pericarpium] 6 g, *Arisaema erubescens* (Wall.) Schott [Araceae; Arisaema cum bile] 6 g. | N- | Prepared by Yang and Zhang (2022) |
| 4 | Liu et al. (2021) | CLMD | Decoction | *Bupleurum falcatum* L. [Apiaceae; Bupleuri radix] 30 g, *Pineilia ternata* (Thunb.) Makino [Araceae; Pinelliae rhizoma] 15 g, *Rheum palmatum* L. [Polygonaceae; Rhei radix et rhizoma] 15 g, *Zingiber officinale* Roscoe [Zingiberaceae; Zingiberis rhizoma recens] 20 g, *Ostrea gigas* Thunberg [Ostreae concha] 20 g, Fossilia Ossia Mastodi [Os draconis] 20 g, *Scutellaria baicalensis* Georgi [Lamiaceae; Scutellariae radix] 20 g, *Panax ginseng* C.A.Mey. [Araliaceae; Ginseng radix et rhizoma] 20 g, *Neolitsea cassia* (L.) Kosterm. [Lauraceae; Cinnamomi ramulus] 20 g, *Poria cocos* (Schw.) Wolf [Poria] 20 g, *Ziziphus jujuba* Mill. [Rhamnaceae; Jujubae fructus] 6 pieces. | N- | Prepared by Liu et al., (2021) |
| 5 | Jin et al. (2020) | SV | Injection | Salvianolate | Y- | Prepared according to China of NMPA |
| 6 | Zhang et al. (2020) | XNJ | Injection | *Moschus berezovskii* Flerov [Moschus], *Curcuma longa* L. [Zingiberaceae; Curcumae radix], *Gardenia jasminoides* J.Ellis [Rubiaceae; Gardeniae fructus], (C_10_H_18_O) [Borneolum syntheticum]. | Y- | Prepared according to China of NMPA: Z53021639 |
| 7 | Liu et al. (2020) | KJD | Decoction | Fe_3_O_4_ [Magnetitum] 30 g, *Achyranthes bidentata* Blume [Amaranthaceae; Achyranthis bidentatae rhizoma] 30 g, *Poria cocos* (Schw.) Wolf [Poria] 30 g, *Arisaema erubescens* (Wall.) Schott [Araceae; Arisaema cum bile] 15 g, *Pheretima aspergillum* (E. Perrier) [Pheretima] 15 g, *Pineilia ternata* (Thunb.) Makino [Araceae; Pinelliae rhizoma praeparatum cum alumine] 12 g, *Bombyx mori Linnaeus* [Bombyx batryticatus] 10 g, *Curcuma longa* L. [Zingiberaceae; Curcumae longae rhizoma] 10 g, *Rheum palmatum* L. [Polygonaceae; Rhei radix et rhizoma] 10 g, *Citrus × aurantium f. deliciosa* (Ten.) M.Hiroe [Rutaceae; Citri exocarpium rubrum] 10 g, *Acorus calamus var. angustatus* Besser [Acoraceae; Acori tatarinowii rhizoma] 10 g, *Conioselinum anthriscoides* 'Chuanxiong' [Apiaceae; Chuanxiong rhizoma] 10 g, *Neolitsea cassia* (L.) Kosterm. [Lauraceae; Cinnamomi ramulus] 10 g, *Panax ginseng* C.A.Mey. [Araliaceae; Ginseng radix et rhizoma] 10 g, *Glycyrrhiza glabra* L. [Fabaceae; Glycyrrhizae radix et rhizoma] 10 g, *Cryptotympana pustulata* Fabricius [Cicadae periostracum] 6 g, *Aquilaria sinensis* (Lour.) Spreng. [Thymelaeaceae; Aquilariae lignum resinatum] 6 g. | N- | Prepared by Liu et al., (2020) |
| 8 | Chang and Cui (2020) | DXP + THD | Pills + Decoction | *Gastrodia elata* Blume [Orchidaceae; Gastrodiae rhizoma] 15 g, *Fritillaria cirrhosa* D.Don [Liliaceae; Fritillariae cirrhosae bulbus] 15 g, *Pineilia ternata* (Thunb.) Makino [Araceae; Pinelliae rhizoma praeparatum cum zingibere et alumine] 15 g, *Poria cocos* (Schw.) Wolf [Poria] 15 g, *Poria cocos* (Schw.) Wolf [Poria cum radix pini] 15 g, *Arisaema erubescens* (Wall.) Schott [Araceae; Arisaema cum bile] 15 g, *Acorus calamus var. angustatus* Besser [Acoraceae; Acori tatarinowii rhizoma] 15 g, *Pheretima aspergillum* (E. Perrier) [Pheretima] 15 g, *Buthus martensii* Karsch [Scorpio] 15 g, *Bombyx mori Linnaeus* [Bombyx batryticatus] 15 g, Succinum 15 g, *Carthamus tinctorius* L. [Asteraceae; Carthami flos] 12 g, *Conioselinum anthriscoides* 'Chuanxiong' [Apiaceae; Chuanxiong rhizoma] 12 g, *Paeonia lactiflora* Pall. [Paeoniaceae; Paeoniae radix rubra] 12 g, *Prunus persica* (L.) Batsch [Rosaceae; Persica semen] 12 g, *Citrus × aurantium f. deliciosa* (Ten.) M.Hiroe [Rutaceae; Citri reticulatae pericarpium] 12 g, *Polygala senega* L. [Polygalaceae; Polygalae radix] 12 g, *Salvia miltiorrhiza* Bunge [Lamiaceae; Salviae miltiorrhizae radix et rhizoma] 20 g, *Ophiopogon japonicus* (Thunb.) Ker Gawl. [Asparagaceae; Ophiopogonis radix] 20 g. | N- | Prepared by Chang and Cui (2020) |
| 9 | Li and Liu (2020) | BJP | CPM | *Curcuma longa* L. [Zingiberaceae; Curcumae radix], [KAl(SO_4_)_2_·12H_2_O] [Alumen], *Mentha canadensis* L. [Lamiaceae; Menthae haplocalycis herba]. | Y- | Prepared according to China of NMPA: Z20025543 |
| 10 | Niu (2019) | QWD | Decoction | *Pineilia ternata* (Thunb.) Makino [Araceae; Pinelliae rhizoma] 9 g, *Bambusa tuldoides* Munro [Poaceae; Bambusae caulis in taenias] 6 g, *Citrus × aurantium f. deliciosa* (Ten.) M.Hiroe [Rutaceae; Citri reticulatae pericarpium] 12 g, *Citrus × aurantium* L. [Rutaceae; aurantii fructus immaturus] 6 g, *Atractylodes macrocephala* Koidz. [Asteraceae; Atractylodis macrocephalae rhizoma] 15 g, *Poria cocos* (Schw.) Wolf [Poria] 12 g, *Coptis chinensis* Franch. [Ranunculaceae; Coptidis rhizoma] 6 g, *Cyperus rotundus* L. [Cyperaceae; Cyperi rhizoma] 12 g, *Acorus calamus var. angustatus* Besser [Acoraceae; Acori tatarinowii rhizoma] 9 g, *Polygala senega* L. [Polygalaceae; Polygalae radix] 9 g, *Conioselinum anthriscoides* 'Chuanxiong' [Apiaceae; Chuanxiong rhizoma] 10 g, *Panax ginseng* C.A.Mey. [Araliaceae; Ginseng radix et rhizoma] 6 g, *Ophiopogon japonicus* (Thunb.) Ker Gawl. [Asparagaceae; Ophiopogonis radix] 9 g, *Paeonia lactiflora* Pall. [Paeoniaceae; Paeoniae radix alba] 12 g. | N- | Prepared by Niu (2019) |
| 11 | Jiao (2018) | XZD + BBTD | Pills | *Bupleurum falcatum* L. [Apiaceae; Bupleuri radix], *Angelica sinensis* (Oliv.) Diels [Apiaceae; Angelicae sinensis radix], *Carthamus tinctorius* L. [Asteraceae; Carthami flos], *Prunus persica* (L.) Batsch [Rosaceae; Persica semen], *Paeonia lactiflora* Pall. [Paeoniaceae; Paeoniae radix rubra], *Citrus × aurantium* L. [Rutaceae; aurantii fructus], *Achyranthes bidentata* Blume [Amaranthaceae; Achyranthis bidentatae rhizoma], *Rehmannia glutinosa* (Gaertn.) DC. [Orobanchaceae; Rehmannia radix], *Pineilia ternata* (Thunb.) Makino [Araceae; Pinelliae rhizoma], *Gastrodia elata* Blume [Orchidaceae; Gastrodiae rhizoma], *Poria cocos* (Schw.) Wolf [Poria], *Buthus martensii* Karsch [Scorpio], *Citrus × aurantium f. deliciosa* (Ten.) M.Hiroe [Rutaceae; Citri exocarpium rubrum], *Atractylodes macrocephala* Koidz. [Asteraceae; Atractylodis macrocephalae rhizoma], *Acorus calamus var. angustatus* Besser [Acoraceae; Acori tatarinowii rhizoma], *Bombyx mori Linnaeus* [Bombyx batryticatus]. | N- | Prepared by Jiao (2018) |
| 12 | Yu (2018) | XNJ | Injection | *Moschus berezovskii* Flerov [Moschus], *Curcuma longa* L. [Zingiberaceae; Curcumae radix], *Gardenia jasminoides* J.Ellis [Rubiaceae; Gardeniae fructus], (C_10_H_18_O) [Borneolum syntheticum]. | Y- | Prepared according to China of NMPA: Z53021640 |
| 13 | Deng et al. (2018) | XNJ | Injection | *Moschus berezovskii* Flerov [Moschus], *Curcuma longa* L. [Zingiberaceae; Curcumae radix], *Gardenia jasminoides* J.Ellis [Rubiaceae; Gardeniae fructus], (C_10_H_18_O) [Borneolum syntheticum]. | Y- | Prepared according to China of NMPA: Z41020664 |
| 14 | Li et al. (2018) | QWD | Decoction | *Ophiopogon japonicus* (Thunb.) Ker Gawl. [Asparagaceae; Ophiopogonis radix] 2.4 g, *Conioselinum anthriscoides* 'Chuanxiong' [Apiaceae; Chuanxiong rhizoma] 1.8 g, *Panax ginseng* C.A.Mey. [Araliaceae; Ginseng radix et rhizoma] 1.8 g, *Polygala senega* L. [Polygalaceae; Polygalae radix] 1.8 g, *Glycyrrhiza glabra* L. [Fabaceae; Glycyrrhizae radix et rhizoma] 1.2 g, *Angelica sinensis* (Oliv.) Diels [Apiaceae; Angelicae sinensis radix] 3 g, *Paeonia lactiflora* Pall. [Paeoniaceae; Paeoniae radix alba] 3 g, *Atractylodes macrocephala* Koidz. [Asteraceae; Atractylodis macrocephalae rhizoma] 3 g, *Poria cocos* (Schw.) Wolf [Poria] 3 g, *Citrus × aurantium f. deliciosa* (Ten.) M.Hiroe [Rutaceae; Citri reticulatae pericarpium] 3 g, *Pineilia ternata* (Thunb.) Makino [Araceae; Pinelliae rhizoma] 3 g, *Citrus × aurantium* L. [Rutaceae; aurantii fructus immaturus] 3 g, *Bambusa tuldoides* Munro [Poaceae; Bambusae caulis in taenias] 3 g, *Acorus calamus var. angustatus* Besser [Acoraceae; Acori tatarinowii rhizoma] 3 g, *Cyperus rotundus* L. [Cyperaceae; Cyperi rhizoma] 3 g, *Coptis chinensis* Franch. [Ranunculaceae; Coptidis rhizoma] 3 g. | N- | Prepared by Li et al., (2018) |
| 15 | Liu and Wang (2018) | CLMD | Decoction | *Bupleurum falcatum* L. [Apiaceae; Bupleuri radix] 15 g, *Scutellaria baicalensis* Georgi [Lamiaceae; Scutellariae radix] 9 g, *Pineilia ternata* (Thunb.) Makino [Araceae; Pinelliae rhizoma praeparatum] 12 g, *Codonopsis pilosula* (Franch.) Nannf. [Campanulaceae; Codonopsis radix] 15 g, *Neolitsea cassia* (L.) Kosterm. [Lauraceae; Cinnamomi ramulus] 12 g, *Poria cocos* (Schw.) Wolf [Poria] 12 g, *Atractylodes macrocephala* Koidz. [Asteraceae; Atractylodis macrocephalae rhizoma] 12 g, Fossilia Ossia Mastodi [Os draconis] 30 g, *Ostrea gigas* Thunberg [Ostreae concha] 30 g, *Rheum palmatum* L. [Polygonaceae; Rhei radix et rhizoma] 6 g, *Prunus persica* (L.) Batsch [Rosaceae; Persica semen] 6 g, *Carthamus tinctorius* L. [Asteraceae; Carthami flos] 6 g, *Polygala senega* L. [Polygalaceae; Polygalae radix] 9 g, *Acorus calamus var. angustatus* Besser [Acoraceae; Acori tatarinowii rhizoma] 9 g, *Trichosanthes kirilowii* Maxim. [Cucurbitaceae; Trichosanthis fructus] 15 g, *Paeonia lactiflora* Pall. [Paeoniaceae; Paeoniae radix alba] 12 g, *Zingiber officinale* Roscoe [Zingiberaceae; Zingiberis rhizoma recens] 6 g, *Ziziphus jujuba* Mill. [Rhamnaceae; Jujubae fructus] 5 pieces. | N- | Prepared by Liu and Wang (2018) |
| 16 | Guo (2017) | XNJ | Injection | *Moschus berezovskii* Flerov [Moschus], *Curcuma longa* L. [Zingiberaceae; Curcumae radix], *Gardenia jasminoides* J.Ellis [Rubiaceae; Gardeniae fructus], (C_10_H_18_O) [Borneolum syntheticum]. | Y- | Prepared according to China of NMPA: Z53021639 |
| 17 | Wang and Zhang (2017) | DTD | Decoction | *Citrus × aurantium f. deliciosa* (Ten.) M.Hiroe [Rutaceae; Citri exocarpium rubrum] 15 g, *Curcuma longa* L. [Zingiberaceae; Curcumae radix] 15 g, *Conioselinum anthriscoides* 'Chuanxiong' [Apiaceae; Chuanxiong rhizoma] 15 g, *Angelica sinensis* (Oliv.) Diels [Apiaceae; Angelicae sinensis radix] 15 g, *Prunus persica* (L.) Batsch [Rosaceae; Persica semen] 15 g, *Carthamus tinctorius* L. [Asteraceae; Carthami flos] 15 g, *Paeonia lactiflora* Pall. [Paeoniaceae; Paeoniae radix rubra] 10 g, *Pheretima aspergillum* (E. Perrier) [Pheretima] 10 g, *Pineilia ternata* (Thunb.) Makino [Araceae; Pinelliae rhizoma] 10 g. | N- | Prepared by Wang and Zhang (2017) |
| 18 | Wang et al. (2015) | XNJ | Injection | *Moschus berezovskii* Flerov [Moschus], *Curcuma longa* L. [Zingiberaceae; Curcumae radix], *Gardenia jasminoides* J.Ellis [Rubiaceae; Gardeniae fructus], (C_10_H_18_O) [Borneolum syntheticum]. | Y- | Prepared according to China of NMPA: Z41020664 |
| 19 | Zhang (2015) | XNJ | Injection | *Moschus berezovskii* Flerov [Moschus], *Curcuma longa* L. [Zingiberaceae; Curcumae radix], *Gardenia jasminoides* J.Ellis [Rubiaceae; Gardeniae fructus], (C_10_H_18_O) [Borneolum syntheticum]. | Y- | Prepared according to China of NMPA: Z53021638 |
| 20 | Wang (2014) | XNJ | Injection | *Moschus berezovskii* Flerov [Moschus], *Curcuma longa* L. [Zingiberaceae; Curcumae radix], *Gardenia jasminoides* J.Ellis [Rubiaceae; Gardeniae fructus], (C_10_H_18_O) [Borneolum syntheticum]. | Y- | Prepared according to China of NMPA |
| 21 | Wang et al. (2014) | PXG | Granule | *Arisaema erubescens* (Wall.) Schott [Araceae; Arisaema cum bile] 18 g, (CaSO_4_ · 2H_2_O) [Gypsum fibrosum] 20 g, Fe_3_O_4_ [Magnetitum] 20 g, *Gastrodia elata* Blume [Orchidaceae; Gastrodiae rhizoma] 30 g, *Uncaria rhynchophylla* (Miq.) Miq. [Rubiaceae; Uncariae ramulus cum uncis] 20 g, *Cryptotympana pustulata* Fabricius [Cicadae periostracum] 6 g, *Bombyx mori Linnaeus* [Bombyx batryticatus] 10 g, *Buthus martensii* Karsch [Scorpio] 3 g, *Scolopendra subspinipes mutilans* L. Koch [Scolopendra] 2 strips, *Mentha canadensis* L. [Lamiaceae; Menthae haplocalycis herba] 10 g, *Wurfbainia vera* (Blackw.) Škorničk. & A.D.Poulsen [Zingiberaceae; Amomi fructus rotundus] 15 g, Succinum 12 g, *Acorus calamus var. angustatus* Besser [Acoraceae; Acori tatarinowii rhizoma] 10 g, *Aquilaria sinensis* (Lour.) Spreng. [Thymelaeaceae; Aquilariae lignum resinatum] 6 g, *Conioselinum anthriscoides* 'Chuanxiong' [Apiaceae; Chuanxiong rhizoma] 8 g, *Panax ginseng* C.A.Mey. [Araliaceae; Ginseng radix et rhizoma] 12 g, Dried Human Placenta [Hominis placenta] 12 g, *Diospyros kaki* Thunb. [Mannosum kaki] 30 g. | N- | Prepared by Wang et al., (2014) |
| 22 | Cai et al. (2013) | TTC | CPM | *Conioselinum anthriscoides* 'Chuanxiong' [Apiaceae; Chuanxiong rhizoma], *Sigesbeckia orientalis* L. [Asteraceae; Sigesbeckiae herba], *Salvia miltiorrhiza* Bunge [Lamiaceae; Salviae miltiorrhizae radix et rhizoma], *Whitmania pigra* Whitman [Hirudo], *Gastrodia elata* Blume [Orchidaceae; Gastrodiae rhizoma], *Styphnolobium japonicum* (L.) Schott [Fabaceae; Sophorae], *Acorus calamus var. angustatus* Besser [Acoraceae; Acori tatarinowii rhizoma], *Bos taurus domesticus* Gmelin [Bovis calculus], *Astragalus mongholicus* Bunge [Fabaceae; Astragali radix], *Achyranthes bidentata* Blume [Amaranthaceae; Achyranthis bidentatae rhizoma]. | Y- | Prepared according to China of NMPA: Z20010029 |
| 23 | Liu and Yin (2008) | HCD + HLC | Granule + Decoction | *Astragalus mongholicus* Bunge [Fabaceae; Astragali radix] 60 g, *Paeonia lactiflora* Pall. [Paeoniaceae; Paeoniae radix rubra] 15 g, *Saposhnikovia divaricata* (Turcz. ex Ledeb.) Schischk. [Apiaceae; Saposhnikoviae radix] 15 g, *Pheretima aspergillum* (E. Perrier) [Pheretima] 10 g, *Spatholobus suberectus* Dunn [Fabaceae; Spatholobi caulis] 30 g, *Codonopsis pilosula* (Franch.) Nannf. [Campanulaceae; Codonopsis radix] 30 g, *Curcuma longa* L. [Zingiberaceae; Curcumae radix] 9 g, *Conioselinum anthriscoides* 'Chuanxiong' [Apiaceae; Chuanxiong rhizoma] 15 g. + *Bombyx mori Linnaeus* [Bombyx batryticatus], *Buthus martensii* Karsch [Scorpio], *Scolopendra subspinipes mutilans* L. Koch [Scolopendra]. | Y- | Prepared by Liu and Yin (2008) + Quality controlled by Handan City Hospital of Traditional Chinese Medicine |

Note: NMPA: National Medical Products Administration; N: no; Y: yes; g, gram; CPM: Chinese patent medicine; XZD: xuefu zhuyu decoction; BBTD: banxia baizhu tianma decoction; XDAP: xiandean pills; HTDD: huatan tongluo dingxian decoction; CLMD: chaihu longgu muli decoction; XNJ: xingnaojing injection; SV: salvianolate injection; QWD: qingxin wendan decoction; DTD: ditan decoction; KJD: kangxian jiejing decoction; BJP: baijin pills; PXG: pingxian granule; TTC: tiandan tongluo capsules; DXP: dingxian pills; THD: tongqiao huoxue decoction; HCD: huangqi chifeng decoction; HLC: huoluo capsules.

## Supplementary Table S3. Trim-and-fill test of the total responder rate in comparison of orally administered CHM plus CWM vs. CWM.

| **Outcome** | **Effect-size** | **Effect model** | **Before trim-and-fill** | | **After trim-and-fill** | | **Increased research** |
| --- | --- | --- | --- | --- | --- | --- | --- |
|  |  |  | **Pooled estimate** | **95％CI** | **Pooled estimate** | **95％CI** |  |
| Orally administered CHM plus CWM vs. CWM | *RR* | FE | 0.237 | 0.173 to 0.301 | 1.225 | 1.157 to 1.297 | 5 |
|  |  | RE | 0.237 | 0.173 to 0.301 | 1.225 | 1.157 to 1.297 |  |

Note: CHM, Chinese herbal medicine; CWM, conventional ; FE, fixed-effects; RE, random-effects; *RR*, risk ratio.

## Supplementary Table S4. Frequency ranking of Chinese herbal medicine.

| Chinese herbal medicine | frequency | Chinese herbal medicine | frequency | Chinese herbal medicine | frequency |
| --- | --- | --- | --- | --- | --- |
| *Pineilia ternata* (Thunb.) Makino [Araceae; Pinelliae rhizoma] | 9 | *Rheum palmatum* L. [Polygonaceae; Rhei radix et rhizoma] | 3 | *Scolopendra subspinipes mutilans* L. Koch [Scolopendra] | 2 |
| *Poria cocos* (Schw.) Wolf [Poria] | 9 | *Ziziphus jujuba* Mill. [Rhamnaceae; Jujubae fructus] | 3 | *Cyperus rotundus* L. [Cyperaceae; Cyperi rhizoma] | 2 |
| *Conioselinum anthriscoides* 'Chuanxiong' [Apiaceae; Chuanxiong rhizoma] | 8 | *Salvia miltiorrhiza* Bunge [Lamiaceae; Salviae miltiorrhizae radix et rhizoma] | 3 | *Curcuma longa* L. [Zingiberaceae; Curcumae radix] | 2 |
| *Acorus calamus var. angustatus* Besser [Acoraceae; Acori tatarinowii rhizoma] | 7 | *Angelica sinensis* (Oliv.) Diels [Apiaceae; Angelicae sinensis radix] | 3 | *Citrus × aurantium* L. [Rutaceae; aurantii fructus immaturus] | 2 |
| *Atractylodes macrocephala* Koidz. [Asteraceae; Atractylodis macrocephalae rhizoma] | 5 | *Codonopsis pilosula* (Franch.) Nannf. [Campanulaceae; Codonopsis radix] | 3 | *Bambusa tuldoides* Munro [Poaceae; Bambusae caulis in taenias] | 2 |
| *Citrus × aurantium f. deliciosa* (Ten.) M.Hiroe [Rutaceae; Citri reticulatae pericarpium] | 5 | *Glycyrrhiza glabra* L. [Fabaceae; Glycyrrhizae radix et rhizoma] | 3 | *Ostrea gigas* Thunberg [Ostreae concha] | 2 |
| *Carthamus tinctorius* L. [Asteraceae; Carthami flos] | 5 | *Neolitsea cassia* (L.) Kosterm. [Lauraceae; Cinnamomi ramulus] | 3 | *Wurfbainia vera* (Blackw.) Škorničk. & A.D.Poulsen [Zingiberaceae; Amomi fructus rotundus] | 1 |
| *Bombyx mori Linnaeus* [Bombyx batryticatus] | 5 | SUCCINUM | 3 | *Mentha canadensis* L. [Lamiaceae; Menthae haplocalycis herba] | 1 |
| *Ophiopogon japonicus* (Thunb.) Ker Gawl. [Asparagaceae; Ophiopogonis radix] | 5 | *Coptis chinensis* Franch. [Ranunculaceae; Coptidis rhizoma] | 3 | *Saposhnikovia divaricata* (Turcz. ex Ledeb.) Schischk. [Apiaceae; Saposhnikoviae radix] | 1 |
| *Panax ginseng* C.A.Mey. [Araliaceae; Ginseng radix et rhizoma] | 5 | *Zingiber officinale* Roscoe [Zingiberaceae; Zingiberis rhizoma recens] | 3 | *Poria cocos* (Schw.) Wolf [Poria cum radix pini] | 1 |
| *Polygala senega* L. [Polygalaceae; Polygalae radix] | 5 | *Cryptotympana pustulata* Fabricius [Cicadae periostracum] | 2 | *Trichosanthes kirilowii* Maxim. [Cucurbitaceae; Trichosanthis fructus] | 1 |
| *Paeonia lactiflora* Pall. [Paeoniaceae; Paeoniae radix rubra] | 4 | *Aquilaria sinensis* (Lour.) Spreng. [Thymelaeaceae; Aquilariae lignum resinatum] | 2 | *Spatholobus suberectus* Dunn [Fabaceae; Spatholobi caulis] | 1 |
| *Arisaema erubescens* (Wall.) Schott [Araceae; Arisaema cum bile] | 4 | *Fritillaria cirrhosa* D.Don [Liliaceae; Fritillariae cirrhosae bulbus] | 2 | *Curcuma longa* L. [Zingiberaceae; Curcumae longae rhizoma] | 1 |
| *Pheretima aspergillum* (E. Perrier) [Pheretima] | 4 | Fe_3_O_4_ [Magnetitum] | 2 | *Platycodon grandiflorus* (Jacq.) A.DC. [Campanulaceae; Platycodonis radix] | 1 |
| *Buthus martensii* Karsch [Scorpio] | 4 | *Uncaria rhynchophylla* (Miq.) Miq. [Rubiaceae; Uncariae ramulus cum uncis] | 2 | *Rehmannia glutinosa* (Gaertn.) DC. [Orobanchaceae; Rehmannia radix] | 1 |
| *Prunus persica* (L.) Batsch [Rosaceae; Persica semen] | 4 | *Scutellaria baicalensis* Georgi [Lamiaceae; Scutellariae radix] | 2 | *Astragalus mongholicus* Bunge [Fabaceae; Astragali radix] | 1 |
| *Gastrodia elata* Blume [Orchidaceae; Gastrodiae rhizoma] | 4 | *Citrus × aurantium f. deliciosa* (Ten.) M.Hiroe [Rutaceae; Citri exocarpium rubrum] | 2 | (CaSO_4_ · 2H_2_O) [Gypsum fibrosum] | 1 |
| *Paeonia lactiflora* Pall. [Paeoniaceae; Paeoniae radix alba] | 3 | Fossilia Ossia Mastodi [Os draconis] | 2 | *Diospyros kaki* Thunb. [Mannosum kaki] | 1 |
| *Bupleurum falcatum* L. [Apiaceae; Bupleuri radix] | 3 | *Achyranthes bidentata* Blume [Amaranthaceae; Achyranthis bidentatae rhizoma] | 2 | Dried Human Placenta [Hominis placenta] | 1 |

# Supplementary Figures

## Supplementary Figure S1. Sensitivity analysis


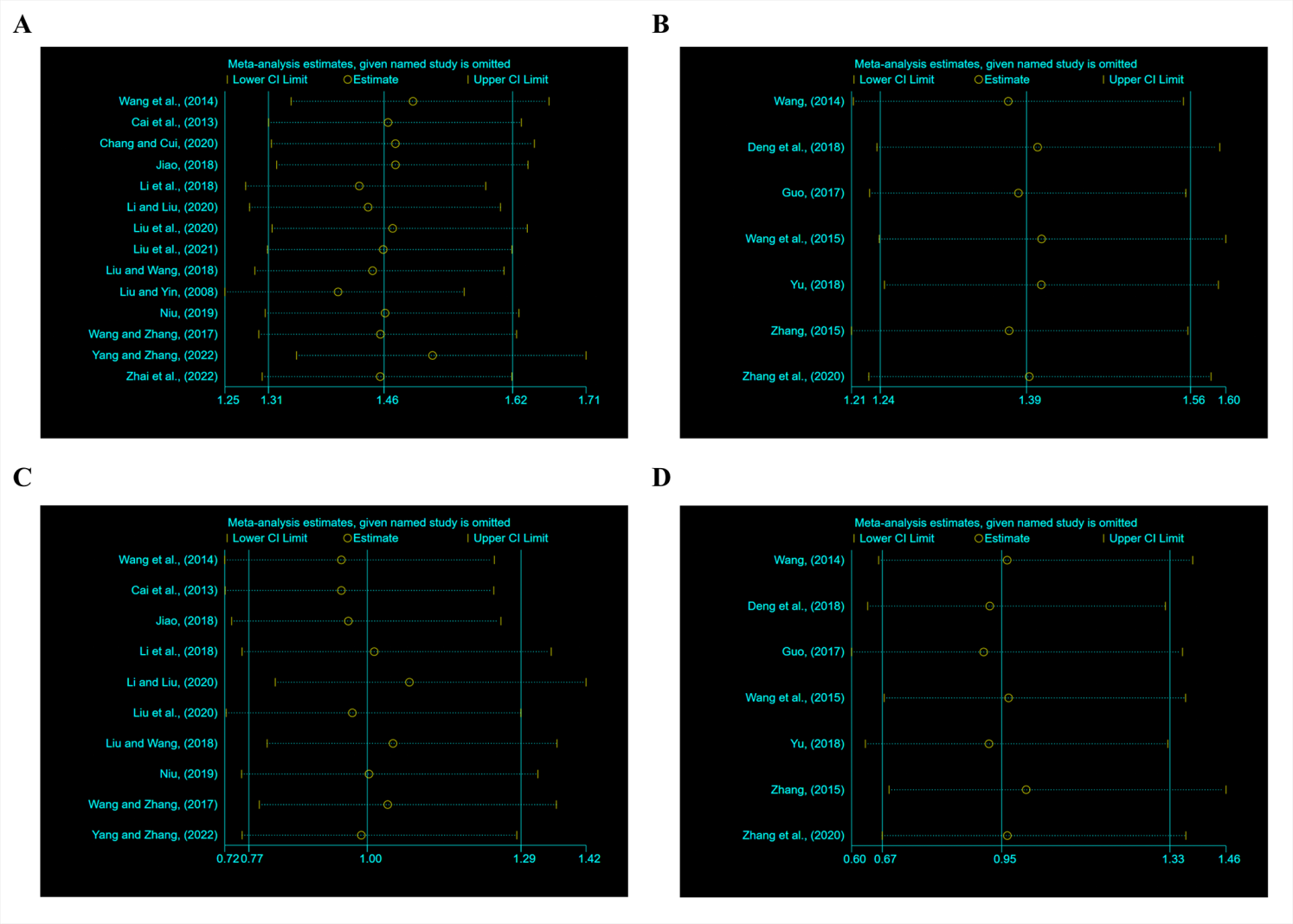


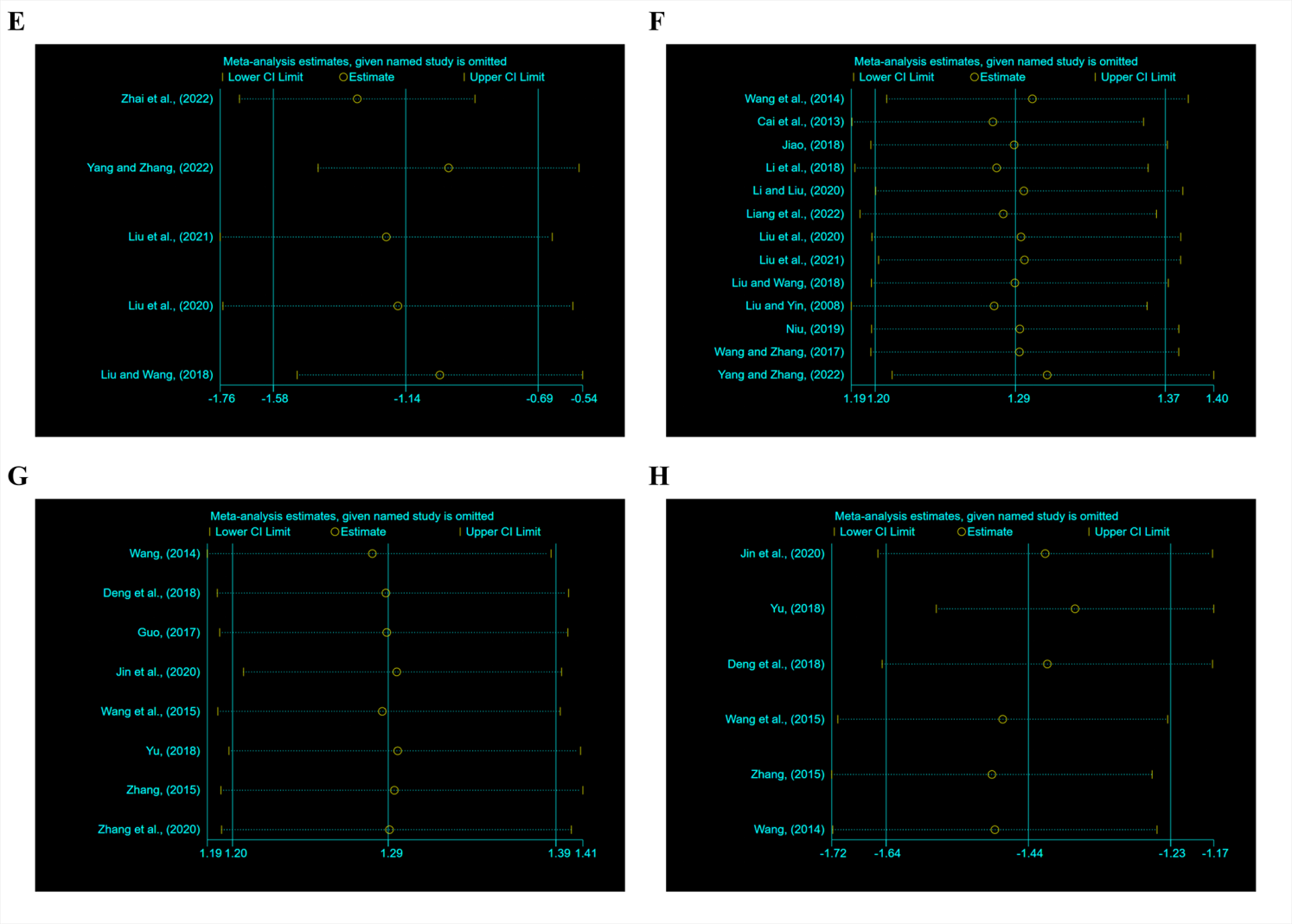


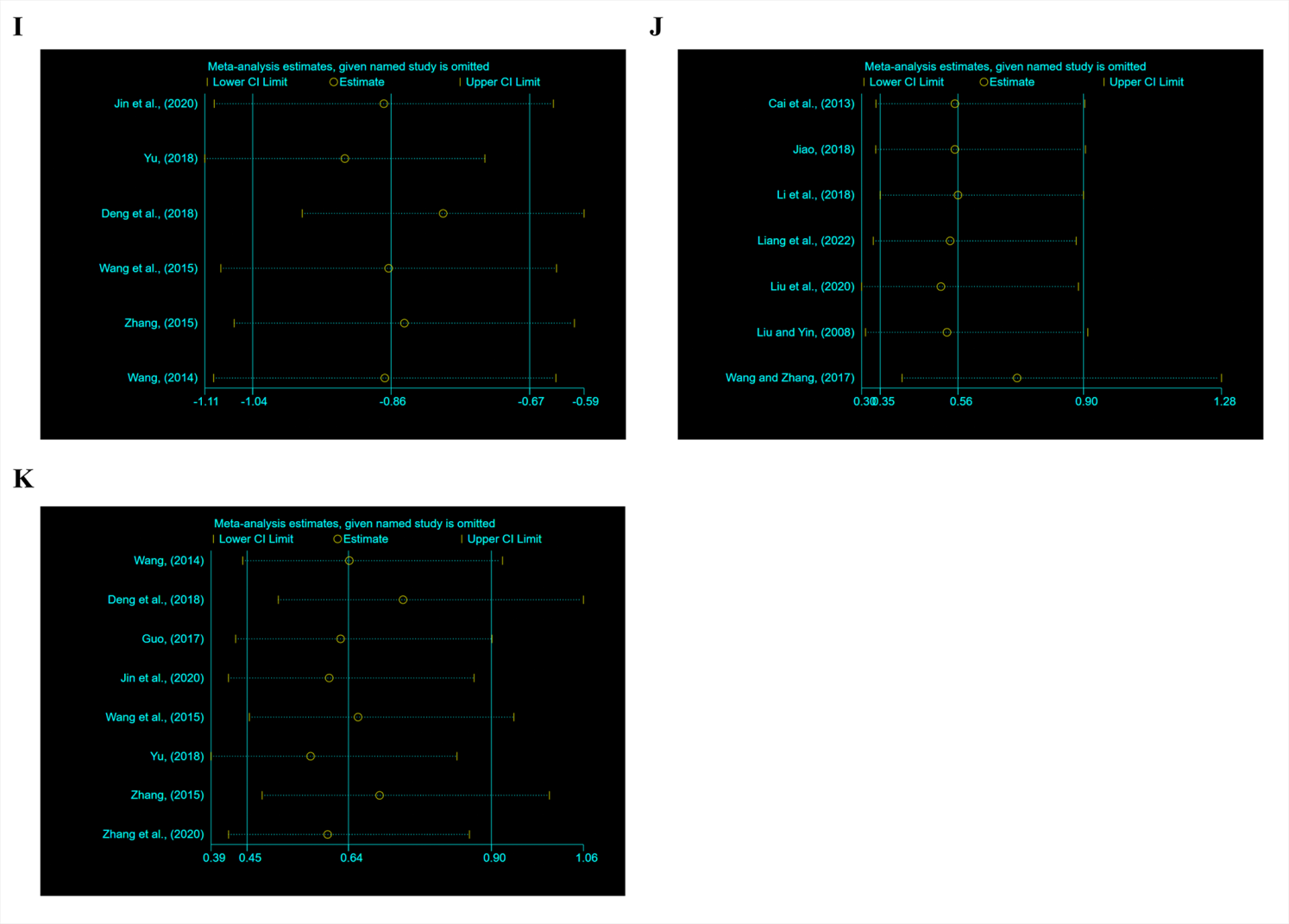


**(A)** 75% responder rate in comparison of orally administered CHM plus CWM vs. CWM;

**(B)** 75% responder rate in comparison of intravenously administered CHM plus CWM vs. CWM;

**(C)** 50%~75% responder rate in comparison of orally administered CHM plus CWM vs. CWM;

**(D)** 50%~75% responder rate in comparison of intravenously administered CHM plus CWM vs. CWM;

**(E)** Seizure duration in comparison of orally administered CHM plus CWM vs. CWM;

**(F)** Total responder rate in comparison of orally administered CHM plus CWM vs. CWM;

**(G)** Total responder rate in comparison of intravenously administered CHM plus CWM vs. CWM;

**(H)** Epileptiform discharges in comparison of intravenously administered CHM plus CWM vs. CWM;

**(I)** The number of leads involved in epileptiform discharge in comparison of intravenously administered CHM plus CWM vs. CWM;

**(J)** The incidence of AEs in comparison of orally administered CHM plus CWM vs. CWM;

**(K)** The incidence of AEs in comparison of intravenously administered CHM plus CWM vs. CWM.

## Supplementary Figure S2. Egger’s plot


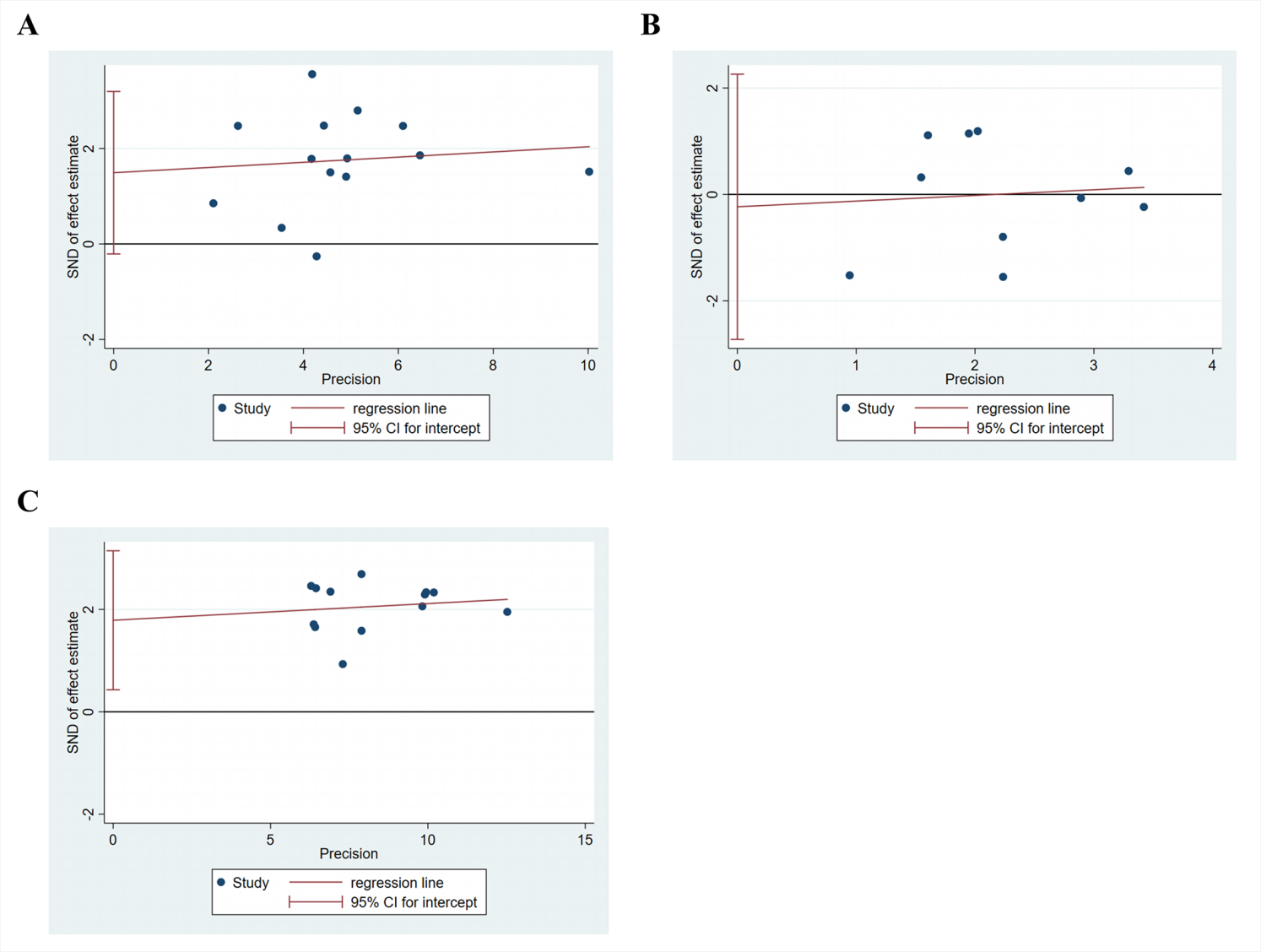


**(A)** 75% responder rate in comparison of orally administered CHM plus CWM vs. CWM;

**(B)** 50%~75% responder rate in comparison of orally administered CHM plus CWM vs. CWM;

**(C)** Total responder rate in comparison of orally administered CHM plus CWM vs. CWM.

# Supplementary File

## Supplementary File S1. Search strategy

**CNKI**

((SU=('癫痫' + '羊角风' + '羊癫风' + '痫病' + '痫症' + '痫证' + '羊癫疯' + '痫性发作' + '惊厥') AND SU=('脑出血' + '脑梗死' + '脑梗塞' + '卒中' + '中风' + '脑栓塞' + '蛛网膜下腔出血' + '脑血栓形成' + '脑溢血' + '脑血管意外')) OR SU=('卒中后癫痫' + '中风后癫痫')) AND SU=('中医' + '中药' + '中医药' + '中西医' + '中西药' + '草药' + '中草药' + '中成药' + '方' + '汤' + '片' + '丸' + '散' + '胶囊' + '颗粒' + '水' + '液' + '合剂' + '口服液' + '注射液' + '粉' + '丹' + '复方' + '膏' + '酒' + '茶' + '露' + '传统医疗' + '替代疗法' + '补充疗法') AND AB=('随机对照试验' + '随机对照研究' + 'RCT' + '随机' + '对照' + '控制组' + '安慰剂' + '试验' + '分组' + '临床' + '临床研究' + '临床疗效')

n= 256

**VIP**

(M=((("癫痫" OR "羊角风" OR "羊癫风" OR "痫病" OR "痫症" OR "痫证" OR "羊癫疯" OR "痫性发作" OR "惊厥") AND ("脑出血" OR "脑梗死" OR "脑梗塞" OR "卒中" OR "中风" OR "脑栓塞" OR "蛛网膜下腔出血" OR "脑血栓形成" OR "脑溢血" OR "脑血管意外")) OR ("卒中后癫痫" OR "中风后癫痫")) AND ("中医" OR "中药" OR "中医药" OR "中西医" OR "中西药" OR "草药" OR "中草药" OR "中成药" OR "方" OR "汤" OR "片" OR "丸" OR "散" OR "胶囊" OR "颗粒" OR "水" OR "液" OR "合剂" OR "口服液" OR "注射液" OR "粉" OR "丹" OR "复方" OR "膏" OR "酒" OR "茶" OR "露" OR "传统医疗" OR "替代疗法" OR "补充疗法")) AND ("随机对照试验" OR "随机对照研究" OR "RCT" OR "随机" OR "对照" OR "控制组" OR "安慰剂" OR "试验" OR "分组" OR "临床" OR "临床研究" OR "临床疗效")

n= 78

**Wanfang**

检索表达式： ((主题:("癫痫" OR "羊角风" OR "羊癫风" OR "痫病" OR "痫症" OR "痫证" OR "羊癫疯" OR "痫性发作" OR "惊厥") and 主题:("脑出血" OR "脑梗死" OR "脑梗塞" OR "卒中" OR "中风" OR "脑栓塞" OR "蛛网膜下腔出血" OR "脑血栓形成" OR "脑溢血" OR "脑血管意外")) or 主题:("卒中后癫痫" OR "中风后癫痫")) and 主题:("中医" OR "中药" OR "中医药" OR "中西医" OR "中西药" OR "草药" OR "中草药" OR "中成药" OR "方" OR "汤" OR "片" OR "丸" OR "散" OR "胶囊" OR "颗粒" OR "水" OR "液" OR "合剂" OR "口服液" OR "注射液" OR "粉" OR "丹" OR "复方" OR "膏" OR "酒" OR "茶" OR "露" OR "传统医疗" OR "替代疗法" OR "补充疗法") and 摘要:("随机对照试验" OR "随机对照研究" OR "RCT" OR "随机" OR "对照" OR "控制组" OR "安慰剂" OR "试验" OR "分组" OR "临床" OR "临床研究" OR "临床疗效")

n=3,379

**SinoMed**

(("随机对照试验"[常用字段:智能] OR "随机对照研究"[常用字段:智能] OR "RCT"[常用字段:智能] OR "随机"[常用字段:智能] OR "对照"[常用字段:智能] OR "控制组"[常用字段:智能] OR "安慰剂"[常用字段:智能] OR "试验"[常用字段:智能] OR "分组"[常用字段:智能] OR "临床"[常用字段:智能] OR "临床研究"[常用字段:智能] OR "临床疗效"[常用字段:智能]) OR ("随机对照试验"[不加权:扩展] OR "随机对照试验(主题)"[不加权:扩展])) AND (("中医"[常用字段:智能] OR "中药"[常用字段:智能] OR "中医药"[常用字段:智能] OR "中西医"[常用字段:智能] OR "中西药"[常用字段:智能] OR "草药"[常用字段:智能] OR "中草药"[常用字段:智能] OR "中成药"[常用字段:智能] OR "方"[常用字段:智能] OR "汤"[常用字段:智能] OR "片"[常用字段:智能] OR "丸"[常用字段:智能] OR "散"[常用字段:智能] OR "胶囊"[常用字段:智能] OR "颗粒"[常用字段:智能] OR "水"[常用字段:智能] OR "液"[常用字段:智能] OR "合剂"[常用字段:智能] OR "口服液"[常用字段:智能] OR "注射液"[常用字段:智能] OR "粉"[常用字段:智能] OR "丹"[常用字段:智能] OR "复方"[常用字段:智能] OR "膏"[常用字段:智能] OR "酒"[常用字段:智能] OR "茶"[常用字段:智能] OR "露"[常用字段:智能] OR "传统医疗"[常用字段:智能] OR "替代疗法"[常用字段:智能] OR "补充疗法"[常用字段:智能]) OR (("医学, 中国传统"[不加权:扩展] OR "中药"[不加权:扩展] OR "中草药"[不加权:扩展] OR "中医药学"[不加权:扩展] OR "中医药疗法"[不加权:扩展] OR "中西医结合"[不加权:扩展] OR "中西医结合疗法"[不加权:扩展]) OR ("植物, 药用"[不加权:扩展] OR "中成药"[不加权:扩展] OR "复方"[不加权:扩展] OR "补充疗法"[不加权:扩展] OR "医学, 中国传统"[不加权:扩展] OR "中医学"[不加权:扩展]))) AND ((( "卒中后癫痫"[常用字段:智能] OR "中风后癫痫"[常用字段:智能])) OR (((( "脑出血"[常用字段:智能] OR "颅内出血"[常用字段:智能] OR "脑梗死"[常用字段:智能] OR "中风"[常用字段:智能] OR "卒中"[常用字段:智能] OR "缺血性卒中"[常用字段:智能] OR "出血性卒中"[常用字段:智能] OR "栓塞性卒中"[常用字段:智能] OR "颅内栓塞"[常用字段:智能] OR "颅内栓塞和血栓形成"[常用字段:智能] OR "蛛网膜下腔出血"[常用字段:智能] OR "脑梗塞"[常用字段:智能] OR "脑栓塞"[常用字段:智能] OR "脑血栓形成"[常用字段:智能] OR "脑溢血"[常用字段:智能] OR "脑血管意外"[常用字段:智能])) OR (("出血性卒中"[不加权:扩展] OR "栓塞性卒中"[不加权:扩展] OR "颅内栓塞"[不加权:扩展] OR "颅内栓塞和血栓形成"[不加权:扩展] OR "蛛网膜下腔出血"[不加权:扩展]) OR ("脑出血"[不加权:扩展] OR "颅内出血"[不加权:扩展] OR "脑梗死"[不加权:扩展] OR "中风"[不加权:扩展] OR "卒中"[不加权:扩展] OR "缺血性卒中"[不加权:扩展]))) AND ((( "癫痫"[常用字段:智能] OR "羊角风"[常用字段:智能] OR "羊癫风"[常用字段:智能] OR "痫病"[常用字段:智能] OR "痫症"[常用字段:智能] OR "痫证"[常用字段:智能] OR "羊癫疯"[常用字段:智能] OR "痫性发作"[常用字段:智能] OR "惊厥"[常用字段:智能])) OR (("癫痫"[不加权:扩展]) OR "癫痫"[不加权:扩展]))))

11169

2023-10-11 22:29:35.0

n=11,169

**PubMed/**

((((((((("Epilepsy"[Mesh]) OR "Seizures"[Mesh]) OR "Epilepsy, Absence"[Mesh]) OR (((((((((((("Epilepsy"[Title/Abstract]) OR ("Epilepsies"[Title/Abstract])) OR ("Seizure Disorder"[Title/Abstract])) OR ("Seizure Disorders"[Title/Abstract])) OR ("Awakening Epilepsy"[Title/Abstract])) OR ("Epilepsy, Awakening"[Title/Abstract])) OR ("Epilepsy, Cryptogenic"[Title/Abstract])) OR ("Cryptogenic Epilepsies"[Title/Abstract])) OR ("Cryptogenic Epilepsy"[Title/Abstract])) OR ("Epilepsies, Cryptogenic"[Title/Abstract])) OR ("Aura"[Title/Abstract])) OR ("Auras"[Title/Abstract]))) OR (((((((((((((((((((((((((((((((((((((((((((((((((((((((((((((((((((((((((((((((((((((((((((((((((((((((((((((((((((((((((((((((("seizures"[Title/Abstract]) OR ("Seizure"[Title/Abstract])) OR ("Atonic Absence Seizures"[Title/Abstract])) OR ("Atonic Absence Seizure"[Title/Abstract])) OR ("Absence Seizure, Atonic"[Title/Abstract])) OR ("Absence Seizures, Atonic"[Title/Abstract])) OR ("Seizure, Atonic Absence"[Title/Abstract])) OR ("Seizures, Sensory"[Title/Abstract])) OR ("Seizure, Sensory"[Title/Abstract])) OR ("Sensory Seizure"[Title/Abstract])) OR ("Sensory Seizures"[Title/Abstract])) OR ("Absence Seizures"[Title/Abstract])) OR ("Petit Mal Convulsion"[Title/Abstract])) OR ("Convulsion, Petit Mal"[Title/Abstract])) OR ("Absence Seizure"[Title/Abstract])) OR ("Seizure, Absence"[Title/Abstract])) OR ("Convulsions"[Title/Abstract])) OR ("Convulsion"[Title/Abstract])) OR ("Convulsive Seizures"[Title/Abstract])) OR ("Seizure, Convulsive"[Title/Abstract])) OR ("Seizures, Convulsive"[Title/Abstract])) OR ("Seizures, Motor"[Title/Abstract])) OR ("Motor Seizure"[Title/Abstract])) OR ("Motor Seizures"[Title/Abstract])) OR ("Seizure, Motor"[Title/Abstract])) OR ("Convulsive Seizure"[Title/Abstract])) OR ("Jacksonian Seizure"[Title/Abstract])) OR ("Seizure, Jacksonian"[Title/Abstract])) OR ("Seizures, Auditory"[Title/Abstract])) OR ("Auditory Seizure"[Title/Abstract])) OR ("Auditory Seizures"[Title/Abstract])) OR ("Seizure, Auditory"[Title/Abstract])) OR ("Seizures, Focal"[Title/Abstract])) OR ("Focal Seizure"[Title/Abstract])) OR ("Focal Seizures"[Title/Abstract])) OR ("Seizure, Focal"[Title/Abstract])) OR ("Partial Seizures"[Title/Abstract])) OR ("Partial Seizure"[Title/Abstract])) OR ("Seizure, Partial"[Title/Abstract])) OR ("Seizures, Generalized"[Title/Abstract])) OR ("Generalized Seizure"[Title/Abstract])) OR ("Generalized Seizures"[Title/Abstract])) OR ("Seizure, Generalized"[Title/Abstract])) OR ("Seizures, Gustatory"[Title/Abstract])) OR ("Gustatory Seizure"[Title/Abstract])) OR ("Gustatory Seizures"[Title/Abstract])) OR ("Seizure, Gustatory"[Title/Abstract])) OR ("Seizures, Olfactory"[Title/Abstract])) OR ("Olfactory Seizure"[Title/Abstract])) OR ("Olfactory Seizures"[Title/Abstract])) OR ("Seizure, Olfactory"[Title/Abstract])) OR ("Complex Partial Seizures"[Title/Abstract])) OR ("Complex Partial Seizure"[Title/Abstract])) OR ("Partial Seizure, Complex"[Title/Abstract])) OR ("Partial Seizures, Complex"[Title/Abstract])) OR ("Seizure, Complex Partial"[Title/Abstract])) OR ("Single Seizure"[Title/Abstract])) OR ("Seizure, Single"[Title/Abstract])) OR ("Single Seizures"[Title/Abstract])) OR ("Seizures, Somatosensory"[Title/Abstract])) OR ("Seizure, Somatosensory"[Title/Abstract])) OR ("Somatosensory Seizure"[Title/Abstract])) OR ("Somatosensory Seizures"[Title/Abstract])) OR ("Seizures, Vertiginous"[Title/Abstract])) OR ("Seizure, Vertiginous"[Title/Abstract])) OR ("Vertiginous Seizure"[Title/Abstract])) OR ("Vertiginous Seizures"[Title/Abstract])) OR ("Seizures, Vestibular"[Title/Abstract])) OR ("Seizure, Vestibular"[Title/Abstract])) OR ("Vestibular Seizure"[Title/Abstract])) OR ("Vestibular Seizures"[Title/Abstract])) OR ("Seizures, Visual"[Title/Abstract])) OR ("Seizure, Visual"[Title/Abstract])) OR ("Visual Seizure"[Title/Abstract])) OR ("Visual Seizures"[Title/Abstract])) OR ("Nonepileptic Seizures"[Title/Abstract])) OR ("Non-Epileptic Seizures"[Title/Abstract])) OR ("Non Epileptic Seizures"[Title/Abstract])) OR ("Nonepileptic Seizure"[Title/Abstract])) OR ("Seizure, Nonepileptic"[Title/Abstract])) OR ("Seizures, Nonepileptic"[Title/Abstract])) OR ("Non-Epileptic Seizure"[Title/Abstract])) OR ("Non Epileptic Seizure"[Title/Abstract])) OR ("Seizure, Non-Epileptic"[Title/Abstract])) OR ("Generalized Absence Seizures"[Title/Abstract])) OR ("Generalized Absence Seizure"[Title/Abstract])) OR ("Absence Seizure, Generalized"[Title/Abstract])) OR ("Absence Seizures, Generalized"[Title/Abstract])) OR ("Seizure, Generalized Absence"[Title/Abstract])) OR ("Tonic-Clonic Seizures"[Title/Abstract])) OR ("Tonic Clonic Seizure"[Title/Abstract])) OR ("Clonic Seizure, Tonic"[Title/Abstract])) OR ("Clonic Seizures, Tonic"[Title/Abstract])) OR ("Seizure, Tonic Clonic"[Title/Abstract])) OR ("Tonic Clonic Seizures"[Title/Abstract])) OR ("Generalized Tonic-Clonic Seizures"[Title/Abstract])) OR ("Generalized Tonic Clonic Seizures"[Title/Abstract])) OR ("Generalized Tonic-Clonic Seizure"[Title/Abstract])) OR ("Seizure, Generalized Tonic-Clonic"[Title/Abstract])) OR ("Seizures, Generalized Tonic-Clonic"[Title/Abstract])) OR ("Tonic-Clonic Seizure, Generalized"[Title/Abstract])) OR ("Tonic-Clonic Seizures, Generalized"[Title/Abstract])) OR ("Seizures, Tonic-Clonic"[Title/Abstract])) OR ("Tonic-Clonic Seizure"[Title/Abstract])) OR ("Seizure, Tonic-Clonic"[Title/Abstract])) OR ("Clonic Seizures"[Title/Abstract])) OR ("Clonic Seizure"[Title/Abstract])) OR ("Seizure, Clonic"[Title/Abstract])) OR ("Seizures, Clonic"[Title/Abstract])) OR ("Tonic Seizures"[Title/Abstract])) OR ("Seizures, Tonic"[Title/Abstract])) OR ("Tonic Seizure"[Title/Abstract])) OR ("Seizure, Tonic"[Title/Abstract])) OR ("Convulsion, Non-Epileptic"[Title/Abstract])) OR ("Convulsion, Non Epileptic"[Title/Abstract])) OR ("Convulsions, Non-Epileptic"[Title/Abstract])) OR ("Non-Epileptic Convulsion"[Title/Abstract])) OR ("Non-Epileptic Convulsions"[Title/Abstract])) OR ("Atonic Seizures"[Title/Abstract])) OR ("Atonic Seizure"[Title/Abstract])) OR ("Seizure, Atonic"[Title/Abstract])) OR ("Myoclonic Seizures"[Title/Abstract])) OR ("Myoclonic Seizure"[Title/Abstract])) OR ("Seizure, Myoclonic"[Title/Abstract])) OR ("Epileptic Seizures"[Title/Abstract])) OR ("Seizures, Epileptic"[Title/Abstract])) OR ("Epileptic Seizure"[Title/Abstract])) OR ("Seizure, Epileptic"[Title/Abstract]))) OR (((((((((((((((((((((((("Epilepsy, Absence"[Title/Abstract]) OR ("Absence Epilepsy"[Title/Abstract])) OR ("Epilepsy, Petit Mal"[Title/Abstract])) OR ("Petit Mal Epilepsy"[Title/Abstract])) OR ("Akinetic Petit Mal"[Title/Abstract])) OR ("Petit Mal, Akinetic"[Title/Abstract])) OR ("Childhood Absence Epilepsy"[Title/Abstract])) OR ("Absence Epilepsy, Childhood"[Title/Abstract])) OR ("Epilepsy, Childhood Absence"[Title/Abstract])) OR ("Pyknolepsy"[Title/Abstract])) OR ("Pyknolepsies"[Title/Abstract])) OR ("Pykno-Epilepsy"[Title/Abstract])) OR ("Pykno Epilepsy"[Title/Abstract])) OR ("Absence Seizure Disorder"[Title/Abstract])) OR ("Absence Seizure Disorders"[Title/Abstract])) OR ("Seizure Disorders, Absence"[Title/Abstract])) OR ("Seizure Disorder, Absence"[Title/Abstract])) OR ("Juvenile Absence Epilepsy"[Title/Abstract])) OR ("Absence Epilepsy, Juvenile"[Title/Abstract])) OR ("Epilepsy, Juvenile Absence"[Title/Abstract])) OR ("Epilepsy Juvenile Absence"[Title/Abstract])) OR ("Epilepsy, Absence, Atypical"[Title/Abstract])) OR ("Epilepsy, Minor"[Title/Abstract])) OR ("Minor Epilepsy"[Title/Abstract]))) AND (((((((("Stroke"[Mesh]) OR "Cerebral Infarction"[Mesh]) OR "Cerebral Hemorrhage"[Mesh]) OR "Brain Ischemia"[Mesh]) OR "Brain Infarction"[Mesh]) OR "Cerebrovascular Disorders"[Mesh]) OR (("Hemorrhagic Stroke"[Mesh]) OR "Ischemic Stroke"[Mesh])) OR ((((((((("Stroke"[Title/Abstract] OR "Strokes"[Title/Abstract] OR "Cerebrovascular Accident"[Title/Abstract] OR "Cerebrovascular Accidents"[Title/Abstract] OR "CVA (Cerebrovascular Accident)"[Title/Abstract] OR "CVAs (Cerebrovascular Accident)"[Title/Abstract] OR "Cerebrovascular Apoplexy"[Title/Abstract] OR "Apoplexy, Cerebrovascular"[Title/Abstract] OR "Vascular Accident, Brain"[Title/Abstract] OR "Brain Vascular Accident"[Title/Abstract] OR "Brain Vascular Accidents"[Title/Abstract] OR "Vascular Accidents, Brain"[Title/Abstract] OR "Cerebrovascular Stroke"[Title/Abstract] OR "Cerebrovascular Strokes"[Title/Abstract] OR "Stroke, Cerebrovascular"[Title/Abstract] OR "Strokes, Cerebrovascular"[Title/Abstract] OR "Apoplexy"[Title/Abstract] OR "Cerebral Stroke"[Title/Abstract] OR "Cerebral Strokes"[Title/Abstract] OR "Stroke, Cerebral"[Title/Abstract] OR "Strokes, Cerebral"[Title/Abstract] OR "Stroke, Acute"[Title/Abstract] OR "Acute Stroke"[Title/Abstract] OR "Acute Strokes"[Title/Abstract] OR "Strokes, Acute"[Title/Abstract] OR "Cerebrovascular Accident, Acute"[Title/Abstract] OR "Acute Cerebrovascular Accident"[Title/Abstract] OR "Acute Cerebrovascular Accidents"[Title/Abstract] OR "Cerebrovascular Accidents, Acute"[Title/Abstract]) OR ("Cerebral Infarction"[Title/Abstract] OR "Cerebral Infarctions"[Title/Abstract] OR "Infarctions, Cerebral"[Title/Abstract] OR "Infarction, Cerebral"[Title/Abstract] OR "Cerebral Infarct"[Title/Abstract] OR "Cerebral Infarcts"[Title/Abstract] OR "Infarct, Cerebral"[Title/Abstract] OR "Infarcts, Cerebral"[Title/Abstract] OR "Cerebral Infarction, Left Hemisphere"[Title/Abstract] OR "Left Hemisphere, Cerebral Infarction"[Title/Abstract] OR "Infarction, Cerebral, Left Hemisphere"[Title/Abstract] OR "Cerebral, Left Hemisphere, Infarction"[Title/Abstract] OR "Infarction, Left Hemisphere, Cerebral"[Title/Abstract] OR "Left Hemisphere, Infarction, Cerebral"[Title/Abstract] OR "Subcortical Infarction"[Title/Abstract] OR "Infarction, Subcortical"[Title/Abstract] OR "Infarctions, Subcortical"[Title/Abstract] OR "Subcortical Infarctions"[Title/Abstract] OR "Posterior Choroidal Artery Infarction"[Title/Abstract] OR "Anterior Choroidal Artery Infarction"[Title/Abstract] OR "Cerebral Infarction, Right Hemisphere"[Title/Abstract] OR "Right Hemisphere, Cerebral Infarction"[Title/Abstract] OR "Infarction, Right Hemisphere, Cerebral"[Title/Abstract] OR "Right Hemisphere, Infarction, Cerebral"[Title/Abstract] OR "Cerebral, Right Hemisphere, Infarction"[Title/Abstract] OR "Infarction, Cerebral, Right Hemisphere"[Title/Abstract])) OR ("Cerebral Hemorrhage"[Title/Abstract] OR "Hemorrhage, Cerebrum"[Title/Abstract] OR "Cerebrum Hemorrhage"[Title/Abstract] OR "Cerebrum Hemorrhages"[Title/Abstract] OR "Hemorrhages, Cerebrum"[Title/Abstract] OR "Cerebral Parenchymal Hemorrhage"[Title/Abstract] OR "Cerebral Parenchymal Hemorrhages"[Title/Abstract] OR "Hemorrhage, Cerebral Parenchymal"[Title/Abstract] OR "Hemorrhages, Cerebral Parenchymal"[Title/Abstract] OR "Parenchymal Hemorrhage, Cerebral"[Title/Abstract] OR "Parenchymal Hemorrhages, Cerebral"[Title/Abstract] OR "Intracerebral Hemorrhage"[Title/Abstract] OR "Hemorrhage, Intracerebral"[Title/Abstract] OR "Hemorrhages, Intracerebral"[Title/Abstract] OR "Intracerebral Hemorrhages"[Title/Abstract] OR "Hemorrhage, Cerebral"[Title/Abstract] OR "Cerebral Hemorrhages"[Title/Abstract] OR "Hemorrhages, Cerebral"[Title/Abstract] OR "Brain Hemorrhage, Cerebral"[Title/Abstract] OR "Brain Hemorrhages, Cerebral"[Title/Abstract] OR "Cerebral Brain Hemorrhage"[Title/Abstract] OR "Cerebral Brain Hemorrhages"[Title/Abstract] OR "Hemorrhage, Cerebral Brain"[Title/Abstract] OR "Hemorrhages, Cerebral Brain"[Title/Abstract])) OR ("Hemorrhagic Stroke"[Title/Abstract] OR "Hemorrhagic Strokes"[Title/Abstract] OR "Stroke, Hemorrhagic"[Title/Abstract] OR "Subarachnoid Hemorrhagic Stroke"[Title/Abstract] OR "Hemorrhagic Stroke, Subarachnoid"[Title/Abstract] OR "Stroke, Subarachnoid Hemorrhagic"[Title/Abstract] OR "Subarachnoid Hemorrhagic Strokes"[Title/Abstract] OR "Intracerebral Hemorrhagic Stroke"[Title/Abstract] OR "Hemorrhagic Stroke, Intracerebral"[Title/Abstract] OR "Intracerebral Hemorrhagic Strokes"[Title/Abstract] OR "Stroke, Intracerebral Hemorrhagic"[Title/Abstract] OR "Intracerebral Hemorrhage Stroke"[Title/Abstract] OR "Hemorrhage Stroke, Intracerebral"[Title/Abstract] OR "Intracerebral Hemorrhage Strokes"[Title/Abstract] OR "Stroke, Intracerebral Hemorrhage"[Title/Abstract])) OR ("Ischemic Stroke"[Title/Abstract] OR "Ischemic Strokes"[Title/Abstract] OR "Stroke, Ischemic"[Title/Abstract] OR "Ischaemic Stroke"[Title/Abstract] OR "Ischaemic Strokes"[Title/Abstract] OR "Stroke, Ischaemic"[Title/Abstract] OR "Cryptogenic Ischemic Stroke"[Title/Abstract] OR "Cryptogenic Ischemic Strokes"[Title/Abstract] OR "Ischemic Stroke, Cryptogenic"[Title/Abstract] OR "Stroke, Cryptogenic Ischemic"[Title/Abstract] OR "Cryptogenic Stroke"[Title/Abstract] OR "Cryptogenic Strokes"[Title/Abstract] OR "Stroke, Cryptogenic"[Title/Abstract] OR "Cryptogenic Embolism Stroke"[Title/Abstract] OR "Cryptogenic Embolism Strokes"[Title/Abstract] OR "Embolism Stroke, Cryptogenic"[Title/Abstract] OR "Stroke, Cryptogenic Embolism"[Title/Abstract] OR "Wake-up Stroke"[Title/Abstract] OR "Stroke, Wake-up"[Title/Abstract] OR "Wake up Stroke"[Title/Abstract] OR "Wake-up Strokes"[Title/Abstract] OR "Acute Ischemic Stroke"[Title/Abstract] OR "Acute Ischemic Strokes"[Title/Abstract] OR "Ischemic Stroke, Acute"[Title/Abstract] OR "Stroke, Acute Ischemic"[Title/Abstract])) OR ("Brain ischemia"[Title/Abstract] OR "Brain Ischemias"[Title/Abstract] OR "Ischemia, Brain"[Title/Abstract] OR "Ischemic Encephalopathy"[Title/Abstract] OR "Encephalopathy, Ischemic"[Title/Abstract] OR "Ischemic Encephalopathies"[Title/Abstract] OR "Cerebral Ischemia"[Title/Abstract] OR "Cerebral Ischemias"[Title/Abstract] OR "Ischemias, Cerebral"[Title/Abstract] OR "Ischemia, Cerebral"[Title/Abstract])) OR ("Brain Infarction"[Title/Abstract] OR "Brain Infarctions"[Title/Abstract] OR "Infarction, Brain"[Title/Abstract] OR "Infarctions, Brain"[Title/Abstract] OR "Brain Infarct"[Title/Abstract] OR "Brain Infarcts"[Title/Abstract] OR "Infarct, Brain"[Title/Abstract] OR "Infarcts, Brain"[Title/Abstract] OR "Brain Infarction, Posterior Circulation"[Title/Abstract] OR "Infarction, Posterior Circulation, Brain"[Title/Abstract] OR "Posterior Circulation Infarction, Brain"[Title/Abstract] OR "Infarction, Brain, Posterior Circulation"[Title/Abstract] OR "Posterior Circulation Brain Infarction"[Title/Abstract] OR "Anterior Circulation Brain Infarction"[Title/Abstract] OR "Infarction, Brain, Anterior Circulation"[Title/Abstract] OR "Brain Infarction, Anterior Circulation"[Title/Abstract] OR "Anterior Circulation Infarction, Brain"[Title/Abstract] OR "Infarction, Anterior Circulation, Brain"[Title/Abstract] OR "Venous Infarction, Brain"[Title/Abstract] OR "Brain Venous Infarction"[Title/Abstract] OR "Brain Venous Infarctions"[Title/Abstract] OR "Infarction, Brain Venous"[Title/Abstract] OR "Infarctions, Brain Venous"[Title/Abstract] OR "Venous Infarctions, Brain"[Title/Abstract] OR "Brain Infarction, Venous"[Title/Abstract] OR "Brain Infarctions, Venous"[Title/Abstract] OR "Infarction, Venous Brain"[Title/Abstract] OR "Infarctions, Venous Brain"[Title/Abstract] OR "Venous Brain Infarction"[Title/Abstract] OR "Venous Brain Infarctions"[Title/Abstract] OR "Anterior Cerebral Circulation Infarction"[Title/Abstract] OR "Infarction, Anterior Cerebral Circulation"[Title/Abstract])) OR ("Cerebrovascular Disorders"[Title/Abstract] OR "Cerebrovascular Disorder"[Title/Abstract] OR "Cerebrovascular Diseases"[Title/Abstract] OR "Cerebrovascular Disease"[Title/Abstract] OR "Disease, Cerebrovascular"[Title/Abstract] OR "Diseases, Cerebrovascular"[Title/Abstract] OR "Vascular Diseases, Intracranial"[Title/Abstract] OR "Intracranial Vascular Disease"[Title/Abstract] OR "Intracranial Vascular Diseases"[Title/Abstract] OR "Vascular Disease, Intracranial"[Title/Abstract] OR "Brain Vascular Disorders"[Title/Abstract] OR "Brain Vascular Disorder"[Title/Abstract] OR "Vascular Disorder, Brain"[Title/Abstract] OR "Vascular Disorders, Brain"[Title/Abstract] OR "Intracranial Vascular Disorders"[Title/Abstract] OR "Intracranial Vascular Disorder"[Title/Abstract] OR "Vascular Disorder, Intracranial"[Title/Abstract] OR "Vascular Disorders, Intracranial"[Title/Abstract] OR "Cerebrovascular Insufficiency"[Title/Abstract] OR "Cerebrovascular Insufficiencies"[Title/Abstract] OR "Insufficiencies, Cerebrovascular"[Title/Abstract] OR "Insufficiency, Cerebrovascular"[Title/Abstract] OR "Cerebrovascular Occlusion"[Title/Abstract] OR "Cerebrovascular Occlusions"[Title/Abstract] OR "Occlusion, Cerebrovascular"[Title/Abstract] OR "Occlusions, Cerebrovascular"[Title/Abstract])) OR ("CVA"[Title/Abstract] OR "Intracranial Thromb*"[Title/Abstract] OR "Ischemic Event "[Title/Abstract] OR "Brain Thrombosis"[Title/Abstract] OR "Cerebral Thrombus"[Title/Abstract] OR "Ischemic Encephalopathies"[Title/Abstract] OR "Ischemic Attack"[Title/Abstract])))) OR (("Post stroke Epilepsy"[Title/Abstract]) OR ("Poststroke Epilepsy"[Title/Abstract]))) AND ((((("Herbal Medicine"[Mesh]) OR "Medicine, Chinese Traditional"[Mesh]) OR "Drugs, Chinese Herbal"[Mesh]) OR "Medicine, Traditional"[Mesh]) OR ("Herbal Medicine"[Title/Abstract] OR "Medicine, Herbal"[Title/Abstract] OR "Hawaiian Herbal Medicine*"[Title/Abstract] OR "Herbal Medicine*, Hawaiian"[Title/Abstract] OR "Medicine*, Hawaiian Herbal"[Title/Abstract] OR "La'au Lapa'au"[Title/Abstract] OR "Laau Lapaau"[Title/Abstract] OR "La au Lapa au"[Title/Abstract] OR "Herbalism"[Title/Abstract] OR "Zhong Yi Xue"[Title/Abstract] OR "Chung I Hsueh"[Title/Abstract] OR "Hsueh, Chung I"[Title/Abstract] OR "Chinese Medicine, Traditional"[Title/Abstract] OR "Chinese Traditional Medicine"[Title/Abstract] OR "Traditional Chinese Medicine"[Title/Abstract] OR "Traditional Medicine, Chinese"[Title/Abstract] OR "Traditional Tongue Diagnosis"[Title/Abstract] OR "Tongue Diagnoses, Traditional"[Title/Abstract] OR "Tongue Diagnosis, Traditional"[Title/Abstract] OR "Traditional Tongue Diagnoses"[Title/Abstract] OR "Traditional Tongue Assessment"[Title/Abstract] OR "Tongue Assessment, Traditional"[Title/Abstract] OR "Traditional Tongue Assessments"[Title/Abstract] OR "Drugs, Chinese Herbal"[Title/Abstract] OR "Chinese Drugs, Plant"[Title/Abstract] OR "Chinese Herbal Drugs"[Title/Abstract] OR "Herbal Drugs, Chinese"[Title/Abstract] OR "Plant Extracts, Chinese"[Title/Abstract] OR "Chinese Plant Extracts"[Title/Abstract] OR "Extracts, Chinese Plant"[Title/Abstract] OR "Medicine, Traditional"[Title/Abstract] OR "Traditional Medicine"[Title/Abstract] OR "Home Remed*"[Title/Abstract] OR "Remed*, Home"[Title/Abstract] OR "Medicine, Primitive"[Title/Abstract] OR "Primitive Medicine"[Title/Abstract] OR "Medicine, Folk"[Title/Abstract] OR "Folk Medicine"[Title/Abstract] OR "Medicine, Indigenous"[Title/Abstract] OR "Indigenous Medicine"[Title/Abstract] OR "Folk Remed*"[Title/Abstract] OR "Remed*, Folk"[Title/Abstract] OR "Ethnomedicine"[Title/Abstract] OR "Chinese medicine"[Title/Abstract] OR "TCM"[Title/Abstract] OR "integrated chinese and western medicine "[Title/Abstract] OR "decoction"[Title/Abstract] OR "chinese patent medicine"[Title/Abstract] OR "Chinese herbal medicine"[Title/Abstract] OR "oriental medicine"[Title/Abstract] OR "herbal formula"[Title/Abstract] OR "herb*"[Title/Abstract] OR "Alternative Medicine*"[Title/Abstract] OR "Alternative Therap*"[Title/Abstract] OR "Complementary Medicine*"[Title/Abstract] OR "Complementary Therap*"[Title/Abstract] OR "Integrat* Medicine*"[Title/Abstract] OR "Integrat* Therap*"[Title/Abstract]))) AND (("Randomized Controlled Trial" [Publication Type] OR "Randomized Controlled Trials as Topic"[Mesh] OR "Controlled Clinical Trial" [Publication Type]) OR ((((((((((((((((((("Randomized Controlled Trial"[Title/Abstract]) OR ("Randomized Controlled Trials as Topic"[Title/Abstract])) OR ("Controlled Clinical Trial"[Title/Abstract])) OR ("Randomized"[Title/Abstract])) OR ("placebo"[Title/Abstract])) OR ("randomly"[Title/Abstract])) OR ("trial"[Title/Abstract])) OR ("groups"[Title/Abstract])) OR ("Clinical Trials"[Title/Abstract])) OR ("Random"[Title/Abstract])) OR ("RCT"[Title/Abstract])) OR ("randomized controlled study"[Title/Abstract])) OR ("Controlled Clinical study"[Title/Abstract])) OR ("randomized trial"[Title/Abstract])) OR ("randomized study"[Title/Abstract])) OR ("randomized placebo-controlled study"[Title/Abstract])) OR ("randomized parallel-group study"[Title/Abstract])) OR ("randomized placebo controlled"[Title/Abstract])) OR ("randomized double-blin*"[Title/Abstract])))

n=26

**Embase**

SourcesEmbase, MEDLINE, Preprints

Query(('epilepsy'/exp OR 'seizure'/exp OR 'absence'/exp OR 'convulsion'/exp OR 'epilepsy':ab,ti,kw OR 'audiogenic seizure':ab,ti,kw OR 'catamenial epilepsy':ab,ti,kw OR 'drug resistant epilepsy':ab,ti,kw OR 'epileptic aura':ab,ti,kw OR 'epileptic discharge':ab,ti,kw OR 'epileptic focus':ab,ti,kw OR 'epileptic state':ab,ti,kw OR 'experimental epilepsy':ab,ti,kw OR 'focal epilepsy':ab,ti,kw OR 'generalized epilepsy':ab,ti,kw OR 'intractable epilepsy':ab,ti,kw OR 'matutinal seizure':ab,ti,kw OR 'migralepsy':ab,ti,kw OR 'mixed seizure':ab,ti,kw OR 'mtoropathy':ab,ti,kw OR 'neuronal ceroid lipofuscinosis':ab,ti,kw OR 'nocturnal seizure':ab,ti,kw OR 'photosensitive epilepsy':ab,ti,kw OR 'reflex epilepsy':ab,ti,kw OR 'severe myoclonic epilepsy in infancy':ab,ti,kw OR 'startle epilepsy':ab,ti,kw OR 'sudden unexpected death in epilepsy':ab,ti,kw OR 'symptomatic epilepsy':ab,ti,kw OR 'traumatic epilepsy':ab,ti,kw OR 'seizure':ab,ti,kw OR 'absence seizure':ab,ti,kw OR 'experimental seizure':ab,ti,kw OR 'hypoglycemic seizure':ab,ti,kw OR 'withdrawal seizure':ab,ti,kw OR 'absence':ab,ti,kw OR 'convulsion':ab,ti,kw) AND ('cerebrovascular accident'/exp OR 'brain infarction'/exp OR 'brain hemorrhage'/exp OR 'brain ischemia'/exp OR 'ischemic stroke'/exp OR 'occlusive cerebrovascular disease'/exp OR 'cerebrovascular accident':ab,ti,kw OR 'brainstem stroke':ab,ti,kw OR 'cardioembolic stroke':ab,ti,kw OR 'experimental stroke':ab,ti,kw OR 'lacunar stroke':ab,ti,kw OR 'brain infarction':ab,ti,kw OR 'anterior circulation infarction':ab,ti,kw OR 'brain infarction size':ab,ti,kw OR 'brain stem infarction':ab,ti,kw OR 'cadasil':ab,ti,kw OR 'cerebellum infarction':ab,ti,kw OR 'lacunar infarction':ab,ti,kw OR 'migrainous infarction':ab,ti,kw OR 'multiinfarct dementia':ab,ti,kw OR 'posterior circulation infarction':ab,ti,kw OR 'brain hemorrhage':ab,ti,kw OR 'brain ventricle hemorrhage':ab,ti,kw OR 'cerebellum hemorrhage':ab,ti,kw OR 'massive intracerebral hemorrhage':ab,ti,kw OR 'subarachnoid hemorrhage':ab,ti,kw OR 'brain ischemia':ab,ti,kw OR 'anterior circulation ischemia':ab,ti,kw OR 'brain vasospasm':ab,ti,kw OR 'experimental cerebral ischemia':ab,ti,kw OR 'hypoxic ischemic encephalopathy':ab,ti,kw OR 'posterior circulation ischemia':ab,ti,kw OR 'transient ischemic attack':ab,ti,kw OR 'ischemic stroke':ab,ti,kw OR 'acute ischemic stroke':ab,ti,kw OR 'anterior circulation stroke':ab,ti,kw OR 'chronic ischemic stroke':ab,ti,kw OR 'cryptogenic ischemic stroke':ab,ti,kw OR 'posterior circulation stroke':ab,ti,kw OR 'subacute ischemic stroke':ab,ti,kw OR 'wake up stroke':ab,ti,kw OR 'occlusive cerebrovascular disease':ab,ti,kw OR 'basilar artery obstruction':ab,ti,kw OR 'cerebral sinus thrombosis':ab,ti,kw OR 'middle cerebral artery occlusion':ab,ti,kw OR 'sneddon syndrome':ab,ti,kw OR 'susac syndrome':ab,ti,kw OR 'vertebral artery stenosis':ab,ti,kw OR 'intracranial thromb*':ab,ti,kw OR 'ischemic event':ab,ti,kw OR 'brain thrombosis':ab,ti,kw OR 'cerebral thrombus':ab,ti,kw OR 'ischemic encephalopathies':ab,ti,kw OR 'ischemic attack':ab,ti,kw) OR 'poststroke epilepsy'/exp OR 'poststroke epilepsy':ab,ti,kw OR 'post-stroke epilepsy':ab,ti,kw) AND ('herbal medicine'/exp OR 'traditional medicine'/exp OR 'chinese medicine'/exp OR 'chinese drug'/exp OR 'chinese medicinal formula'/exp OR 'chinese patent medicine'/exp OR 'oriental medicine'/exp OR 'complementary therapy'/exp OR 'alternative medicine'/exp OR 'medicinal plant'/exp OR 'herbaceous agent'/exp OR 'kampo medicine'/exp OR 'kampo medicine (drug)'/exp OR 'herb'/exp OR 'plant medicinal product'/exp OR 'plant'/exp OR 'phytotherapy'/exp OR 'herbal medicine':ab,ti,kw OR 'traditional medicine':ab,ti,kw OR 'chinese medicine':ab,ti,kw OR 'chinese drug':ab,ti,kw OR 'chinese medicinal formula':ab,ti,kw OR 'chinese patent medicine':ab,ti,kw OR 'oriental medicine':ab,ti,kw OR 'complementary therapy':ab,ti,kw OR 'alternative medicine':ab,ti,kw OR 'medicinal plant':ab,ti,kw OR 'herbaceous agent':ab,ti,kw OR 'kampo medicine':ab,ti,kw OR 'kampo medicine (drug)':ab,ti,kw OR 'herb':ab,ti,kw OR 'plant medicinal product':ab,ti,kw OR 'plant':ab,ti,kw OR 'phytotherapy':ab,ti,kw OR 'tcm':ab,ti,kw OR 'integrated chinese and western medicine':ab,ti,kw OR 'herbalism':ab,ti,kw OR 'injection':ab,ti,kw OR 'decoction':ab,ti,kw OR 'herb*':ab,ti,kw OR 'chinese herbal medicine':ab,ti,kw OR 'herbal formula':ab,ti,kw OR 'alternative therap*':ab,ti,kw OR 'complementary medicine*':ab,ti,kw OR 'complementary therap*':ab,ti,kw OR 'integrat* medicine*':ab,ti,kw OR 'integrat* therap*':ab,ti,kw) AND ('randomized controlled trial'/exp OR 'randomized controlled trial (topic)'/exp OR 'controlled clinical trial'/exp OR 'randomized controlled trial':ab,ti,kw OR 'randomized controlled trial (topic)':ab,ti,kw OR 'controlled clinical trial':ab,ti,kw OR 'randomized':ab,ti,kw OR 'placebo':ab,ti,kw OR 'randomly':ab,ti,kw OR 'trial':ab,ti,kw OR 'groups':ab,ti,kw OR 'clinical trials':ab,ti,kw OR 'random':ab,ti,kw OR 'rct':ab,ti,kw OR 'randomized controlled study':ab,ti,kw OR 'randomized trial':ab,ti,kw OR 'randomized study':ab,ti,kw OR 'randomized placebo-controlled study':ab,ti,kw OR 'randomized parallel-group study':ab,ti,kw OR 'randomized placebo controlled':ab,ti,kw OR 'randomized double-blind':ab,ti,kw)

Mapped termsn/a

n=826

**Cochrane Library**

Date Run: 11/10/2023 22:47:23

ID Search Hits

#1 MeSH descriptor: [Epilepsy] explode all trees 3464

#2 MeSH descriptor: [Seizures] explode all trees 1514

#3 MeSH descriptor: [Epilepsy, Absence] explode all trees 58

#4 MeSH descriptor: [Convulsants] explode all trees 6

#5 #1 OR #2 OR #3 OR #4 4478

#6 (“Epilepsy”):ti,ab,kw OR (“Epilepsies”):ti,ab,kw OR (“Seizure Disorder”):ti,ab,kw OR (“Seizure Disorders”):ti,ab,kw OR (“Awakening Epilepsy”):ti,ab,kw OR (“Epilepsy, Awakening”):ti,ab,kw OR (“Epilepsy, Cryptogenic”):ti,ab,kw OR (“Cryptogenic Epilepsies”):ti,ab,kw OR (“Cryptogenic Epilepsy”):ti,ab,kw OR (“Epilepsies, Cryptogenic”):ti,ab,kw OR (“Aura”):ti,ab,kw OR (“Auras”):ti,ab,kw OR (“seizures”):ti,ab,kw OR (“Seizure”):ti,ab,kw OR (“Atonic Absence Seizures”):ti,ab,kw OR (“Atonic Absence Seizure”):ti,ab,kw OR (“Absence Seizure, Atonic”):ti,ab,kw OR (“Absence Seizures, Atonic”):ti,ab,kw OR (“Seizure, Atonic Absence”):ti,ab,kw OR (“Seizures, Sensory”):ti,ab,kw OR (“Seizure, Sensory”):ti,ab,kw OR (“Sensory Seizure”):ti,ab,kw OR (“Sensory Seizures”):ti,ab,kw OR (“Absence Seizures”):ti,ab,kw OR (“Petit Mal Convulsion”):ti,ab,kw OR (“Convulsion, Petit Mal”):ti,ab,kw OR (“Absence Seizure”):ti,ab,kw OR (“Seizure, Absence”):ti,ab,kw OR (“Convulsions”):ti,ab,kw OR (“Convulsion”):ti,ab,kw OR (“Convulsive Seizures”):ti,ab,kw OR (“Seizure, Convulsive”):ti,ab,kw OR (“Seizures, Convulsive”):ti,ab,kw OR (“Seizures, Motor”):ti,ab,kw OR (“Motor Seizure”):ti,ab,kw OR (“Motor Seizures”):ti,ab,kw OR (“Seizure, Motor”):ti,ab,kw OR (“Convulsive Seizure”):ti,ab,kw OR (“Jacksonian Seizure”):ti,ab,kw OR (“Seizure, Jacksonian”):ti,ab,kw OR (“Seizures, Auditory”):ti,ab,kw OR (“Auditory Seizure”):ti,ab,kw OR (“Auditory Seizures”):ti,ab,kw OR (“Seizure, Auditory”):ti,ab,kw OR (“Seizures, Focal”):ti,ab,kw OR (“Focal Seizure”):ti,ab,kw OR (“Focal Seizures”):ti,ab,kw OR (“Seizure, Focal”):ti,ab,kw OR (“Partial Seizures”):ti,ab,kw OR (“Partial Seizure”):ti,ab,kw OR (“Seizure, Partial”):ti,ab,kw OR (“Seizures, Generalized”):ti,ab,kw OR (“Generalized Seizure”):ti,ab,kw OR (“Generalized Seizures”):ti,ab,kw OR (“Seizure, Generalized”):ti,ab,kw OR (“Seizures, Gustatory”):ti,ab,kw OR (“Gustatory Seizure”):ti,ab,kw OR (“Gustatory Seizures”):ti,ab,kw OR (“Seizure, Gustatory”):ti,ab,kw OR (“Seizures, Olfactory”):ti,ab,kw OR (“Olfactory Seizure”):ti,ab,kw OR (“Olfactory Seizures”):ti,ab,kw OR (“Seizure, Olfactory”):ti,ab,kw OR (“Complex Partial Seizures”):ti,ab,kw OR (“Complex Partial Seizure”):ti,ab,kw OR (“Partial Seizure, Complex”):ti,ab,kw OR (“Partial Seizures, Complex”):ti,ab,kw OR (“Seizure, Complex Partial”):ti,ab,kw OR (“Single Seizure”):ti,ab,kw OR (“Seizure, Single”):ti,ab,kw OR (“Single Seizures”):ti,ab,kw OR (“Seizures, Somatosensory”):ti,ab,kw OR (“Seizure, Somatosensory”):ti,ab,kw OR (“Somatosensory Seizure”):ti,ab,kw OR (“Somatosensory Seizures”):ti,ab,kw OR (“Seizures, Vertiginous”):ti,ab,kw OR (“Seizure, Vertiginous”):ti,ab,kw OR (“Vertiginous Seizure”):ti,ab,kw OR (“Vertiginous Seizures”):ti,ab,kw OR (“Seizures, Vestibular”):ti,ab,kw OR (“Seizure, Vestibular”):ti,ab,kw OR (“Vestibular Seizure”):ti,ab,kw OR (“Vestibular Seizures”):ti,ab,kw OR (“Seizures, Visual”):ti,ab,kw OR (“Seizure, Visual”):ti,ab,kw OR (“Visual Seizure”):ti,ab,kw OR (“Visual Seizures”):ti,ab,kw OR (“Nonepileptic Seizures”):ti,ab,kw OR (“Non-Epileptic Seizures”):ti,ab,kw OR (“Non Epileptic Seizures”):ti,ab,kw OR (“Nonepileptic Seizure”):ti,ab,kw OR (“Seizure, Nonepileptic”):ti,ab,kw OR (“Seizures, Nonepileptic”):ti,ab,kw OR (“Non-Epileptic Seizure”):ti,ab,kw OR (“Non Epileptic Seizure”):ti,ab,kw OR (“Seizure, Non-Epileptic”):ti,ab,kw OR (“Generalized Absence Seizures”):ti,ab,kw OR (“Generalized Absence Seizure”):ti,ab,kw OR (“Absence Seizure, Generalized”):ti,ab,kw OR (“Absence Seizures, Generalized”):ti,ab,kw OR (“Seizure, Generalized Absence”):ti,ab,kw OR (“Tonic-Clonic Seizures”):ti,ab,kw OR (“Tonic Clonic Seizure”):ti,ab,kw OR (“Clonic Seizure, Tonic”):ti,ab,kw OR (“Clonic Seizures, Tonic”):ti,ab,kw OR (“Seizure, Tonic Clonic”):ti,ab,kw OR (“Tonic Clonic Seizures”):ti,ab,kw OR (“Generalized Tonic-Clonic Seizures”):ti,ab,kw OR (“Generalized Tonic Clonic Seizures”):ti,ab,kw OR (“Generalized Tonic-Clonic Seizure”):ti,ab,kw OR (“Seizure, Generalized Tonic-Clonic”):ti,ab,kw OR (“Seizures, Generalized Tonic-Clonic”):ti,ab,kw OR (“Tonic-Clonic Seizure, Generalized”):ti,ab,kw OR (“Tonic-Clonic Seizures, Generalized”):ti,ab,kw OR (“Seizures, Tonic-Clonic”):ti,ab,kw OR (“Tonic-Clonic Seizure”):ti,ab,kw OR (“Seizure, Tonic-Clonic”):ti,ab,kw OR (“Clonic Seizures”):ti,ab,kw OR (“Clonic Seizure”):ti,ab,kw OR (“Seizure, Clonic”):ti,ab,kw OR (“Seizures, Clonic”):ti,ab,kw OR (“Tonic Seizures”):ti,ab,kw OR (“Seizures, Tonic”):ti,ab,kw OR (“Tonic Seizure”):ti,ab,kw OR (“Seizure, Tonic”):ti,ab,kw OR (“Convulsion, Non-Epileptic”):ti,ab,kw OR (“Convulsion, Non Epileptic”):ti,ab,kw OR (“Convulsions, Non-Epileptic”):ti,ab,kw OR (“Non-Epileptic Convulsion”):ti,ab,kw OR (“Non-Epileptic Convulsions”):ti,ab,kw OR (“Atonic Seizures”):ti,ab,kw OR (“Atonic Seizure”):ti,ab,kw OR (“Seizure, Atonic”):ti,ab,kw OR (“Myoclonic Seizures”):ti,ab,kw OR (“Myoclonic Seizure”):ti,ab,kw OR (“Seizure, Myoclonic”):ti,ab,kw OR (“Epileptic Seizures”):ti,ab,kw OR (“Seizures, Epileptic”):ti,ab,kw OR (“Epileptic Seizure”):ti,ab,kw OR (“Seizure, Epileptic”):ti,ab,kw OR (“Epilepsy, Absence”):ti,ab,kw OR (“Absence Epilepsy”):ti,ab,kw OR (“Epilepsy, Petit Mal”):ti,ab,kw OR (“Petit Mal Epilepsy”):ti,ab,kw OR (“Akinetic Petit Mal”):ti,ab,kw OR (“Petit Mal, Akinetic”):ti,ab,kw OR (“Childhood Absence Epilepsy”):ti,ab,kw OR (“Absence Epilepsy, Childhood”):ti,ab,kw OR (“Epilepsy, Childhood Absence”):ti,ab,kw OR (“Pyknolepsy”):ti,ab,kw OR (“Pyknolepsies”):ti,ab,kw OR (“Pykno-Epilepsy”):ti,ab,kw OR (“Pykno Epilepsy”):ti,ab,kw OR (“Absence Seizure Disorder”):ti,ab,kw OR (“Absence Seizure Disorders”):ti,ab,kw OR (“Seizure Disorders, Absence”):ti,ab,kw OR (“Seizure Disorder, Absence”):ti,ab,kw OR (“Juvenile Absence Epilepsy”):ti,ab,kw OR (“Absence Epilepsy, Juvenile”):ti,ab,kw OR (“Epilepsy, Juvenile Absence”):ti,ab,kw OR (“Epilepsy Juvenile Absence”):ti,ab,kw OR (“Epilepsy, Absence, Atypical”):ti,ab,kw OR (“Epilepsy, Minor”):ti,ab,kw OR (“Minor Epilepsy”):ti,ab,kw OR (“Convulsion”):ti,ab,kw 16729

#7 #5 OR #6 16826

#8 MeSH descriptor: [Stroke] explode all trees 15152

#9 MeSH descriptor: [Cerebral Infarction] explode all trees 1502

#10 MeSH descriptor: [Cerebral Hemorrhage] explode all trees 1392

#11 MeSH descriptor: [Brain Ischemia] explode all trees 5995

#12 MeSH descriptor: [Brain Infarction] explode all trees 1947

#13 MeSH descriptor: [Cerebrovascular Disorders] explode all trees 22881

#14 MeSH descriptor: [Hemorrhagic Stroke] explode all trees 40

#15 MeSH descriptor: [Ischemic Stroke] explode all trees 994

#16 #8 OR #9 OR #10 OR #11 OR #12 OR #13 OR #14 OR #15 22881

#17 ("Stroke"):ti,ab,kw OR ("Strokes"):ti,ab,kw OR ("Cerebrovascular Accident"):ti,ab,kw OR ("Cerebrovascular Accidents"):ti,ab,kw OR ("CVA (Cerebrovascular Accident)"):ti,ab,kw OR ("CVAs (Cerebrovascular Accident)"):ti,ab,kw OR ("Cerebrovascular Apoplexy"):ti,ab,kw OR ("Apoplexy, Cerebrovascular"):ti,ab,kw OR ("Vascular Accident, Brain"):ti,ab,kw OR ("Brain Vascular Accident"):ti,ab,kw OR ("Brain Vascular Accidents"):ti,ab,kw OR ("Vascular Accidents, Brain"):ti,ab,kw OR ("Cerebrovascular Stroke"):ti,ab,kw OR ("Cerebrovascular Strokes"):ti,ab,kw OR ("Stroke, Cerebrovascular"):ti,ab,kw OR ("Strokes, Cerebrovascular"):ti,ab,kw OR ("Apoplexy"):ti,ab,kw OR ("Cerebral Stroke"):ti,ab,kw OR ("Cerebral Strokes"):ti,ab,kw OR ("Stroke, Cerebral"):ti,ab,kw OR ("Strokes, Cerebral"):ti,ab,kw OR ("Stroke, Acute"):ti,ab,kw OR ("Acute Stroke"):ti,ab,kw OR ("Acute Strokes"):ti,ab,kw OR ("Strokes, Acute"):ti,ab,kw OR ("Cerebrovascular Accident, Acute"):ti,ab,kw OR ("Acute Cerebrovascular Accident"):ti,ab,kw OR ("Acute Cerebrovascular Accidents"):ti,ab,kw OR ("Cerebrovascular Accidents, Acute"):ti,ab,kw OR ("Cerebral Infarction"):ti,ab,kw OR ("Cerebral Infarctions"):ti,ab,kw OR ("Infarctions, Cerebral"):ti,ab,kw OR ("Infarction, Cerebral"):ti,ab,kw OR ("Cerebral Infarct"):ti,ab,kw OR ("Cerebral Infarcts"):ti,ab,kw OR ("Infarct, Cerebral"):ti,ab,kw OR ("Infarcts, Cerebral"):ti,ab,kw OR ("Cerebral Infarction, Left Hemisphere"):ti,ab,kw OR ("Left Hemisphere, Cerebral Infarction"):ti,ab,kw OR ("Infarction, Cerebral, Left Hemisphere"):ti,ab,kw OR ("Cerebral, Left Hemisphere, Infarction"):ti,ab,kw OR ("Infarction, Left Hemisphere, Cerebral"):ti,ab,kw OR ("Left Hemisphere, Infarction, Cerebral"):ti,ab,kw OR ("Subcortical Infarction"):ti,ab,kw OR ("Infarction, Subcortical"):ti,ab,kw OR ("Infarctions, Subcortical"):ti,ab,kw OR ("Subcortical Infarctions"):ti,ab,kw OR ("Posterior Choroidal Artery Infarction"):ti,ab,kw OR ("Anterior Choroidal Artery Infarction"):ti,ab,kw OR ("Cerebral Infarction, Right Hemisphere"):ti,ab,kw OR ("Right Hemisphere, Cerebral Infarction"):ti,ab,kw OR ("Infarction, Right Hemisphere, Cerebral"):ti,ab,kw OR ("Right Hemisphere, Infarction, Cerebral"):ti,ab,kw OR ("Cerebral, Right Hemisphere, Infarction"):ti,ab,kw OR ("Infarction, Cerebral, Right Hemisphere"):ti,ab,kw OR ("Cerebral Hemorrhage"):ti,ab,kw OR ("Hemorrhage, Cerebrum"):ti,ab,kw OR ("Cerebrum Hemorrhage"):ti,ab,kw OR ("Cerebrum Hemorrhages"):ti,ab,kw OR ("Hemorrhages, Cerebrum"):ti,ab,kw OR ("Cerebral Parenchymal Hemorrhage"):ti,ab,kw OR ("Cerebral Parenchymal Hemorrhages"):ti,ab,kw OR ("Hemorrhage, Cerebral Parenchymal"):ti,ab,kw OR ("Hemorrhages, Cerebral Parenchymal"):ti,ab,kw OR ("Parenchymal Hemorrhage, Cerebral"):ti,ab,kw OR ("Parenchymal Hemorrhages, Cerebral"):ti,ab,kw OR ("Intracerebral Hemorrhage"):ti,ab,kw OR ("Hemorrhage, Intracerebral"):ti,ab,kw OR ("Hemorrhages, Intracerebral"):ti,ab,kw OR ("Intracerebral Hemorrhages"):ti,ab,kw OR ("Hemorrhage, Cerebral"):ti,ab,kw OR ("Cerebral Hemorrhages"):ti,ab,kw OR ("Hemorrhages, Cerebral"):ti,ab,kw OR ("Brain Hemorrhage, Cerebral"):ti,ab,kw OR ("Brain Hemorrhages, Cerebral"):ti,ab,kw OR ("Cerebral Brain Hemorrhage"):ti,ab,kw OR ("Cerebral Brain Hemorrhages"):ti,ab,kw OR ("Hemorrhage, Cerebral Brain"):ti,ab,kw OR ("Hemorrhages, Cerebral Brain"):ti,ab,kw OR ("Hemorrhagic Stroke"):ti,ab,kw OR ("Hemorrhagic Strokes"):ti,ab,kw OR ("Stroke, Hemorrhagic"):ti,ab,kw OR ("Subarachnoid Hemorrhagic Stroke"):ti,ab,kw OR ("Hemorrhagic Stroke, Subarachnoid"):ti,ab,kw OR ("Stroke, Subarachnoid Hemorrhagic"):ti,ab,kw OR ("Subarachnoid Hemorrhagic Strokes"):ti,ab,kw OR ("Intracerebral Hemorrhagic Stroke"):ti,ab,kw OR ("Hemorrhagic Stroke, Intracerebral"):ti,ab,kw OR ("Intracerebral Hemorrhagic Strokes"):ti,ab,kw OR ("Stroke, Intracerebral Hemorrhagic"):ti,ab,kw OR ("Intracerebral Hemorrhage Stroke"):ti,ab,kw OR ("Hemorrhage Stroke, Intracerebral"):ti,ab,kw OR ("Intracerebral Hemorrhage Strokes"):ti,ab,kw OR ("Stroke, Intracerebral Hemorrhage"):ti,ab,kw OR ("Ischemic Stroke"):ti,ab,kw OR ("Ischemic Strokes"):ti,ab,kw OR ("Stroke, Ischemic"):ti,ab,kw OR ("Ischaemic Stroke"):ti,ab,kw OR ("Ischaemic Strokes"):ti,ab,kw OR ("Stroke, Ischaemic"):ti,ab,kw OR ("Cryptogenic Ischemic Stroke"):ti,ab,kw OR ("Cryptogenic Ischemic Strokes"):ti,ab,kw OR ("Ischemic Stroke, Cryptogenic"):ti,ab,kw OR ("Stroke, Cryptogenic Ischemic"):ti,ab,kw OR ("Cryptogenic Stroke"):ti,ab,kw OR ("Cryptogenic Strokes"):ti,ab,kw OR ("Stroke, Cryptogenic"):ti,ab,kw OR ("Cryptogenic Embolism Stroke"):ti,ab,kw OR ("Cryptogenic Embolism Strokes"):ti,ab,kw OR ("Embolism Stroke, Cryptogenic"):ti,ab,kw OR ("Stroke, Cryptogenic Embolism"):ti,ab,kw OR ("Wake-up Stroke"):ti,ab,kw OR ("Stroke, Wake-up"):ti,ab,kw OR ("Wake up Stroke"):ti,ab,kw OR ("Wake-up Strokes"):ti,ab,kw OR ("Acute Ischemic Stroke"):ti,ab,kw OR ("Acute Ischemic Strokes"):ti,ab,kw OR ("Ischemic Stroke, Acute"):ti,ab,kw OR ("Stroke, Acute Ischemic"):ti,ab,kw OR ("Brain ischemia"):ti,ab,kw OR ("Brain Ischemias"):ti,ab,kw OR ("Ischemia, Brain"):ti,ab,kw OR ("Ischemic Encephalopathy"):ti,ab,kw OR ("Encephalopathy, Ischemic"):ti,ab,kw OR ("Ischemic Encephalopathies"):ti,ab,kw OR ("Cerebral Ischemia"):ti,ab,kw OR ("Cerebral Ischemias"):ti,ab,kw OR ("Ischemias, Cerebral"):ti,ab,kw OR ("Ischemia, Cerebral"):ti,ab,kw OR ("Brain Infarction"):ti,ab,kw OR ("Brain Infarctions"):ti,ab,kw OR ("Infarction, Brain"):ti,ab,kw OR ("Infarctions, Brain"):ti,ab,kw OR ("Brain Infarct"):ti,ab,kw OR ("Brain Infarcts"):ti,ab,kw OR ("Infarct, Brain"):ti,ab,kw OR ("Infarcts, Brain"):ti,ab,kw OR ("Brain Infarction, Posterior Circulation"):ti,ab,kw OR ("Infarction, Posterior Circulation, Brain"):ti,ab,kw OR ("Posterior Circulation Infarction, Brain"):ti,ab,kw OR ("Infarction, Brain, Posterior Circulation"):ti,ab,kw OR ("Posterior Circulation Brain Infarction"):ti,ab,kw OR ("Anterior Circulation Brain Infarction"):ti,ab,kw OR ("Infarction, Brain, Anterior Circulation"):ti,ab,kw OR ("Brain Infarction, Anterior Circulation"):ti,ab,kw OR ("Anterior Circulation Infarction, Brain"):ti,ab,kw OR ("Infarction, Anterior Circulation, Brain"):ti,ab,kw OR ("Venous Infarction, Brain"):ti,ab,kw OR ("Brain Venous Infarction"):ti,ab,kw OR ("Brain Venous Infarctions"):ti,ab,kw OR ("Infarction, Brain Venous"):ti,ab,kw OR ("Infarctions, Brain Venous"):ti,ab,kw OR ("Venous Infarctions, Brain"):ti,ab,kw OR ("Brain Infarction, Venous"):ti,ab,kw OR ("Brain Infarctions, Venous"):ti,ab,kw OR ("Infarction, Venous Brain"):ti,ab,kw OR ("Infarctions, Venous Brain"):ti,ab,kw OR ("Venous Brain Infarction"):ti,ab,kw OR ("Venous Brain Infarctions"):ti,ab,kw OR ("Anterior Cerebral Circulation Infarction"):ti,ab,kw OR ("Infarction, Anterior Cerebral Circulation"):ti,ab,kw OR ("Cerebrovascular Disorders"):ti,ab,kw OR ("Cerebrovascular Disorder"):ti,ab,kw OR ("Cerebrovascular Diseases"):ti,ab,kw OR ("Cerebrovascular Disease"):ti,ab,kw OR ("Disease, Cerebrovascular"):ti,ab,kw OR ("Diseases, Cerebrovascular"):ti,ab,kw OR ("Vascular Diseases, Intracranial"):ti,ab,kw OR ("Intracranial Vascular Disease"):ti,ab,kw OR ("Intracranial Vascular Diseases"):ti,ab,kw OR ("Vascular Disease, Intracranial"):ti,ab,kw OR ("Brain Vascular Disorders"):ti,ab,kw OR ("Brain Vascular Disorder"):ti,ab,kw OR ("Vascular Disorder, Brain"):ti,ab,kw OR ("Vascular Disorders, Brain"):ti,ab,kw OR ("Intracranial Vascular Disorders"):ti,ab,kw OR ("Intracranial Vascular Disorder"):ti,ab,kw OR ("Vascular Disorder, Intracranial"):ti,ab,kw OR ("Vascular Disorders, Intracranial"):ti,ab,kw OR ("Cerebrovascular Insufficiency"):ti,ab,kw OR ("Cerebrovascular Insufficiencies"):ti,ab,kw OR ("Insufficiencies, Cerebrovascular"):ti,ab,kw OR ("Insufficiency, Cerebrovascular"):ti,ab,kw OR ("Cerebrovascular Occlusion"):ti,ab,kw OR ("Cerebrovascular Occlusions"):ti,ab,kw OR ("Occlusion, Cerebrovascular"):ti,ab,kw OR ("Occlusions, Cerebrovascular"):ti,ab,kw OR ("CVA"):ti,ab,kw OR ("Intracranial Thromb*"):ti,ab,kw OR ("Ischemic Event "):ti,ab,kw OR ("Brain Thrombosis"):ti,ab,kw OR ("Cerebral Thrombus"):ti,ab,kw OR ("Ischemic Encephalopathies"):ti,ab,kw OR ("Ischemic Attack"):ti,ab,kw 82752

#18 #16 OR #17 85503

#19 #7 AND #18 1278

#20 ("Post stroke Epilepsy"):ti,ab,kw OR ("Poststroke Epilepsy"):ti,ab,kw 19

#21 #19 OR #20 1278

#22 MeSH descriptor: [Herbal Medicine] explode all trees 243

#23 MeSH descriptor: [Medicine, Chinese Traditional] explode all trees 1560

#24 MeSH descriptor: [Drugs, Chinese Herbal] explode all trees 4191

#25 MeSH descriptor: [Medicine, Traditional] explode all trees 1990

#26 #22 OR #23 OR #24 OR #25 5658

#27 ("Herbal Medicine"):ti,ab,kw OR ("Medicine, Herbal"):ti,ab,kw OR ("Hawaiian Herbal Medicine*"):ti,ab,kw OR ("Herbal Medicine*, Hawaiian"):ti,ab,kw OR ("Medicine*, Hawaiian Herbal"):ti,ab,kw OR ("La'au Lapa'au"):ti,ab,kw OR ("Laau Lapaau"):ti,ab,kw OR ("La au Lapa au"):ti,ab,kw OR ("Herbalism"):ti,ab,kw OR ("Medicine, Chinese Traditional"):ti,ab,kw OR ("Zhong Yi Xue"):ti,ab,kw OR ("Chung I Hsueh"):ti,ab,kw OR ("Hsueh, Chung I"):ti,ab,kw OR ("Chinese Medicine, Traditional"):ti,ab,kw OR ("Chinese Traditional Medicine"):ti,ab,kw OR ("Traditional Chinese Medicine"):ti,ab,kw OR ("Traditional Medicine, Chinese"):ti,ab,kw OR ("Traditional Tongue Diagnosis"):ti,ab,kw OR ("Tongue Diagnoses, Traditional"):ti,ab,kw OR ("Tongue Diagnosis, Traditional"):ti,ab,kw OR ("Traditional Tongue Diagnoses"):ti,ab,kw OR ("Traditional Tongue Assessment"):ti,ab,kw OR ("Tongue Assessment, Traditional"):ti,ab,kw OR ("Traditional Tongue Assessments"):ti,ab,kw OR ("Drugs, Chinese Herbal"):ti,ab,kw OR ("Chinese Drugs, Plant"):ti,ab,kw OR ("Chinese Herbal Drugs"):ti,ab,kw OR ("Herbal Drugs, Chinese"):ti,ab,kw OR ("Plant Extracts, Chinese"):ti,ab,kw OR ("Chinese Plant Extracts"):ti,ab,kw OR ("Extracts, Chinese Plant"):ti,ab,kw OR ("Medicine, Traditional"):ti,ab,kw OR ("Traditional Medicine"):ti,ab,kw OR ("Home Remed*"):ti,ab,kw OR ("Remed*, Home"):ti,ab,kw OR ("Medicine, Primitive"):ti,ab,kw OR ("Primitive Medicine"):ti,ab,kw OR ("Medicine, Folk"):ti,ab,kw OR ("Folk Medicine"):ti,ab,kw OR ("Medicine, Indigenous"):ti,ab,kw OR ("Indigenous Medicine"):ti,ab,kw OR ("Folk Remed*"):ti,ab,kw OR ("Remed*, Folk"):ti,ab,kw OR ("Ethnomedicine"):ti,ab,kw OR ("Chinese medicine"):ti,ab,kw OR ("TCM"):ti,ab,kw OR ("integrated chinese and western medicine "):ti,ab,kw OR ("decoction"):ti,ab,kw OR ("chinese patent medicine"):ti,ab,kw OR ("Chinese herbal medicine"):ti,ab,kw OR ("oriental medicine"):ti,ab,kw OR ("herbal formula"):ti,ab,kw OR ("herb*"):ti,ab,kw OR ("Alternative Medicine*"):ti,ab,kw OR ("Alternative Therap*"):ti,ab,kw OR ("Complementary Medicine*"):ti,ab,kw OR ("Complementary Therap*"):ti,ab,kw OR ("Integrat* Medicine*"):ti,ab,kw OR ("Integrat* Therap*"):ti,ab,kw 26999

#28 #26 OR #27 27340

#29 MeSH descriptor: [Randomized Controlled Trial] explode all trees 25732

#30 MeSH descriptor: [Randomized Controlled Trials as Topic] explode all trees 47397

#31 MeSH descriptor: [Controlled Clinical Trial] explode all trees 38477

#32 #29 OR #30 OR #31 85398

#33 (“Randomized Controlled Trial”):ti,ab,kw OR (“Randomized Controlled Trials as Topic”):ti,ab,kw OR (“Controlled Clinical Trial”):ti,ab,kw OR (“Randomized”):ti,ab,kw OR (“placebo”):ti,ab,kw OR (“randomly”):ti,ab,kw OR (“trial”):ti,ab,kw OR (“groups”):ti,ab,kw OR (“Clinical Trials”):ti,ab,kw OR (“Random”):ti,ab,kw OR (“RCT”):ti,ab,kw OR (“randomized controlled study”):ti,ab,kw OR (“randomized trial”):ti,ab,kw OR (“randomized study”):ti,ab,kw OR (“randomized placebo-controlled study”):ti,ab,kw OR (“randomized parallel-group study”):ti,ab,kw OR (“randomized placebo controlled”):ti,ab,kw OR (“randomized double-blin*”):ti,ab,kw 1541550

#34 #32 OR #33 1541581

#35 #21 AND #28 AND #34 10

n=10

**Web of Science**

# Web of Science 检索策略 (v0.1)

# 数据库: Web of Science 核心合集

# 权限:

- WOS.SSCI: 1980 to 2023

- WOS.AHCI: 1991 to 2023

- WOS.BHCI: 2005 to 2023

- WOS.ESCI: 2017 to 2023

- WOS.SCI: 1980 to 2023

- WOS.BSCI: 2005 to 2023

# 检索:

1: TS=(“Epilepsy” OR “Epilepsies” OR “Seizure Disorder” OR “Seizure Disorders” OR “Awakening Epilepsy” OR “Epilepsy, Awakening” OR “Epilepsy, Cryptogenic” OR “Cryptogenic Epilepsies” OR “Cryptogenic Epilepsy” OR “Epilepsies, Cryptogenic” OR “Aura” OR “Auras” OR “seizures” OR “Seizure” OR “Atonic Absence Seizures” OR “Atonic Absence Seizure” OR “Absence Seizure, Atonic” OR “Absence Seizures, Atonic” OR “Seizure, Atonic Absence” OR “Seizures, Sensory” OR “Seizure, Sensory” OR “Sensory Seizure” OR “Sensory Seizures” OR “Absence Seizures” OR “Petit Mal Convulsion” OR “Convulsion, Petit Mal” OR “Absence Seizure” OR “Seizure, Absence” OR “Convulsions” OR “Convulsion” OR “Convulsive Seizures” OR “Seizure, Convulsive” OR “Seizures, Convulsive” OR “Seizures, Motor” OR “Motor Seizure” OR “Motor Seizures” OR “Seizure, Motor” OR “Convulsive Seizure” OR “Jacksonian Seizure” OR “Seizure, Jacksonian” OR “Seizures, Auditory” OR “Auditory Seizure” OR “Auditory Seizures” OR “Seizure, Auditory” OR “Seizures, Focal” OR “Focal Seizure” OR “Focal Seizures” OR “Seizure, Focal” OR “Partial Seizures” OR “Partial Seizure” OR “Seizure, Partial” OR “Seizures, Generalized” OR “Generalized Seizure” OR “Generalized Seizures” OR “Seizure, Generalized” OR “Seizures, Gustatory” OR “Gustatory Seizure” OR “Gustatory Seizures” OR “Seizure, Gustatory” OR “Seizures, Olfactory” OR “Olfactory Seizure” OR “Olfactory Seizures” OR “Seizure, Olfactory” OR “Complex Partial Seizures” OR “Complex Partial Seizure” OR “Partial Seizure, Complex” OR “Partial Seizures, Complex” OR “Seizure, Complex Partial” OR “Single Seizure” OR “Seizure, Single” OR “Single Seizures” OR “Seizures, Somatosensory” OR “Seizure, Somatosensory” OR “Somatosensory Seizure” OR “Somatosensory Seizures” OR “Seizures, Vertiginous” OR “Seizure, Vertiginous” OR “Vertiginous Seizure” OR “Vertiginous Seizures” OR “Seizures, Vestibular” OR “Seizure, Vestibular” OR “Vestibular Seizure” OR “Vestibular Seizures” OR “Seizures, Visual” OR “Seizure, Visual” OR “Visual Seizure” OR “Visual Seizures” OR “Nonepileptic Seizures” OR “Non-Epileptic Seizures” OR “Non Epileptic Seizures” OR “Nonepileptic Seizure” OR “Seizure, Nonepileptic” OR “Seizures, Nonepileptic” OR “Non-Epileptic Seizure” OR “Non Epileptic Seizure” OR “Seizure, Non-Epileptic” OR “Generalized Absence Seizures” OR “Generalized Absence Seizure” OR “Absence Seizure, Generalized” OR “Absence Seizures, Generalized” OR “Seizure, Generalized Absence” OR “Tonic-Clonic Seizures” OR “Tonic Clonic Seizure” OR “Clonic Seizure, Tonic” OR “Clonic Seizures, Tonic” OR “Seizure, Tonic Clonic” OR “Tonic Clonic Seizures” OR “Generalized Tonic-Clonic Seizures” OR “Generalized Tonic Clonic Seizures” OR “Generalized Tonic-Clonic Seizure” OR “Seizure, Generalized Tonic-Clonic” OR “Seizures, Generalized Tonic-Clonic” OR “Tonic-Clonic Seizure, Generalized” OR “Tonic-Clonic Seizures, Generalized” OR “Seizures, Tonic-Clonic” OR “Tonic-Clonic Seizure” OR “Seizure, Tonic-Clonic” OR “Clonic Seizures” OR “Clonic Seizure” OR “Seizure, Clonic” OR “Seizures, Clonic” OR “Tonic Seizures” OR “Seizures, Tonic” OR “Tonic Seizure” OR “Seizure, Tonic” OR “Convulsion, Non-Epileptic” OR “Convulsion, Non Epileptic” OR “Convulsions, Non-Epileptic” OR “Non-Epileptic Convulsion” OR “Non-Epileptic Convulsions” OR “Atonic Seizures” OR “Atonic Seizure” OR “Seizure, Atonic” OR “Myoclonic Seizures” OR “Myoclonic Seizure” OR “Seizure, Myoclonic” OR “Epileptic Seizures” OR “Seizures, Epileptic” OR “Epileptic Seizure” OR “Seizure, Epileptic” OR “Epilepsy, Absence” OR “Absence Epilepsy” OR “Epilepsy, Petit Mal” OR “Petit Mal Epilepsy” OR “Akinetic Petit Mal” OR “Petit Mal, Akinetic” OR “Childhood Absence Epilepsy” OR “Absence Epilepsy, Childhood” OR “Epilepsy, Childhood Absence” OR “Pyknolepsy” OR “Pyknolepsies” OR “Pykno-Epilepsy” OR “Pykno Epilepsy” OR “Absence Seizure Disorder” OR “Absence Seizure Disorders” OR “Seizure Disorders, Absence” OR “Seizure Disorder, Absence” OR “Juvenile Absence Epilepsy” OR “Absence Epilepsy, Juvenile” OR “Epilepsy, Juvenile Absence” OR “Epilepsy Juvenile Absence” OR “Epilepsy, Absence, Atypical” OR “Epilepsy, Minor” OR “Minor Epilepsy” OR “Convulsion”) 运行日期: Wed Oct 11 2023 23:00:40 GMT+0800 (中国标准时间) 检索结果: 273522

2: TS=("Stroke" OR "Strokes" OR "Cerebrovascular Accident" OR "Cerebrovascular Accidents" OR "CVA (Cerebrovascular Accident)" OR "CVAs (Cerebrovascular Accident)" OR "Cerebrovascular Apoplexy" OR "Apoplexy, Cerebrovascular" OR "Vascular Accident, Brain" OR "Brain Vascular Accident" OR "Brain Vascular Accidents" OR "Vascular Accidents, Brain" OR "Cerebrovascular Stroke" OR "Cerebrovascular Strokes" OR "Stroke, Cerebrovascular" OR "Strokes, Cerebrovascular" OR "Apoplexy" OR "Cerebral Stroke" OR "Cerebral Strokes" OR "Stroke, Cerebral" OR "Strokes, Cerebral" OR "Stroke, Acute" OR "Acute Stroke" OR "Acute Strokes" OR "Strokes, Acute" OR "Cerebrovascular Accident, Acute" OR "Acute Cerebrovascular Accident" OR "Acute Cerebrovascular Accidents" OR "Cerebrovascular Accidents, Acute" OR "Cerebral Infarction" OR "Cerebral Infarctions" OR "Infarctions, Cerebral" OR "Infarction, Cerebral" OR "Cerebral Infarct" OR "Cerebral Infarcts" OR "Infarct, Cerebral" OR "Infarcts, Cerebral" OR "Cerebral Infarction, Left Hemisphere" OR "Left Hemisphere, Cerebral Infarction" OR "Infarction, Cerebral, Left Hemisphere" OR "Cerebral, Left Hemisphere, Infarction" OR "Infarction, Left Hemisphere, Cerebral" OR "Left Hemisphere, Infarction, Cerebral" OR "Subcortical Infarction" OR "Infarction, Subcortical" OR "Infarctions, Subcortical" OR "Subcortical Infarctions" OR "Posterior Choroidal Artery Infarction" OR "Anterior Choroidal Artery Infarction" OR "Cerebral Infarction, Right Hemisphere" OR "Right Hemisphere, Cerebral Infarction" OR "Infarction, Right Hemisphere, Cerebral" OR "Right Hemisphere, Infarction, Cerebral" OR "Cerebral, Right Hemisphere, Infarction" OR "Infarction, Cerebral, Right Hemisphere" OR "Cerebral Hemorrhage" OR "Hemorrhage, Cerebrum" OR "Cerebrum Hemorrhage" OR "Cerebrum Hemorrhages" OR "Hemorrhages, Cerebrum" OR "Cerebral Parenchymal Hemorrhage" OR "Cerebral Parenchymal Hemorrhages" OR "Hemorrhage, Cerebral Parenchymal" OR "Hemorrhages, Cerebral Parenchymal" OR "Parenchymal Hemorrhage, Cerebral" OR "Parenchymal Hemorrhages, Cerebral" OR "Intracerebral Hemorrhage" OR "Hemorrhage, Intracerebral" OR "Hemorrhages, Intracerebral" OR "Intracerebral Hemorrhages" OR "Hemorrhage, Cerebral" OR "Cerebral Hemorrhages" OR "Hemorrhages, Cerebral" OR "Brain Hemorrhage, Cerebral" OR "Brain Hemorrhages, Cerebral" OR "Cerebral Brain Hemorrhage" OR "Cerebral Brain Hemorrhages" OR "Hemorrhage, Cerebral Brain" OR "Hemorrhages, Cerebral Brain" OR "Hemorrhagic Stroke" OR "Hemorrhagic Strokes" OR "Stroke, Hemorrhagic" OR "Subarachnoid Hemorrhagic Stroke" OR "Hemorrhagic Stroke, Subarachnoid" OR "Stroke, Subarachnoid Hemorrhagic" OR "Subarachnoid Hemorrhagic Strokes" OR "Intracerebral Hemorrhagic Stroke" OR "Hemorrhagic Stroke, Intracerebral" OR "Intracerebral Hemorrhagic Strokes" OR "Stroke, Intracerebral Hemorrhagic" OR "Intracerebral Hemorrhage Stroke" OR "Hemorrhage Stroke, Intracerebral" OR "Intracerebral Hemorrhage Strokes" OR "Stroke, Intracerebral Hemorrhage" OR "Ischemic Stroke" OR "Ischemic Strokes" OR "Stroke, Ischemic" OR "Ischaemic Stroke" OR "Ischaemic Strokes" OR "Stroke, Ischaemic" OR "Cryptogenic Ischemic Stroke" OR "Cryptogenic Ischemic Strokes" OR "Ischemic Stroke, Cryptogenic" OR "Stroke, Cryptogenic Ischemic" OR "Cryptogenic Stroke" OR "Cryptogenic Strokes" OR "Stroke, Cryptogenic" OR "Cryptogenic Embolism Stroke" OR "Cryptogenic Embolism Strokes" OR "Embolism Stroke, Cryptogenic" OR "Stroke, Cryptogenic Embolism" OR "Wake-up Stroke" OR "Stroke, Wake-up" OR "Wake up Stroke" OR "Wake-up Strokes" OR "Acute Ischemic Stroke" OR "Acute Ischemic Strokes" OR "Ischemic Stroke, Acute" OR "Stroke, Acute Ischemic" OR "Brain ischemia" OR "Brain Ischemias" OR "Ischemia, Brain" OR "Ischemic Encephalopathy" OR "Encephalopathy, Ischemic" OR "Ischemic Encephalopathies" OR "Cerebral Ischemia" OR "Cerebral Ischemias" OR "Ischemias, Cerebral" OR "Ischemia, Cerebral" OR "Brain Infarction" OR "Brain Infarctions" OR "Infarction, Brain" OR "Infarctions, Brain" OR "Brain Infarct" OR "Brain Infarcts" OR "Infarct, Brain" OR "Infarcts, Brain" OR "Brain Infarction, Posterior Circulation" OR "Infarction, Posterior Circulation, Brain" OR "Posterior Circulation Infarction, Brain" OR "Infarction, Brain, Posterior Circulation" OR "Posterior Circulation Brain Infarction" OR "Anterior Circulation Brain Infarction" OR "Infarction, Brain, Anterior Circulation" OR "Brain Infarction, Anterior Circulation" OR "Anterior Circulation Infarction, Brain" OR "Infarction, Anterior Circulation, Brain" OR "Venous Infarction, Brain" OR "Brain Venous Infarction" OR "Brain Venous Infarctions" OR "Infarction, Brain Venous" OR "Infarctions, Brain Venous" OR "Venous Infarctions, Brain" OR "Brain Infarction, Venous" OR "Brain Infarctions, Venous" OR "Infarction, Venous Brain" OR "Infarctions, Venous Brain" OR "Venous Brain Infarction" OR "Venous Brain Infarctions" OR "Anterior Cerebral Circulation Infarction" OR "Infarction, Anterior Cerebral Circulation" OR "Cerebrovascular Disorders" OR "Cerebrovascular Disorder" OR "Cerebrovascular Diseases" OR "Cerebrovascular Disease" OR "Disease, Cerebrovascular" OR "Diseases, Cerebrovascular" OR "Vascular Diseases, Intracranial" OR "Intracranial Vascular Disease" OR "Intracranial Vascular Diseases" OR "Vascular Disease, Intracranial" OR "Brain Vascular Disorders" OR "Brain Vascular Disorder" OR "Vascular Disorder, Brain" OR "Vascular Disorders, Brain" OR "Intracranial Vascular Disorders" OR "Intracranial Vascular Disorder" OR "Vascular Disorder, Intracranial" OR "Vascular Disorders, Intracranial" OR "Cerebrovascular Insufficiency" OR "Cerebrovascular Insufficiencies" OR "Insufficiencies, Cerebrovascular" OR "Insufficiency, Cerebrovascular" OR "Cerebrovascular Occlusion" OR "Cerebrovascular Occlusions" OR "Occlusion, Cerebrovascular" OR "Occlusions, Cerebrovascular" OR "CVA" OR "Intracranial Thromb*" OR "Ischemic Event " OR "Brain Thrombosis" OR "Cerebral Thrombus" OR "Ischemic Encephalopathies" OR "Ischemic Attack") 运行日期: Wed Oct 11 2023 23:02:03 GMT+0800 (中国标准时间) 检索结果: 532264

3: #1 AND #2 运行日期: Wed Oct 11 2023 23:02:32 GMT+0800 (中国标准时间) 检索结果: 14346

4: TS=("Post stroke Epilepsy" OR "Poststroke Epilepsy") 运行日期: Wed Oct 11 2023 23:03:06 GMT+0800 (中国标准时间) 检索结果: 399

5: #3 OR #4 运行日期: Wed Oct 11 2023 23:03:55 GMT+0800 (中国标准时间) 检索结果: 14377

6: TS=("Herbal Medicine" OR "Medicine, Herbal" OR "Hawaiian Herbal Medicine*" OR "Herbal Medicine*, Hawaiian" OR "Medicine*, Hawaiian Herbal" OR "La'au Lapa'au" OR "Laau Lapaau" OR "La au Lapa au" OR "Herbalism" OR "Medicine, Chinese Traditional" OR "Zhong Yi Xue" OR "Chung I Hsueh" OR "Hsueh, Chung I" OR "Chinese Medicine, Traditional" OR "Chinese Traditional Medicine" OR "Traditional Chinese Medicine" OR "Traditional Medicine, Chinese" OR "Traditional Tongue Diagnosis" OR "Tongue Diagnoses, Traditional" OR "Tongue Diagnosis, Traditional" OR "Traditional Tongue Diagnoses" OR "Traditional Tongue Assessment" OR "Tongue Assessment, Traditional" OR "Traditional Tongue Assessments" OR "Drugs, Chinese Herbal" OR "Chinese Drugs, Plant" OR "Chinese Herbal Drugs" OR "Herbal Drugs, Chinese" OR "Plant Extracts, Chinese" OR "Chinese Plant Extracts" OR "Extracts, Chinese Plant" OR "Medicine, Traditional" OR "Traditional Medicine" OR "Home Remed*" OR "Remed*, Home" OR "Medicine, Primitive" OR "Primitive Medicine" OR "Medicine, Folk" OR "Folk Medicine" OR "Medicine, Indigenous" OR "Indigenous Medicine" OR "Folk Remed*" OR "Remed*, Folk" OR "Ethnomedicine" OR "Chinese medicine" OR "TCM" OR "integrated chinese and western medicine " OR "decoction" OR "chinese patent medicine" OR "Chinese herbal medicine" OR "oriental medicine" OR "herbal formula" OR "herb*" OR "Alternative Medicine*" OR "Alternative Therap*" OR "Complementary Medicine*" OR "Complementary Therap*" OR "Integrat* Medicine*" OR "Integrat* Therap*") 运行日期: Wed Oct 11 2023 23:04:11 GMT+0800 (中国标准时间) 检索结果: 413637

7: TS=(“Randomized Controlled Trial” OR “randomized controlled trial (topic) ” OR “controlled clinical trial” OR “Randomized” OR “placebo” OR “randomly” OR “trial” OR “groups” OR “Clinical Trials” OR “Random” OR “RCT” OR “randomized controlled study” OR “randomized trial” OR “randomized study” OR “randomized placebo-controlled study” OR “randomized parallel-group study” OR “randomized placebo controlled” OR “randomized double-blind”) 运行日期: Wed Oct 11 2023 23:04:27 GMT+0800 (中国标准时间) 检索结果: 5956034

8: #5 AND #6 AND #7 运行日期: Wed Oct 11 2023 23:04:54 GMT+0800 (中国标准时间) 检索结果: 23

n=23

**Chinese Clinical Trial Register**

n=0

**ClinicalTrials.gov**

n=0

## Supplementary File S2. The PRISMA checklist of this meta-analysis

| **Section and Topic** | **Item #** | **Checklist item** | **Location where item is reported** |
| --- | --- | --- | --- |
| **TITLE** | | |  |
| Title | 1 | Identify the report as a systematic review. | P1 |
| **ABSTRACT** | | |  |
| Abstract | 2 | See the PRISMA 2020 for Abstracts checklist. | P1-2 |
| **INTRODUCTION** | | |  |
| Rationale | 3 | Describe the rationale for the review in the context of existing knowledge. | P2 |
| Objectives | 4 | Provide an explicit statement of the objective(s) or question(s) the review addresses. | P2 |
| **METHODS** | | |  |
| Eligibility criteria | 5 | Specify the inclusion and exclusion criteria for the review and how studies were grouped for the syntheses. | P3-4 |
| Information sources | 6 | Specify all databases, registers, websites, organisations, reference lists and other sources searched or consulted to identify studies. Specify the date when each source was last searched or consulted. | P3 |
| Search strategy | 7 | Present the full search strategies for all databases, registers and websites, including any filters and limits used. | P3 and Supplementary File S1 |
| Selection process | 8 | Specify the methods used to decide whether a study met the inclusion criteria of the review, including how many reviewers screened each record and each report retrieved, whether they worked independently, and if applicable, details of automation tools used in the process. | P3 |
| Data collection process | 9 | Specify the methods used to collect data from reports, including how many reviewers collected data from each report, whether they worked independently, any processes for obtaining or confirming data from study investigators, and if applicable, details of automation tools used in the process. | P4 |
| Data items | 10a | List and define all outcomes for which data were sought. Specify whether all results that were compatible with each outcome domain in each study were sought (e.g. for all measures, time points, analyses), and if not, the methods used to decide which results to collect. | P4 |
|  | 10b | List and define all other variables for which data were sought (e.g. participant and intervention characteristics, funding sources). Describe any assumptions made about any missing or unclear information. | P4 |
| Study risk of bias assessment | 11 | Specify the methods used to assess risk of bias in the included studies, including details of the tool(s) used, how many reviewers assessed each study and whether they worked independently, and if applicable, details of automation tools used in the process. | P4 |
| Effect measures | 12 | Specify for each outcome the effect measure(s) (e.g. risk ratio, mean difference) used in the synthesis or presentation of results. | P4 |
| Synthesis methods | 13a | Describe the processes used to decide which studies were eligible for each synthesis (e.g. tabulating the study intervention characteristics and comparing against the planned groups for each synthesis (item #5)). | P4 |
|  | 13b | Describe any methods required to prepare the data for presentation or synthesis, such as handling of missing summary statistics, or data conversions. | P4 |
|  | 13c | Describe any methods used to tabulate or visually display results of individual studies and syntheses. | P4 |
|  | 13d | Describe any methods used to synthesize results and provide a rationale for the choice(s). If meta-analysis was performed, describe the model(s), method(s) to identify the presence and extent of statistical heterogeneity, and software package(s) used. | P4 |
|  | 13e | Describe any methods used to explore possible causes of heterogeneity among study results (e.g. subgroup analysis, meta-regression). | P4 |
|  | 13f | Describe any sensitivity analyses conducted to assess robustness of the synthesized results. | P4 |
| Reporting bias assessment | 14 | Describe any methods used to assess risk of bias due to missing results in a synthesis (arising from reporting biases). | P4 |
| Certainty assessment | 15 | Describe any methods used to assess certainty (or confidence) in the body of evidence for an outcome. | P4 |
| **RESULTS** | | |  |
| Study selection | 16a | Describe the results of the search and selection process, from the number of records identified in the search to the number of studies included in the review, ideally using a flow diagram. | P5 and Figure 1 |
|  | 16b | Cite studies that might appear to meet the inclusion criteria, but which were excluded, and explain why they were excluded. | Supplementary Table S1 |
| Study characteristics | 17 | Cite each included study and present its characteristics. | P5 and Table1 |
| Risk of bias in studies | 18 | Present assessments of risk of bias for each included study. | P8 and Figure 2 |
| Results of individual studies | 19 | For all outcomes, present, for each study: (a) summary statistics for each group (where appropriate) and (b) an effect estimate and its precision (e.g. confidence/credible interval), ideally using structured tables or plots. | P8-P12 and Figure3-7 |
| Results of syntheses | 20a | For each synthesis, briefly summarise the characteristics and risk of bias among contributing studies. | P8-P12 and Figure3-7 |
|  | 20b | Present results of all statistical syntheses conducted. If meta-analysis was done, present for each the summary estimate and its precision (e.g. confidence/credible interval) and measures of statistical heterogeneity. If comparing groups, describe the direction of the effect. | P8-P12 and Figure3-7 |
|  | 20c | Present results of all investigations of possible causes of heterogeneity among study results. | P8-P12 and Figure3-7 |
|  | 20d | Present results of all sensitivity analyses conducted to assess the robustness of the synthesized results. | P8-P12 and Figure3-7 |
| Reporting biases | 21 | Present assessments of risk of bias due to missing results (arising from reporting biases) for each synthesis assessed. | P12 |
| Certainty of evidence | 22 | Present assessments of certainty (or confidence) in the body of evidence for each outcome assessed. | P12 and Table3 |
| **DISCUSSION** | | |  |
| Discussion | 23a | Provide a general interpretation of the results in the context of other evidence. | P15 |
|  | 23b | Discuss any limitations of the evidence included in the review. | P15-P17 |
|  | 23c | Discuss any limitations of the review processes used. | P15-P17 |
|  | 23d | Discuss implications of the results for practice, policy, and future research. | P15-P17 |
| **OTHER INFORMATION** | | |  |
| Registration and protocol | 24a | Provide registration information for the review, including register name and registration number, or state that the review was not registered. | P3 |
|  | 24b | Indicate where the review protocol can be accessed, or state that a protocol was not prepared. | P3 |
|  | 24c | Describe and explain any amendments to information provided at registration or in the protocol. | P3 |
| Support | 25 | Describe sources of financial or non-financial support for the review, and the role of the funders or sponsors in the review. | P18 |
| Competing interests | 26 | Declare any competing interests of review authors. | P18 |
| Availability of data, code and other materials | 27 | Report which of the following are publicly available and where they can be found: template data collection forms; data extracted from included studies; data used for all analyses; analytic code; any other materials used in the review. | P18 |

*From:*  Page MJ, McKenzie JE, Bossuyt PM, Boutron I, Hoffmann TC, Mulrow CD, et al. The PRISMA 2020 statement: an updated guideline for reporting systematic reviews. BMJ 2021;372:n71. doi: 10.1136/bmj.n71

For more information, visit: <http://www.prisma-statement.org/>
